# Causal effect between systemic inflammatory cytokines and osteoporotic pathological fractures: A bidirectional Mendelian randomization study

**DOI:** 10.1097/MD.0000000000044636

**Published:** 2025-10-31

**Authors:** Heyuan Wang, Dan Wang

**Affiliations:** a Department of Hepatic-Biliary Surgery, The First Hospital of China Medical University, Shenyang, China; b Operation Room, The First Hospital of China Medical University, Shenyang, China.

**Keywords:** causal inference, inflammatory factors, Mendelian randomization, osteoporosis, osteoporotic pathological fractures

## Abstract

Osteoporosis is a prevalent metabolic bone disorder that significantly impairs patients’ quality of life. Mounting evidence suggests a close relationship between systemic inflammation and bone metabolism, yet the causal nature of this association remains unclear. This study aims to elucidate potential causal links between circulating inflammatory factors and osteoporotic pathological fractures. We employed a bidirectional 2-sample Mendelian randomization (MR) approach, utilizing large-scale genetic data from the FinnGen biobank (1822 osteoporosis cases and 3,11,210 controls) and the genome-wide association study catalog to analyze 91 circulating inflammatory factors. Instrumental variables were selected using a threshold of *P* < 1 × 10^−5^, followed by stringent linkage disequilibrium pruning (*R*^2^ < 0.001). The inverse variance weighted method served as the primary analytical tool, supplemented by MR-Egger, weighted median, and mode-based estimator methods. Sensitivity analyses, including leave-one-out analysis, Mendelian Randomization Pleiotropy RESidual Sum and Outlier, and Cochran’s *Q* test, were conducted to assess the robustness of the results. Forward MR analysis identified potential causal associations between 7 inflammatory factors and the risk of osteoporotic pathological fractures. Notably, Artemin levels were negatively correlated with fracture risk (OR = 0.7954, *P* = .0167), while elevated levels of β-nerve growth factor (OR = 1.2375, *P* = .0398), C-X-C motif chemokine 10 (OR = 1.2675, *P* = .0183), CXCL6 (OR = 1.2623, *P* = .0026), interleukin-10 receptor α subunit (OR = 1.2828, *P* = .0204), interleukin-10 receptor β subunit (OR = 1.1463, *P* = .0386), and latency-associated peptide transforming growth factor β1 (OR = 1.2481, *P* = .0206) were associated with increased fracture risk. Reverse MR analysis suggested that fractures might lead to decreased levels of C-X-C motif chemokine 11 (OR = 0.9574, *P* = .0104), interleukin-1α (OR = 0.9591, *P* = .0263), and thymic stromal lymphopoietin (OR = 0.9625, *P* = .0439), as well as elevated levels of tumor necrosis factor-β (OR = 1.0519, *P* = .0111). This study unveils a complex bidirectional relationship between circulating inflammatory factors and osteoporotic pathological fractures. These findings provide novel insights into the pathogenesis of osteoporosis and offer important clues for potential preventive, diagnostic, and therapeutic strategies.

**Key Points**

1. This study identified potential causal associations between 7 inflammatory factors and the risk of osteoporotic pathological fractures, provided novel insights into the pathogenesis of osteoporosis and offered important clues for potential preventive, diagnostic, and therapeutic strategies.
2. Artemin levels were negatively correlated with fracture risk, while elevated levels of β-NGF, CXCL10, CXCL6, IL-10Rα, IL-10Rβ, and LAP-TGFβ1 were associated with increased fracture risk.
3. Fractures might lead to decreased levels of CXCL11, IL-1α and TSLP, as well as elevated levels of TNF-β.

## 1. Introduction

Osteoporosis is a prevalent metabolic bone disorder characterized by low bone mass and microarchitectural deterioration, significantly increasing fracture risk.^[1]^ Globally, osteoporosis causes over 8.9 million fractures annually, with 1 in 3 women over 50 experiencing an osteoporotic fracture.^[2]^ The male-to-female ratio for osteoporotic fractures is approximately 1:1.6, with women accounting for 80% of forearm fractures, 75% of humeral fractures, 70% of hip fractures, and 58% of spine fractures.^[3]^ Pathological fractures not only severely impair patients’ quality of life but also markedly increase mortality rates. One study reported a 20% to 24% mortality rate within 1 year following hip fractures.^[4]^ The economic burden of osteoporosis and its complications on healthcare systems is substantial. In the United States, direct medical costs for osteoporosis are estimated between $13.7 and $20.3 billion, projected to reach $25.3 billion by 2025.^[5]^ In Europe, this figure is even higher at €37 billion, with over 70% related to osteoporotic fracture costs.^[6]^ Furthermore, functional recovery postfracture is often incomplete, with less than half of elderly patients fully regaining walking ability and functional independence within a year, and about 40% to 60% unable to return to their previous functional status.^[7]^ Notably, the risk of subsequent fractures increases significantly after an initial osteoporotic fracture, with the risk in the first year being 2.7 times higher than in patients without a fracture history.^[8]^ Given the aging population trend, with the proportion of individuals aged 65 and over expected to rise from 17.7% to 24.2% by 2040, the disease burden of osteoporosis is anticipated to intensify, necessitating more effective prevention and treatment strategies.^[9]^

Recent evidence increasingly suggests a close connection between systemic inflammation and bone metabolism. Inflammatory cytokines, including members of the tumor necrosis factor (TNF) family, interleukin (IL) family, interferons (IFN), and chemokines, are thought to contribute to osteoporosis pathogenesis by influencing osteoclast and osteoblast activity.^[10]^ Huang et al found that high blood levels of soluble IL-6 receptor were strongly associated with reduced bone mineral density and bone strength in the femoral neck.^[11]^ Studies on chronic inflammatory disease patients support this association, with rheumatoid arthritis patients showing a 2 to 3-fold higher incidence of osteoporosis compared to the general population.^[12]^ However, current understanding primarily stems from observational studies, which are limited in their ability to exclude confounding factors and establish causality. Therefore, there is an urgent need for more rigorous research methods, such as Mendelian randomization (MR) analysis, to explore potential causal links between inflammatory cytokines and osteoporosis. While Zheng’s study analyzed the correlation between inflammatory factors and fractures at different sites,^[13]^ there remains a significant gap between normal fractures and pathological fractures, with vastly different clinical treatment strategies. Consequently, assessing the causal relationship of inflammation in pathological fractures is crucial. However, similar large-scale studies on other inflammatory factors are still lacking, representing an important gap for future research to address.

To overcome the limitations of traditional observational studies, this research employed a bidirectional MR approach to investigate the causal relationship between systemic inflammatory cytokines and osteoporotic pathological fractures. The MR method used genetic variants as instrumental variables, simulating randomized trials and effectively controlling for confounding factors and reverse causality. Our primary research objective is to determine causal associations between specific inflammatory cytokines and pathological fracture risk, while also exploring whether pathological fractures, in turn, influence systemic inflammation levels. Through this bidirectional analysis, our study systematically evaluated the causality and directionality of this relationship for the first time, filling a crucial gap in existing research.

We utilized large-scale genome-wide association study (GWAS) data, including inflammatory marker and bone density measurements from hundreds of thousands of participants, ensuring the reliability of statistical inferences. The findings of this study can help inform future clinical strategies and public health approaches aimed at preventing osteoporotic fractures. If a causal relationship between inflammatory cytokines and osteoporosis is confirmed, it will provide strong support for developing new prevention and treatment strategies, such as targeted interventions for specific inflammatory pathways. Simultaneously, these findings could transform osteoporosis risk assessment methods, suggesting the inclusion of inflammatory markers in screening processes. From a public health perspective, the results of this study may prompt policymakers to place greater emphasis on the prevention and control of chronic inflammation as a key strategy for reducing the burden of osteoporosis. In summary, this study not only advances our understanding of osteoporosis etiology but also provides clear directions for future clinical research and public health interventions.

## 2. Materials and methods

### 2.1. Data sources

This study utilized 2 primary data sources: the OSTEOPOROSIS_FRACTURE_FG sample from the Finnish FinnGen biobank database (https://www.finngen.fi/en), comprising 1822 osteoporosis cases and 3,11,210 control samples, and GWAS data for 91 circulating inflammatory factors, originally from the Protein Research Division of the Department of Public Health and Primary Care at the University of Cambridge, now accessible in the GWAS Catalog (ID range: GCST90274758 to GCST90274848).^[14]^ The FinnGen biobank is part of a large-scale genomics and personalized medicine research project that has collected and analyzed genome and health data from 5,00,000 Finnish biobank donors. This extensive database combines genome information with digital healthcare data from national health registries, providing a comprehensive resource for studying genetic factors in various diseases, including osteoporosis. Both datasets include subjects of European ancestry, ensuring genetic background consistency and providing a reliable foundation for investigating potential causal relationships between inflammatory factors and osteoporosis using MR analysis. Because these data come from separate cohorts, sample overlap is expected to be minimal, reducing the risk of bias. Nevertheless, we applied sensitivity analyses to further address any potential bias from unknown overlap or population stratification.

### 2.2. Selection of instrumental variables

To assess the causal relationship between inflammatory factors and osteoporotic pathological fractures, we employed a 2-sample MR analysis method. This method is based on 3 key assumptions: the selected instrumental variables (genetic variants) must be closely associated with blood inflammatory factors; genetic variants must be independent of any potential confounding factors; and genetic variants must influence osteoporotic pathological fractures only through inflammatory factors, without any other direct causal pathways.^[15]^

We selected instrumental variables from the GWAS summary data of inflammatory factors using a significance threshold of *P* < 1 × 10⁻⁵ to ensure a sufficient number of single nucleotide polymorphisms (SNPs) for subsequent analysis, particularly for cytokines with few genome-wide significant variants. While this relaxed threshold can increase statistical power, we acknowledged that it may also raise the risk of including false-positive associations or weak instruments. To mitigate these risks, we applied stringent linkage disequilibrium (LD) pruning using PLINK software, with a physical distance threshold of 10,000 kb and an R² threshold of 0.001, applying the same LD pruning procedure consistently to both exposure and outcome datasets to exclude potentially highly correlated SNPs. Furthermore, we evaluated the strength of each instrument by calculating the *F*-statistic, excluding SNPs with *F* < 10 to minimize weak instrument bias. This step ensured that the selected instrumental variables had sufficient statistical power to explain the variation in the exposure variable. Through this series of rigorous screening processes, we not only improved the reliability of causal inference but also helped reduce potential biases and false-positive results. Supplementary File 1 and Table S1, Supplemental Digital Content, https://links.lww.com/MD/Q387 summarized the number of SNPs retained for each inflammatory factor and for fractures after applying GWAS significance threshold, LD pruning, and *F*-statistic filtering.

### 2.3. MR analysis methods

#### 2.3.1. Main analysis methods

This study employed multiple MR methods to comprehensively evaluate the causal relationship between blood inflammatory factors and osteoporotic pathological fractures. We used 5 different MR methods: inverse variance weighted (IVW), MR-Egger regression, weighted median (WM), simple mode, and weighted mode. Each method has its unique advantages and assumptions. The IVW method assumes all instrumental variables are valid and provides the highest statistical power; MR-Egger regression can detect and correct for horizontal pleiotropy; the WM method can provide consistent estimates even with a small proportion of invalid instrumental variables; while the Mode methods perform well when the majority of instrumental variables are valid. Considering the robustness and widespread application of the IVW method in causal inference, we chose it as the primary method for estimating causal effects, while using other methods for supplementary analysis and result validation.^[16]^

Given that this study analyzed 91 circulating inflammatory factors for their causal associations with osteoporotic pathological fractures, strict control for multiple comparisons was applied using Bonferroni correction. The significance threshold was adjusted to 0.05 divided by 91 to reduce false-positive findings. Only results passing this corrected threshold were considered statistically significant.

#### 2.3.2. Sensitivity analysis

To assess the robustness and reliability of MR results, we conducted a series of sensitivity analyses. First, we used the Leave-one-out cross-validation to evaluate the influence of individual SNPs on the overall causal effect estimate. This method helps identify outliers that might exert undue influence on the results by excluding 1 SNP at a time and recalculating the effect estimate. Second, we employed the Mendelian Randomization Pleiotropy RESidual Sum and Outlier (MR-PRESSO) method to detect and correct for horizontal pleiotropy bias. MR-PRESSO improves the accuracy of causal estimates by identifying and removing outlier SNPs. Additionally, we used Cochran’s *Q* test to assess the heterogeneity of causal effect estimates, with high heterogeneity potentially indicating the presence of invalid instrumental variables or effect modification. Finally, we utilized the intercept term from MR-Egger regression to test for directional pleiotropy bias, where a significant deviation of the intercept from 0 might indicate systematic pleiotropy.^[17]^

#### 2.3.3. Bidirectional MR analysis

To further explore the causal relationship between blood inflammatory factors and osteoporotic pathological fractures, we conducted bidirectional MR analysis. In the forward analysis, we used inflammatory factors as the exposure and fractures as the outcome; in the reverse analysis, we treated fractures as the exposure and inflammatory factors as the outcome. For the reverse MR analysis, we selected inflammatory significantly associated with osteoporotic pathological fractures from the FinnGen biobank database as instrumental variables. The selection criteria were the same as those used in the forward analysis, including *P*-value threshold, LD pruning, and *F*-statistic evaluation. In the bidirectional MR analysis, we also used the IVW method as the primary causal effect estimation method, supplemented by MR-Egger, WM, simple mode, and weighted mode methods. We conducted the same sensitivity analyses as in the forward MR analysis to assess the robustness of the results. This bidirectional analysis approach helped us understand more comprehensively the complex relationship between inflammatory factors and fracture risk, and detect potential reverse causation or bidirectional causal effects.

All MR analyses were performed using R language (version 4.4.1) and related R packages. The main packages used include “TwoSampleMR,” “MRInstruments,” “ieugwasr,” “purrr,” and “pacman,” among others.

## 3. Results

**Figure 1.**
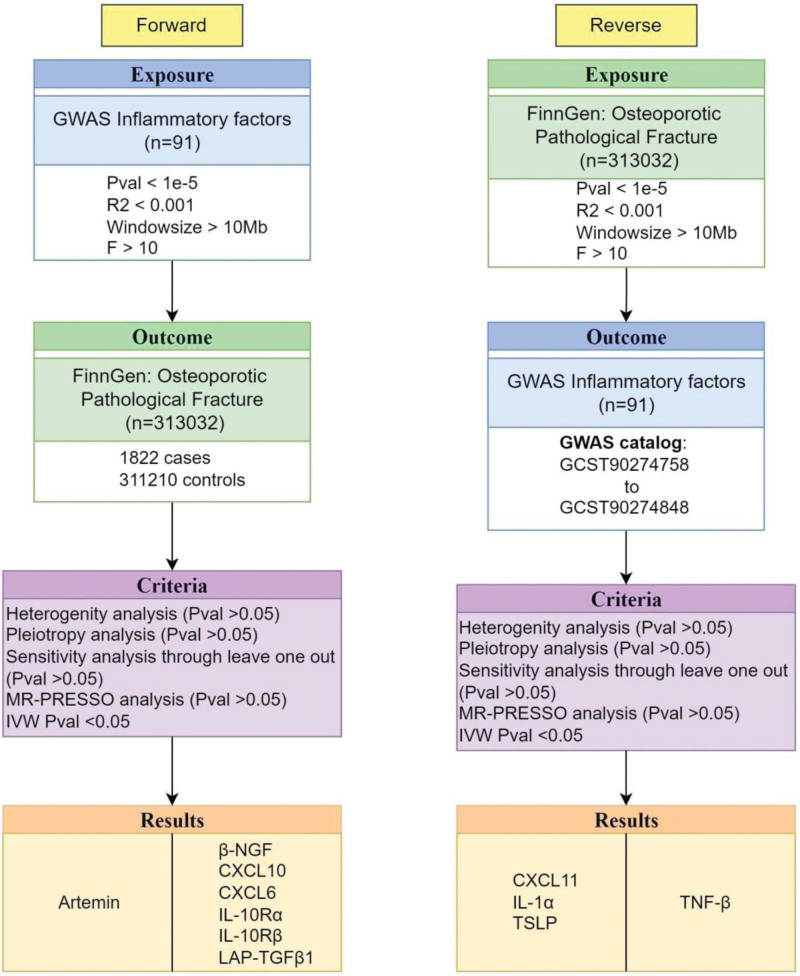
Flowchart of this study. β-NGF = β-nerve growth factor, CXCL10 = C-X-C motif chemokine 10, CXCL6 = C-X-C motif chemokine 6, IL-10Rα = interleukin-10 receptor α subunit, IL-10Rβ = interleukin-10 receptor β subunit, LAP-TGFβ1 = latency-associated peptide transforming growth factor β1.

The main flow and results of this study are shown in Figure 1.

### 3.1. Causal relationship assessment between inflammatory factors and osteoporotic pathological fractures

**Figure 2.**
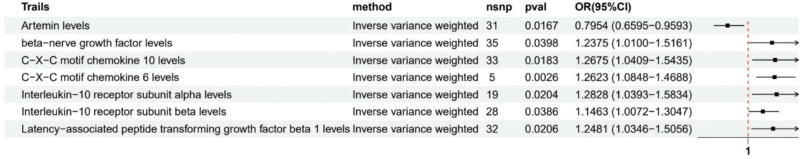
Forest map of MR results of Artemin, β-NGF, XCL10, CXCL6, IL-10Rα, IL-10Rβ, and LAP-TGFβ1 indicated significant statistical inflammatory protein levels and osteoporotic pathological fractures. β-NGF = β-nerve growth factor, CXCL6 = C-X-C motif chemokine 6, IL-10Rα = interleukin-10 receptor α subunit, IL-10Rβ = interleukin-10 receptor β subunit, LAP-TGFβ1 = latency-associated peptide transforming growth factor β1, MR = Mendelian randomization.

We used a 2-sample MR method to evaluate the causal relationship between various circulating inflammatory factors and osteoporotic pathological fractures. Preliminary results showed that among all analyzed inflammatory factors, 7 factors demonstrated potential causal associations with the risk of osteoporotic pathological fractures (Fig. 2). Through IVW analysis, we found that Artemin levels were negatively correlated with fracture risk (OR = 0.7954, 95% CI: 0.6595–0.9593, *P* = .0167), suggesting that decreased Artemin levels may increase fracture risk. Conversely, elevated levels of 6 other inflammatory factors appeared to be associated with increased fracture risk. Specifically, β-nerve growth factor (β-NGF; OR = 1.2375, 95% CI: 1.0100–1.5161, *P* = .0398), C-X-C motif chemokine 10 (CXCL10; OR = 1.2675, 95% CI: 1.0409–1.5435, *P* = .0183), C-X-C motif chemokine 6 (CXCL6; OR = 1.2623, 95% CI: 1.0848–1.4688, *P* = .0026), interleukin-10 receptor α subunit (IL-10Rα; OR = 1.2828, 95% CI: 1.0393–1.5834, *P* = .0204), interleukin-10 receptor β subunit (IL-10Rβ; OR = 1.1463, 95% CI: 1.0072–1.3047, *P* = .0386), and latency-associated peptide transforming growth factor β1 (LAP-TGFβ1; OR = 1.2481, 95% CI: 1.0346–1.5056, *P* = .0206) all showed potential causal associations between elevated levels and increased fracture risk. Notably, CXCL6 showed the most significant correlation (*P* = .0026), which may suggest its important role in the pathogenesis of osteoporosis. These findings provided new perspectives on how inflammatory processes may influence the development of osteoporosis, while also offering clues for potential therapeutic targets.

These results suggested that decreased levels of Artemin and increased levels of β-NGF, CXCL10, CXCL6, IL-10Rα, IL-10Rβ, and LAP-TGFβ1 may have causal associations with increased risk of osteoporotic pathological fractures. To further verify the reliability of these associations, we conducted heterogeneity and pleiotropy tests for each factor. Preliminary analysis showed that these associations did not exhibit significant heterogeneity or horizontal pleiotropy, which enhances the credibility of our findings. (Table 1 and Supplementary Files 2–8, Supplemental Digital Content, https://links.lww.com/MD/Q387).

**Table 1:** Heterogeneity, pleiotropy, and MR-PRESSO test results from bidirectional 2-sample MR analysis of Artemin, β-NGF, CXCL10, CXCL6, IL-10Rα, IL-10Rβ, and LAP-TGFβ, and their association with osteoporotic pathological fractures.

ID	Reported trait	Symbol	*Q*_IVW	*Q*_*P*val_IVW	*Q*_Egger	*Q*_*P*val_Egger	Pleiotropy_pval	PRESSO_*P*val
GCST90274760	Artemin levels	ARTN	29.026	0.516	28.641	0.484	0.539	0.523
GCST90274762	beta-nerve growth factor levels	Beta-NGF	26.876	0.802	26.876	0.765	0.986	0.825
GCST90274780	C-X-C motif chemokine 10 levels	CXCL10	39.469	0.171	35.852	0.251	0.087	0.183
GCST90274783	C-X-C motif chemokine 6 levels	CXCL6	1.818	0.769	1.328	0.722	0.535	0.661
GCST90274796	Interleukin-10 receptor subunit alpha levels	IL-10	13.14	0.783	12.892	0.743	0.625	0.806
GCST90274797	Interleukin-10 receptor subunit beta levels	IL-10RA	29.578	0.333	25.108	0.513	0.044	0.381
GCST90274818	Latency-associated peptide transforming growth factor beta 1 levels	LAP TGF-beta-1	33.628	0.341	33.201	0.314	0.539	0.359

β-NGF = β-nerve growth factor, CXCL10 = C-X-C motif chemokine 10, CXCL6 = C-X-C motif chemokine 6, IL-10Rα = interleukin-10 receptor α subunit, IL-10Rβ = interleukin-10 receptor β subunit, IVW = inverse variance weighted, LAP-TGFβ1 = latency-associated peptide transforming growth factor β1, MR = Mendelian randomization, MR-PRESSO = Mendelian Randomization Pleiotropy RESidual Sum and Outlier

### 3.2. Causal relationship assessment of osteoporotic pathological fractures on circulating inflammatory factors

To explore potential bidirectional relationships between osteoporotic pathological fractures and circulating inflammatory factors, we conducted reverse MR analysis, using osteoporotic pathological fractures as the exposure and 91 circulating inflammatory factors as outcomes. On 1 hand, we aimed to verify whether previously identified significant inflammatory factors showed reverse causality, and on the other hand, we sought to identify and explore potential causal effects of osteoporotic pathological fractures on a broader group of circulating inflammatory factors. Following the inclusion and screening criteria outlined in the methods section, 18 SNPs were retained for osteoporotic pathological fractures as the exposure.

**Figure 3.**
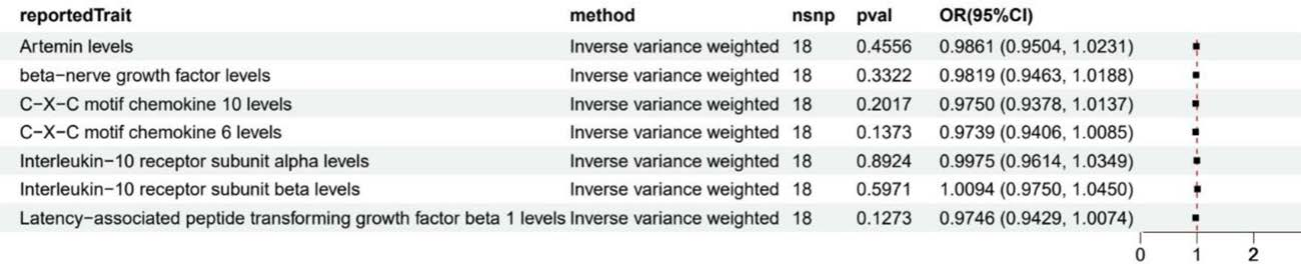
Forest plot of reverse MR analysis of Artemin, β-NGF, XCL10, CXCL6, IL-10Rα, IL-10Rβ, and LAP-TGFβ1 showed the impact of osteoporotic pathological fractures on circulating inflammatory factor levels. β-NGF = β-nerve growth factor, CXCL6 = C-X-C motif chemokine 6, IL-10Rα = interleukin-10 receptor α subunit, IL-10Rβ = interleukin-10 receptor β subunit, LAP-TGFβ1 = latency-associated peptide transforming growth factor β1, MR = Mendelian randomization.

Results of the reverse MR analysis showed that the 7 inflammatory factors identified in Section 3.1 (Artemin, β-NGF, CXCL10, CXCL6, IL-10Rα, IL-10Rβ, and LAP-TGFβ1) did not demonstrate significant reverse causal relationships in this analysis (Fig. 3), further supporting their unidirectional causal influence on osteoporotic pathological fractures.

**Figure 4.**
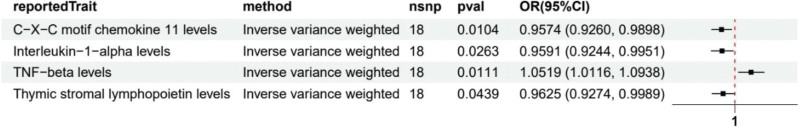
Forest plot of reverse MR analysis of CXCL11, IL-1α, TNF-β and TSLP showed the impact of osteoporotic pathological fractures on circulating inflammatory factor levels. CXCL11 = C-X-C motif chemokine 11, IL-1α = interleukin-1α, MR = Mendelian randomization, TNF-β = tumor necrosis factor-β, TSLP = thymic stromal lymphopoietin.

Moreover, osteoporotic pathological fractures showed statistically significant causal associations with levels of 4 circulating inflammatory factors (Fig. 4). These include negative correlations between fracture risk and levels of C-X-C motif chemokine 11 (CXCL11; OR = 0.9574, 95% CI: 0.9260–0.9898, *P* = .0104), interleukin-1α (IL-1α; OR = 0.9591, 95% CI: 0.9244–0.9951, *P* = .0263), and thymic stromal lymphopoietin (TSLP; OR = 0.9625, 95% CI: 0.9274–0.9989, *P* = .0439), as well as a positive correlation with tumor necrosis factor-β (TNF-β) levels (OR = 1.0519, 95% CI: 1.0116–1.0938, *P* = .0111).

These results suggested that osteoporotic pathological fractures may lead to changes in levels of specific inflammatory factors. Notably, except for TNF-β, the other 3 inflammatory factors (CXCL11, IL-1α, and TSLP) showed decreasing trends, while TNF-β levels showed an increasing trend. Further results of heterogeneity, pleiotropy, and MR-PRESSO tests are shown in Table 2. Analysis results indicate that statistically significant associations between inflammatory factors and osteoporotic pathological fractures do not exhibit significant heterogeneity or pleiotropy (Supplementary Files 9–12, Supplemental Digital Content, https://links.lww.com/MD/Q387).

**Table 2:** Heterogeneity, pleiotropy, and MR-PRESSO test results from bidirectional 2-sample MR analysis of osteoporotic pathological fractures and associations with inflammatory cytokines CXCL11, IL-1α, TNF-β and TSLP.

ID	Outcome traits	Symbol	*Q*_IVW	*Q*_*P*val_IVW	*Q*_Egger	*Q*_*P*val_Egger	Pleiotropy_pval	PRESSO_*P*val
GCST90274781	C-X-C motif chemokine 11 levels	CXCL11	9.554	0.921	16	0.946	0.242	0.93
GCST90274805	Interleukin-1-alpha levels	IL-1α	11.864	0.808	11.54	0.775	0.577	0.835
GCST90274840	TNF-beta levels	TNFB	18.86	0.337	16.366	0.428	0.138	0.394
GCST90274845	Thymic stromal lymphopoietin levels	TSLP	12.711	0.755	12.575	0.704	0.717	0.751

CXCL11 = C-X-C motif chemokine 11, IL-1α = interleukin-1α, IVW = inverse variance weighted, MR = Mendelian randomization, TNF-β = tumor necrosis factor-β, TSLP = thymic stromal lymphopoietin, MR-PRESSO = Mendelian Randomization Pleiotropy RESidual Sum and Outlier

These findings revealed complex interactions between osteoporotic pathological fractures and specific circulating inflammatory factors. While certain inflammatory factors may be potential causes of osteoporotic pathological fractures themselves might also induce changes in levels of specific inflammatory factors. This bidirectional analysis approach provided us with a more comprehensive understanding of disease mechanisms, which may have important implications for future treatment strategy development.

Results of sensitivity analyses, including heterogeneity (Cochran’s *Q* and *P*-value), MR-Egger intercept and *P*-value, pleiotropy test, and MR-PRESSO global test, are summarized for all significant associations in Table 3. Full details of the sensitivity analyses are provided in the Supplementary Materials, Supplemental Digital Content, https://links.lww.com/MD/Q387.

**Table 3:** Summary of sensitivity analyses for significant MR associations between inflammatory factors and osteoporotic pathological fractures.

ID	*Q*_IVW	*Q*_IVW_P	Egger_intercept	Egger_intercept_P	PRESSO_global_*P*
GCST90274760	29.026	0.516	0.012900	0.5390	1
GCST90274762	26.876	0.802	-0.000423	0.9860	1
GCST90274780	39.469	0.171	-0.030900	0.0868	1
GCST90274783	1.818	0.769	0.021300	0.5350	1
GCST90274796	13.140	0.783	-0.010300	0.6250	1
GCST90274797	29.578	0.333	-0.029600	0.0442	1
GCST90274818	33.628	0.341	0.011500	0.5390	1

IVW = inverse variance weighted, MR = Mendelian randomization

In addition, we have provided summary statistics for the *F*-statistics of instrumental variables for each inflammatory factor in the supplementary material (Supplementary File 13 and Table S2, Supplemental Digital Content, https://links.lww.com/MD/Q387). This included the mean, median, minimum, and maximum *F*-statistics for the SNPs used as instruments in the MR analysis, reflecting the strength of the selected instruments.

## 4. Discussion

This study employed bidirectional MR methods to investigate potential causal relationships between circulating inflammatory factors and osteoporotic pathological fractures. We conducted a comprehensive analysis of 91 circulating inflammatory factors using large-scale genetic data from the FinnGen biobank and GWAS Catalog. The results revealed complex and interesting associations between inflammatory factors and osteoporotic pathological fractures. In the forward MR analysis, we found potential causal associations between 7 inflammatory factors and the risk of osteoporotic pathological fractures. Among these, Artemin levels showed a negative correlation with fracture risk, while elevated levels of β-NGF, CXCL10, CXCL6, IL-10Rα, IL-10Rβ, and LAP-TGFβ1 were associated with increased fracture risk. These findings provided new perspectives on understanding how inflammatory processes influence the development of osteoporosis, while also offering clues for potential therapeutic targets. Notably, further analysis of these 7 factors showed no significant heterogeneity or horizontal pleiotropy, and MR-PRESSO tests did not observe significant bias in the causal relationships, further supporting the robustness of these associations.

In the reverse MR analysis, we found that the significant inflammatory factors identified in the forward analysis (such as Artemin, β-NGF, CXCL10, etc) did not show significant reverse causal relationships. This finding further supported the hypothesis of a unidirectional causal influence of these inflammatory factors on osteoporotic pathological fractures. Additionally, osteoporotic pathological fractures may affect the levels of 4 circulating inflammatory factors. Specifically, fractures may lead to decreased levels of CXCL11, IL-1α, and TSLP, and increased levels of TNF-β. These results revealed that fractures themselves may cause changes in specific inflammatory factor levels, highlighting the bidirectional relationship between osteoporosis and inflammation. Moreover, in terms of heterogeneity, pleiotropy, and MR-PRESSO tests, the associations between the 4 inflammatory factors in the reverse analysis and osteoporotic pathological fractures showed no significant heterogeneity or pleiotropy.

Among the 7 inflammatory factors included in the forward MR analysis, Artemin and β-NGF, as neurotrophic factors, may influence bone metabolism by regulating skeletal innervation. For example, a study on early postmenopausal osteoporosis patients found that serum β-NGF levels were negatively correlated with bone density, and increased levels were positively correlated with fracture risk.^[18]^ This suggested that β-NGF may be a potential biomarker for osteoporosis progression. Interestingly, it is a protective factor in traumatic fractures in young people.^[13]^ CXCL10 and CXCL6 belong to the chemokine family and may influence bone remodeling processes by regulating immune cell recruitment and bone cell activity.^[19–21]^ IL-10Rα and IL-10Rβ, as receptors for the anti-inflammatory cytokine IL-10, may influence bone metabolism by regulating inflammatory responses.^[22]^ Studies have shown that IL-10 gene polymorphisms are associated with bone density in postmenopausal women, suggesting the importance of the IL-10 signaling pathway in bone metabolism.^[23,24]^ LAP-TGFβ1 is the latent form of TGF-β1 and plays a key role in bone remodeling.^[25]^ Compared to previous studies, our MR analysis not only confirmed some known associations between inflammatory factors (such as CXCL10) and osteoporosis but also identified some new potential targets (such as Artemin). These findings provided new directions for the prevention and treatment of osteoporosis. For example, targeted therapies for these inflammatory factors may become new strategies for future osteoporosis treatment. Furthermore, the levels of these inflammatory factors may be used to develop new risk assessment tools to help identify high-risk populations early.

The reverse MR analysis in this study revealed 4 inflammatory factors that may be affected by fractures: CXCL11, IL-1α, TNF-β, and TSLP. These findings provided new perspectives for understanding postfracture inflammatory responses and their impact on the healing process. CXCL11, as a chemokine, plays an important role in immune cell recruitment.^[26]^ IL-1α is a crucial pro-inflammatory cytokine. Studies have shown that IL-1α levels increase in the early stages after fracture, suggesting that IL-1α may play a key role in early fracture repair.^[27]^ TNF-β (also known as lymphotoxin-α) plays an important role in inflammation and immune responses. A study of 47 fracture patients showed significantly elevated TNF-β levels after fracture.^[28]^ This sustained inflammatory response may have complex effects on the fracture healing process. TSLP is a cytokine associated with allergies and inflammation.^[29,30]^ Changes in the levels of these inflammatory factors may significantly impact fracture healing and subsequent complications. For example, moderate elevation of CXCL11 and IL-1α levels may help promote early repair responses, but excessive or prolonged elevation may lead to chronic inflammation, affecting healing quality.

Comparing forward and reverse MR results, we found a complex bidirectional relationship between inflammation and osteoporotic pathological fractures. Forward MR results suggested that certain inflammatory factors (such as CXCL10) may increase fracture risk, while reverse MR results showed that fractures themselves can cause changes in inflammatory factor levels (such as CXCL11). This complex interaction highlighted the central role of inflammation in bone metabolism and fracture repair.

The findings of this study have important clinical and public health implications for the prevention, diagnosis, treatment, and management of osteoporosis. Firstly, in terms of osteoporosis risk assessment, the 7 inflammatory factors we identified (Artemin, β-NGF, CXCL10, CXCL6, IL-10Rα, IL-10Rβ, LAP-TGFβ1) may become new biomarkers. Integrating these markers into existing risk assessment tools, such as the fracture risk assessment tool (FRAX) model, could significantly improve the accuracy of fracture risk prediction.^[31]^ For example, a study based on genomic medicine sequencing results combined with the FRAX model showed that incorporating a whole-genome polygenic risk score into the FRAX assessment model could refine the risk prediction with a positive net reclassification index ranging from 0.024 to 0.072.^[32]^ This improvement helps clinicians identify high-risk populations earlier, enabling early intervention and thus reducing fracture incidence. In terms of public health policy making, the results of this study emphasized the importance of inflammation in bone health, providing a basis for developing more comprehensive osteoporosis prevention strategies. Consideration could be given to including inflammatory marker screening in routine health checks for middle-aged and elderly populations.^[33]^ A longitudinal study spanning 20 years showed that inflammatory markers including CXCL10 and IL-10 are specific aging factors, therefore, early screening of inflammatory factors can be used to assess age-related changes including pathological fractures, improving quality-adjusted life years. This screening strategy could become an important component of future osteoporosis and pathological fracture prevention.

This study explored potential causal relationships between circulating inflammatory factors and osteoporotic pathological fractures through innovative methods, providing important new insights in this field. However, several limitations should be considered when interpreting the results. Firstly, the sample is mainly from European populations, which may limit the generalizability of the findings and raises concerns about population stratification. Secondly, although the MR approach can control for confounding to some extent, it still relies on key assumptions such as the validity of instrumental variables and the absence of horizontal pleiotropy. While sensitivity analyses (e.g., MR-Egger regression, WM) were conducted to detect and account for pleiotropy, residual unbalanced pleiotropy cannot be fully excluded, which may affect the reliability of the results. In particular, for certain inflammatory factors with few genome-wide significant SNPs, a relaxed significance threshold (*P* < 1 × 10⁻⁵) was used, which may increase the risk of false positives or weak instrument bias; however, this risk was mitigated through stringent LD pruning, exclusion of SNPs with *F*-statistics < 10, and multiple sensitivity analyses. Measurement error in cytokine quantification could attenuate causal estimates, and the finite sample size available for reverse MR analysis may reduce statistical power in detecting reverse causality. Moreover, MR estimates reflect average lifetime genetic effects and cannot capture temporal changes or the dynamic relationship between cytokine levels and fracture risk over time, possibly masking important time-dependent effects. Despite these limitations, this study demonstrated significant innovation. The bidirectional MR design not only explored the impact of inflammatory factors on osteoporotic pathological fractures risk but also examined the potential impact of osteoporotic pathological fractures on inflammatory factor levels, providing a more comprehensive picture of causal relationships. To our knowledge, this large-scale screening of 91 circulating inflammatory factors and osteoporotic pathological fractures is the most comprehensive to date, laying a foundation for a systematic understanding of inflammation’s role in osteoporotic pathological fractures. Although the observed odds ratio of 1.09 indicates a modest increase in individual risk, such an effect could translate into a substantial public health impact given the high prevalence of osteoporosis in the aging population.

These findings have some implications at both theoretical and practical levels. Theoretically, the study deepens our understanding of the role of inflammation in the pathogenesis of osteoporosis, particularly revealing new potential causal relationships, such as the protective effect of Artemin. This provides new directions for research into bone metabolism regulatory mechanisms. Practically, the research results offer new insights for risk assessment, prevention, and treatment of osteoporosis. The identified inflammatory factors may become new biomarkers or therapeutic targets, potentially improving clinical management strategies for osteoporosis.

This study points to several important directions for future work. Firstly, functional validation experiments, including in vitro and in vivo studies, are needed to further verify and elucidate the discovered causal relationships, especially to conduct in-depth research on the mechanisms of newly discovered inflammatory factors (such as Artemin) in bone metabolism. Secondly, designing and conducting targeted clinical trials to evaluate the effectiveness and safety of new risk assessment tools or treatment strategies based on the findings of this study, for example, assessing the clinical effect of incorporating CXCL10 and β-NGF into fracture risk assessment models. Moreover, replicating this study in different ethnic and regional populations to verify the universality of the results and explore possible racial differences, which is of great significance for the development of global osteoporosis prevention and control strategies.

Furthermore, conducting long-term longitudinal studies to explore the dynamic relationship between inflammatory factor levels and osteoporosis progression may help better understand the natural course and progression mechanisms of the disease. Combining the results of this study with other omics data (such as transcriptomics, proteomics) is expected to provide a more comprehensive picture of the molecular mechanisms of osteoporosis. Screening existing drug libraries based on the inflammatory factors discovered in this study to find potential candidate drugs for osteoporosis prevention or treatment, this drug repositioning strategy may accelerate the development of new treatment methods. Finally, using machine learning algorithms to integrate the inflammatory markers discovered in this study with other clinical and imaging features to develop more accurate fracture risk prediction models, which may significantly improve the early diagnosis and personalized management of osteoporosis.

## 5. Conclusion

In summary, this study employed a bidirectional 2-sample MR method to systematically explore the potential causal relationships between 91 circulating inflammatory factors and osteoporotic pathological fractures, revealing a series of important findings. We identified 7 inflammatory factors significantly associated with the risk of osteoporotic pathological fractures. Among them, Artemin showed a protective effect, while elevated levels of β-NGF, CXCL10, CXCL6, IL-10Rα, IL-10Rβ, and LAP-TGFβ1 were associated with increased risk of osteoporotic pathological fractures. The reverse analysis results further supported the hypothesis of unidirectional causal influence of these inflammatory factors on fracture risk, while also revealing that fractures themselves may affect the levels of certain inflammatory factors. Specifically, fractures may lead to decreased levels of CXCL11, IL-1α, and TSLP, and increased levels of TNF-β. These associations demonstrated good robustness in heterogeneity, pleiotropy, and MR-PRESSO tests, supporting their potential as causal factors. These research findings highlight the complex bidirectional relationship between osteoporotic pathological fractures and inflammation.

## Author contributions

**Conceptualization:** Heyuan Wang, Dan Wang.

**Data curation:** Dan Wang.

**Formal analysis:** Dan Wang.

**Validation:** Heyuan Wang.

**Writing – original draft:** Dan Wang.

**Writing – review & editing:** Heyuan Wang.

## Supplementary Material


