# Supplementary material for: Causal effect between systemic inflammatory cytokines and osteoporotic pathological fractures: A bidirectional Mendelian randomization study
Source: Medicine (Baltimore). 2025 Oct 31;104(44):e44636. doi: 10.1097/MD.0000000000044636 (PMC12582674; doi:10.1097/MD.0000000000044636)
Supplement: Supplementary file 1 [file medi-104-e44636-s001.pdf]

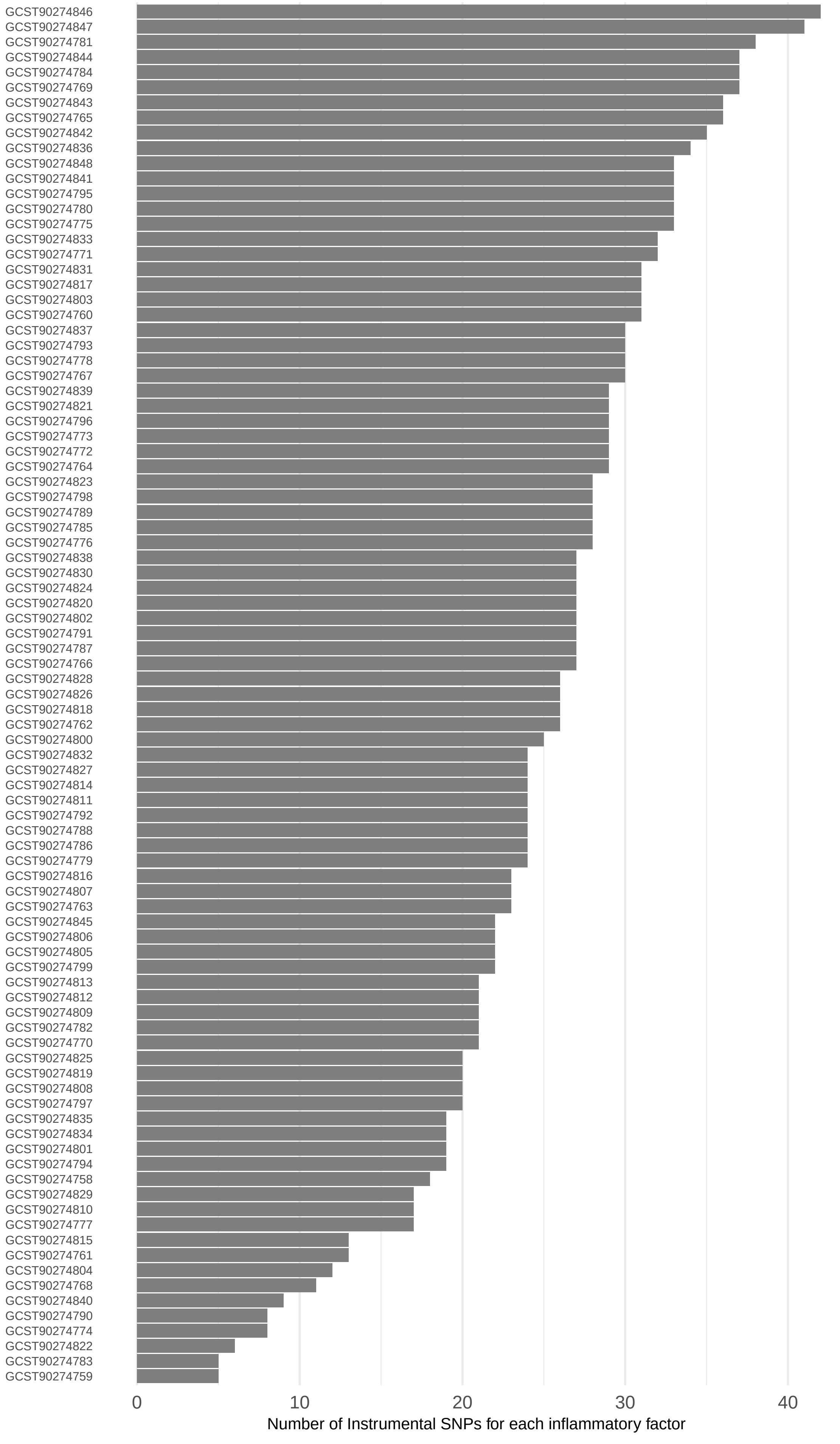

GCST90274846
GCST90274847
GCST90274781
GCST90274844
GCST90274784
GCST90274769
GCST90274843
GCST90274765
GCST90274842
GCST90274836
GCST90274848
GCST90274841
GCST90274795
GCST90274780
GCST90274775
GCST90274833
GCST90274771
GCST90274831
GCST90274817
GCST90274803
GCST90274760
GCST90274837
GCST90274793
GCST90274778
GCST90274767
GCST90274839
GCST90274821
GCST90274796
GCST90274773
GCST90274772
GCST90274764
GCST90274823
GCST90274798
GCST90274789
GCST90274785
GCST90274776
GCST90274838
GCST90274830
GCST90274824
GCST90274820
GCST90274802
GCST90274791
GCST90274787
GCST90274766
GCST90274828
GCST90274826
GCST90274818
GCST90274762
GCST90274800
GCST90274832
GCST90274827
GCST90274814
GCST90274811
GCST90274792
GCST90274788
GCST90274786
GCST90274779
GCST90274816
GCST90274807
GCST90274763
GCST90274845
GCST90274806
GCST90274805
GCST90274799
GCST90274813
GCST90274812
GCST90274809
GCST90274782
GCST90274770
GCST90274825
GCST90274819
GCST90274808
GCST90274797
GCST90274835
GCST90274834
GCST90274801
GCST90274794
GCST90274758
GCST90274829
GCST90274810
GCST90274777
GCST90274815
GCST90274761
GCST90274804
GCST90274768
GCST90274840
GCST90274790
GCST90274774
GCST90274822
GCST90274783
GCST90274759
0
10
20
30
40
Number of Instrumental SNPs for each inflammatory factor

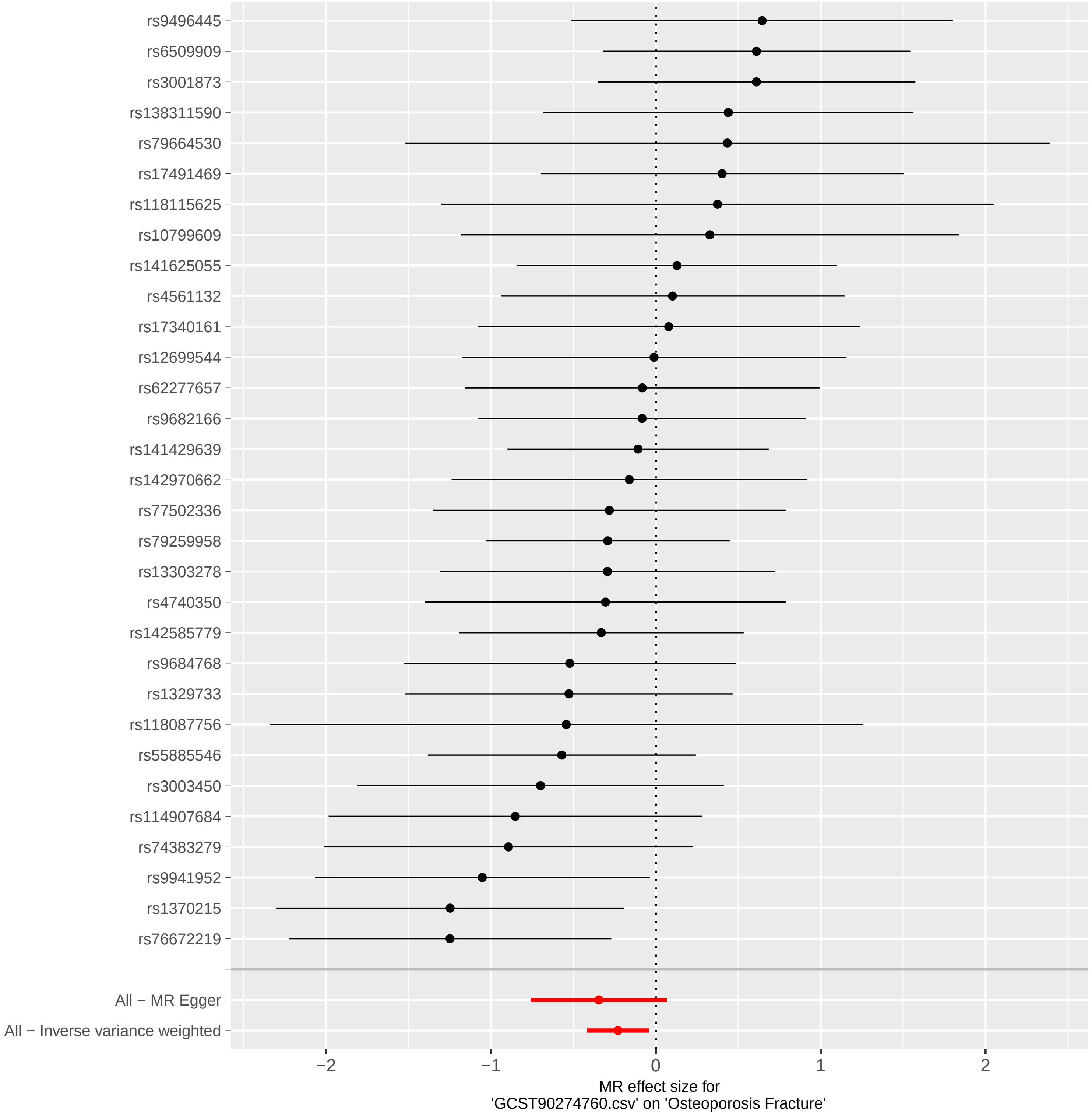
rs9496445
rs6509909
rs3001873
rs138311590
rs79664530
rs17491469
rs118115625
rs10799609
rs141625055
rs4561132
rs17340161
rs12699544
rs62277657
rs9682166
rs141429639
rs142970662
rs77502336
rs79259958
rs13303278
rs4740350
rs142585779
rs9684768
rs1329733
rs118087756
rs55885546
rs3003450
rs114907684
rs74383279
rs9941952
rs1370215
rs76672219
All − MR Egger
All − Inverse variance weighted
−2
−1
0
1
2
MR effect size for
'GCST90274760.csv' on 'Osteoporosis Fracture'

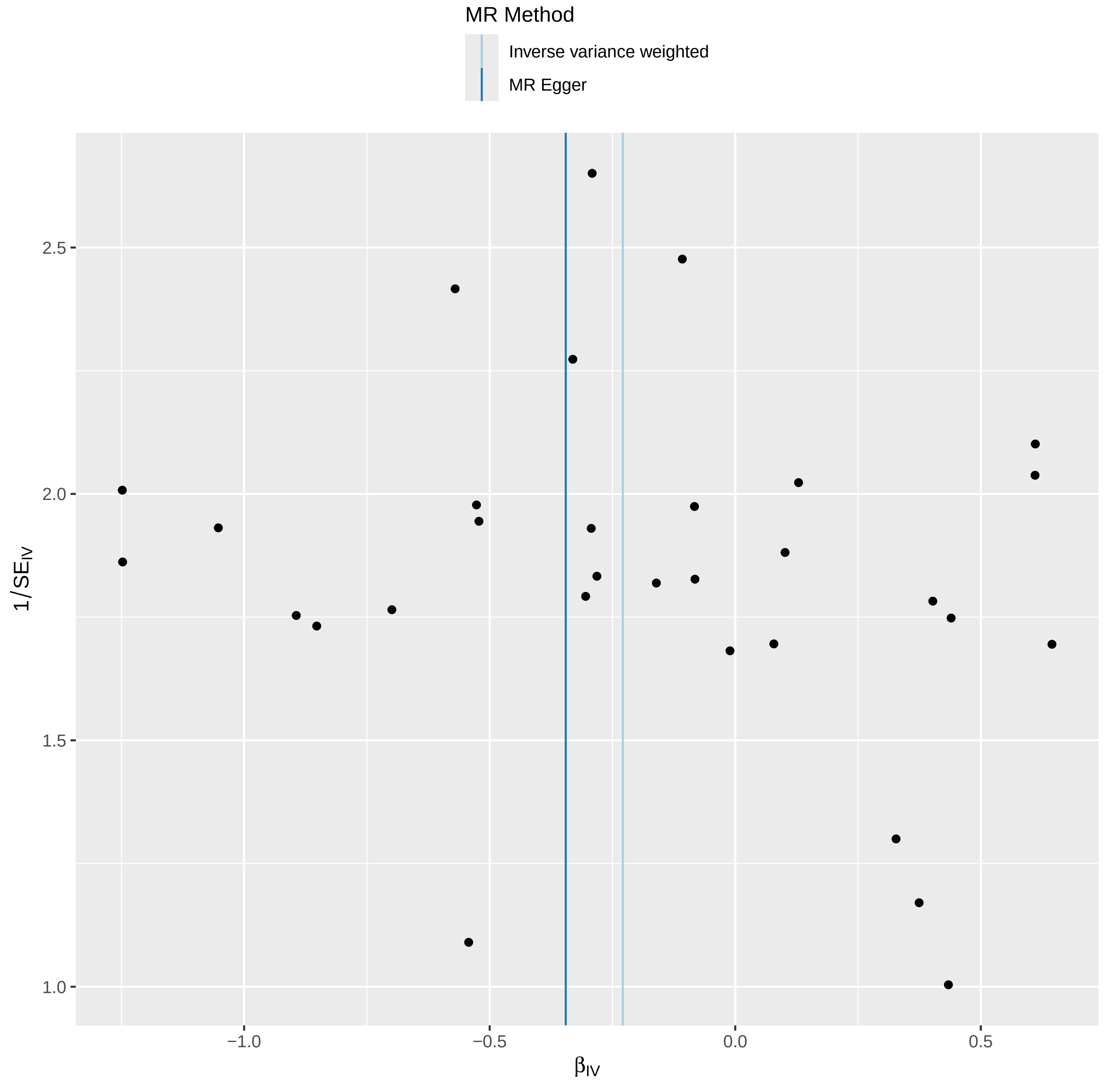

MR Method
Inverse variance weighted
MR Egger
2.5
2.0
1.5
1.0
1/SE_IV
−1.0
−0.5
0.0
0.5
β_IV

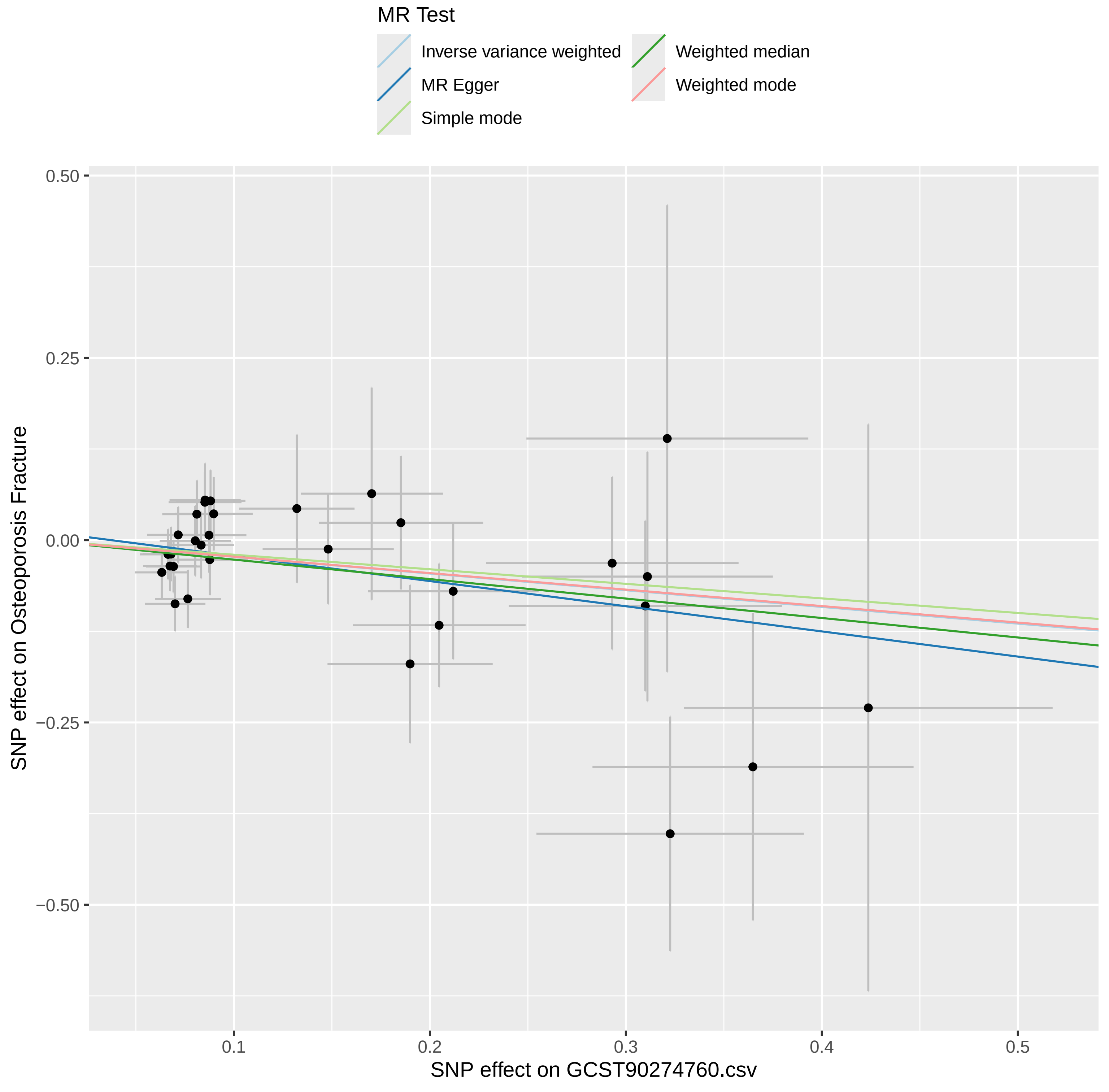

MR Test
Inverse variance weighted
MR Egger
Simple mode
Weighted median
Weighted mode
0.50
0.25
0.00
−0.25
−0.50
SNP effect on Osteoporosis Fracture
0.1
0.2
0.3
0.4
0.5
SNP effect on GCST90274760.csv

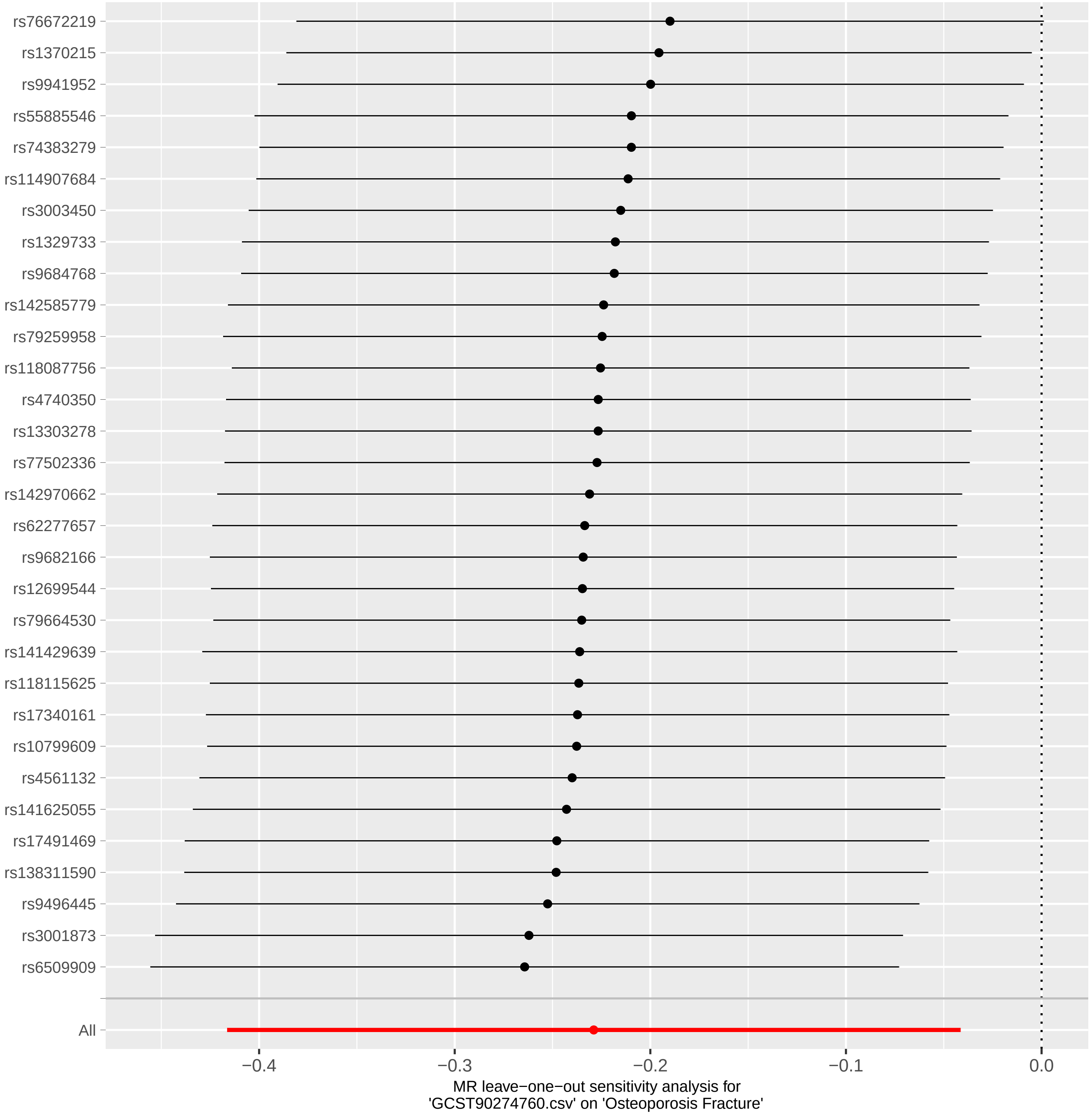
rs76672219
rs1370215
rs9941952
rs55885546
rs74383279
rs114907684
rs3003450
rs1329733
rs9684768
rs142585779
rs79259958
rs118087756
rs4740350
rs13303278
rs77502336
rs142970662
rs62277657
rs9682166
rs12699544
rs79664530
rs141429639
rs118115625
rs17340161
rs10799609
rs4561132
rs141625055
rs17491469
rs138311590
rs9496445
rs3001873
rs6509909
All
−0.4
−0.3
−0.2
−0.1
0.0
MR leave−one−out sensitivity analysis for
'GCST90274760.csv' on 'Osteoporosis Fracture'

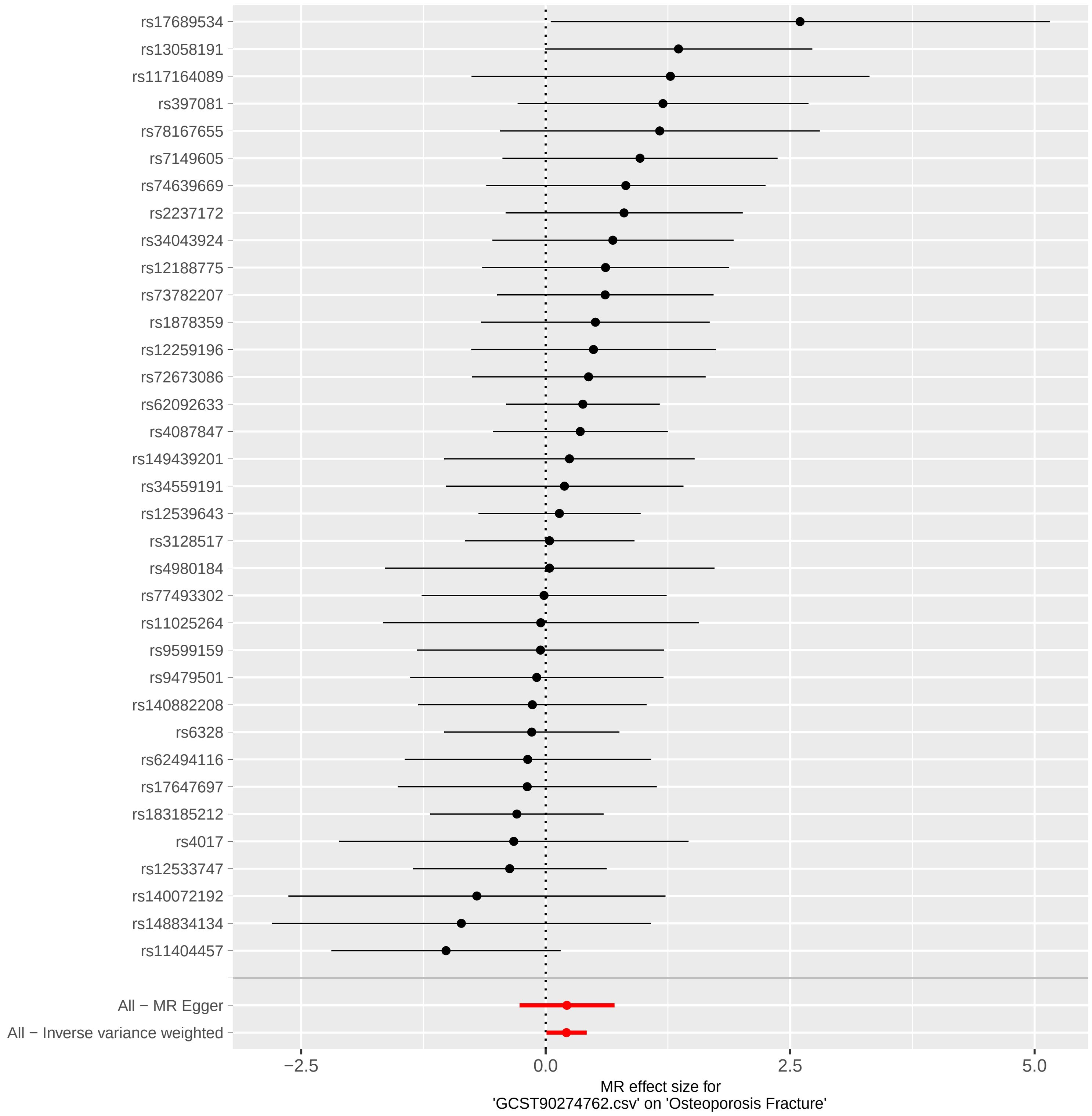
rs17689534
rs13058191
rs117164089
rs397081
rs78167655
rs7149605
rs74639669
rs2237172
rs34043924
rs12188775
rs73782207
rs1878359
rs12259196
rs72673086
rs62092633
rs4087847
rs149439201
rs34559191
rs12539643
rs3128517
rs4980184
rs77493302
rs11025264
rs9599159
rs9479501
rs140882208
rs6328
rs62494116
rs17647697
rs183185212
rs4017
rs12533747
rs140072192
rs148834134
rs11404457
All − MR Egger
All − Inverse variance weighted
−2.5
0.0
2.5
5.0
MR effect size for
'GCST90274762.csv' on 'Osteoporosis Fracture'

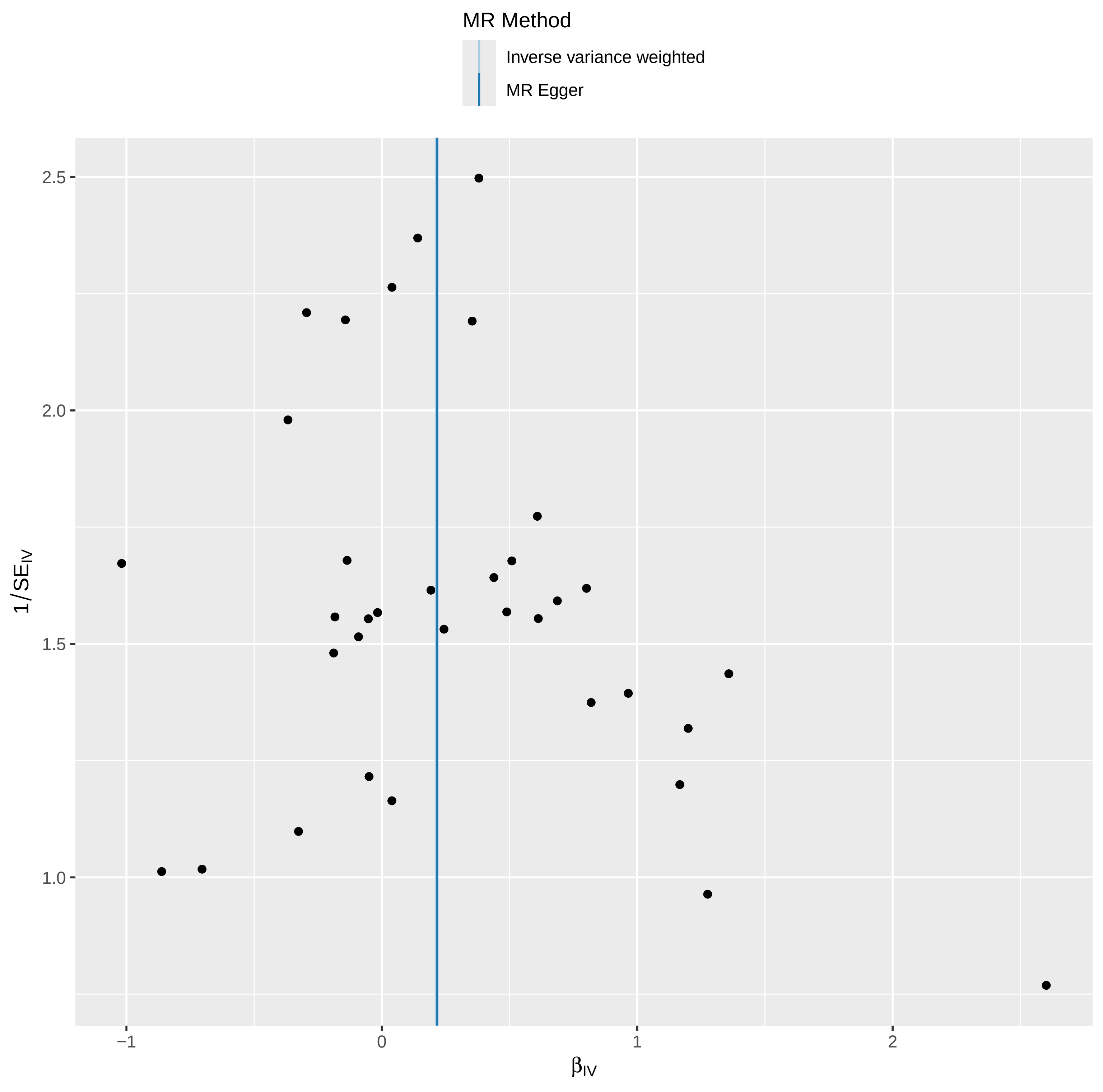
MR Method
Inverse variance weighted
MR Egger
2.5
2.0
1.5
1.0
1/SE_IV
−1
0
1
2
β_IV

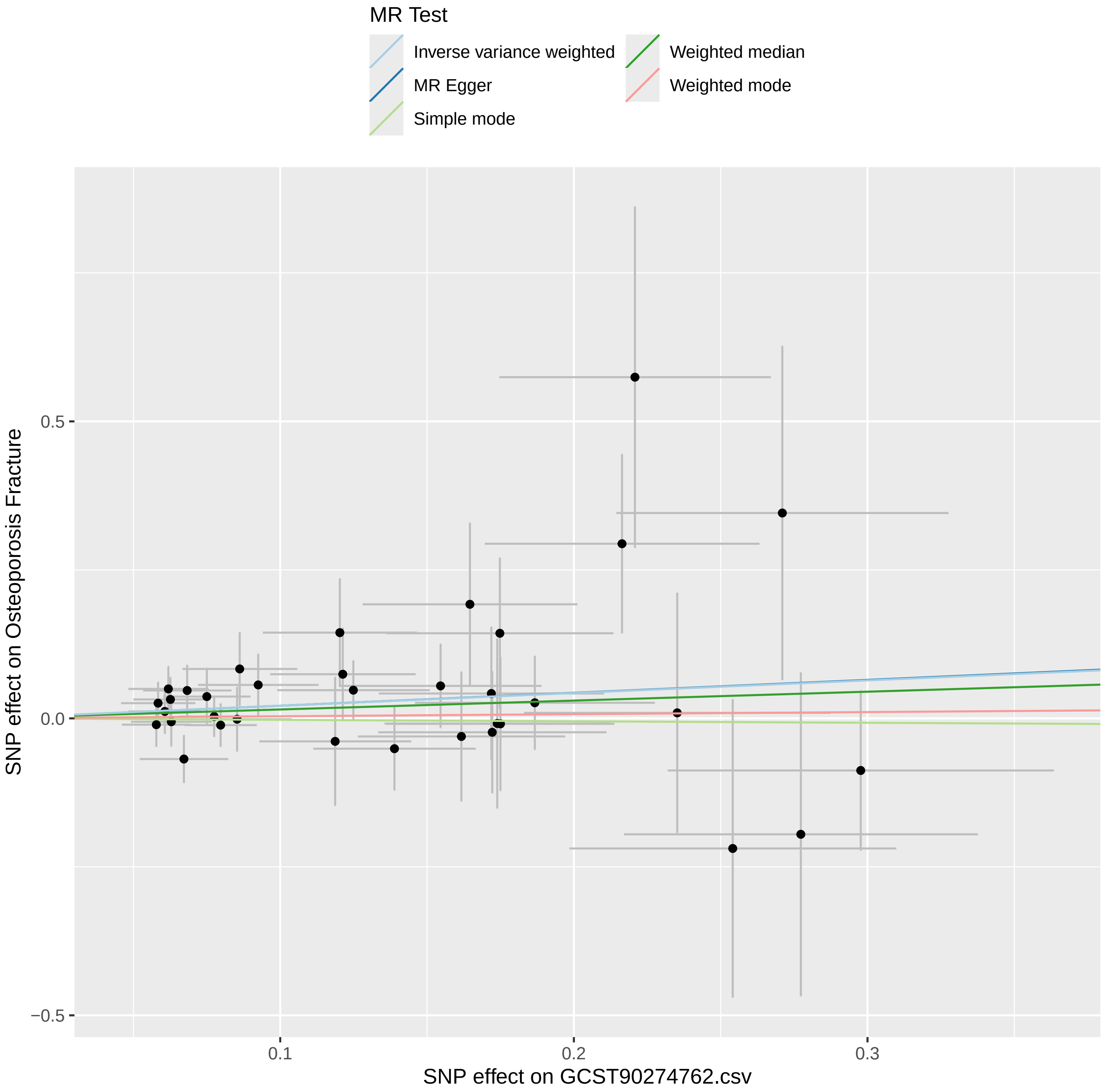

MR Test
Inverse variance weighted
MR Egger
Simple mode
Weighted median
Weighted mode
0.5
0.0
−0.5
0.1
0.2
0.3
SNP effect on Osteoporosis Fracture
SNP effect on GCST90274762.csv

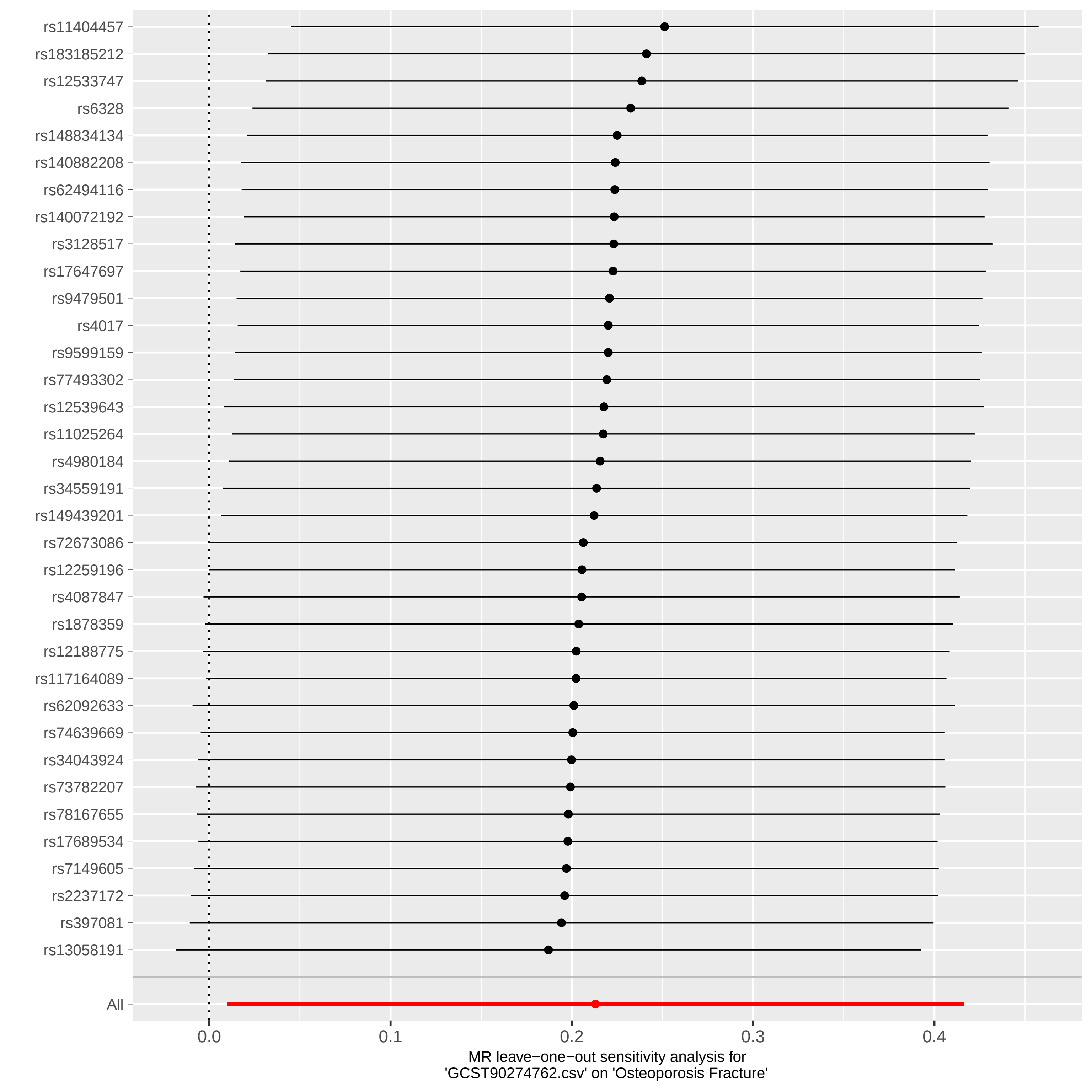
rs11404457
rs183185212
rs12533747
rs6328
rs148834134
rs140882208
rs62494116
rs140072192
rs3128517
rs17647697
rs9479501
rs4017
rs9599159
rs77493302
rs12539643
rs11025264
rs4980184
rs34559191
rs149439201
rs72673086
rs12259196
rs4087847
rs1878359
rs12188775
rs117164089
rs62092633
rs74639669
rs34043924
rs73782207
rs78167655
rs17689534
rs7149605
rs2237172
rs397081
rs13058191
All
0.0
0.1
0.2
0.3
0.4
MR leave−one−out sensitivity analysis for
'GCST90274762.csv' on 'Osteoporosis Fracture'

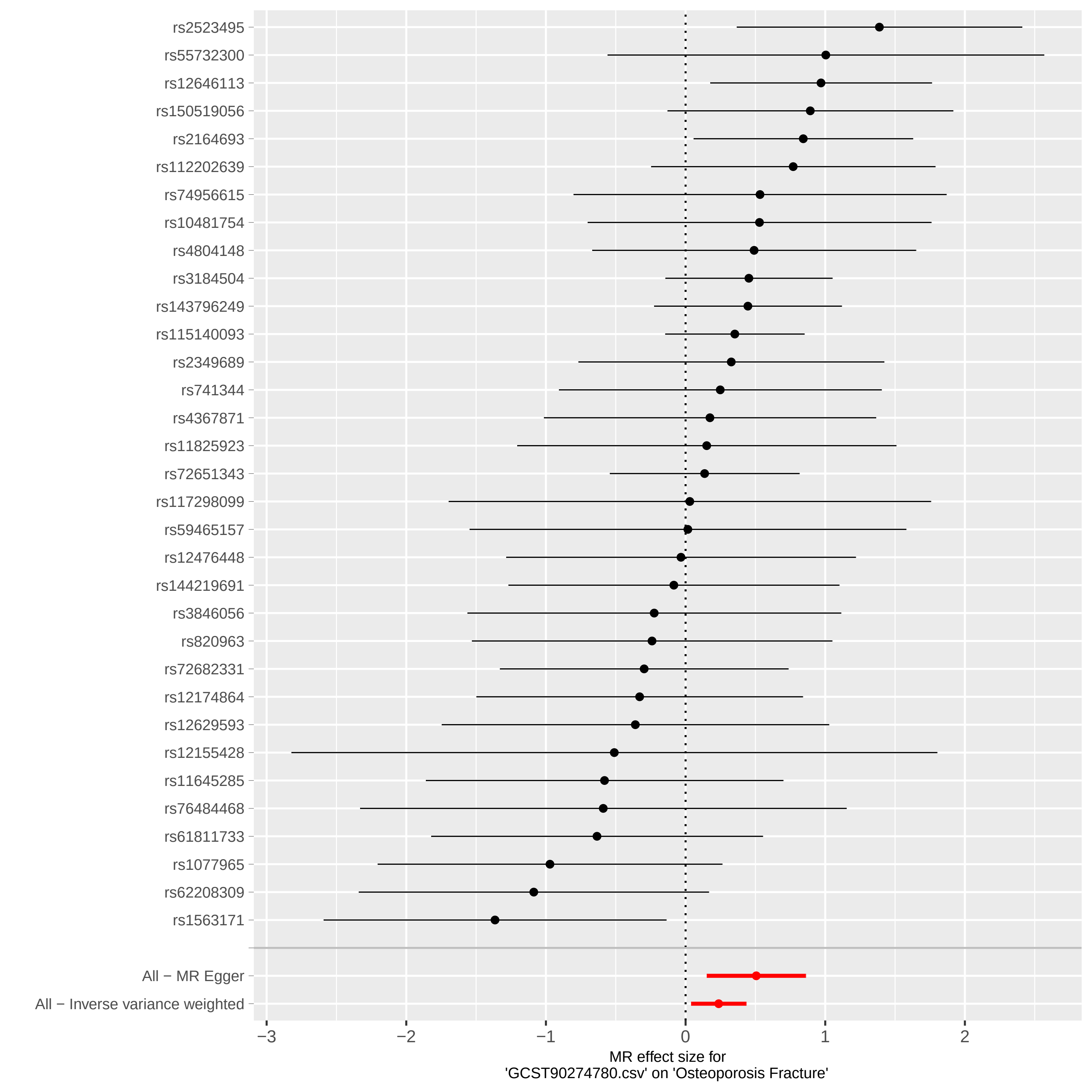
rs2523495
rs55732300
rs12646113
rs150519056
rs2164693
rs112202639
rs74956615
rs10481754
rs4804148
rs3184504
rs143796249
rs115140093
rs2349689
rs741344
rs4367871
rs11825923
rs72651343
rs117298099
rs59465157
rs12476448
rs144219691
rs3846056
rs820963
rs72682331
rs12174864
rs12629593
rs12155428
rs11645285
rs76484468
rs61811733
rs1077965
rs62208309
rs1563171
All − MR Egger
All − Inverse variance weighted
−3
−2
−1
0
1
2
MR effect size for
'GCST90274780.csv' on 'Osteoporosis Fracture'

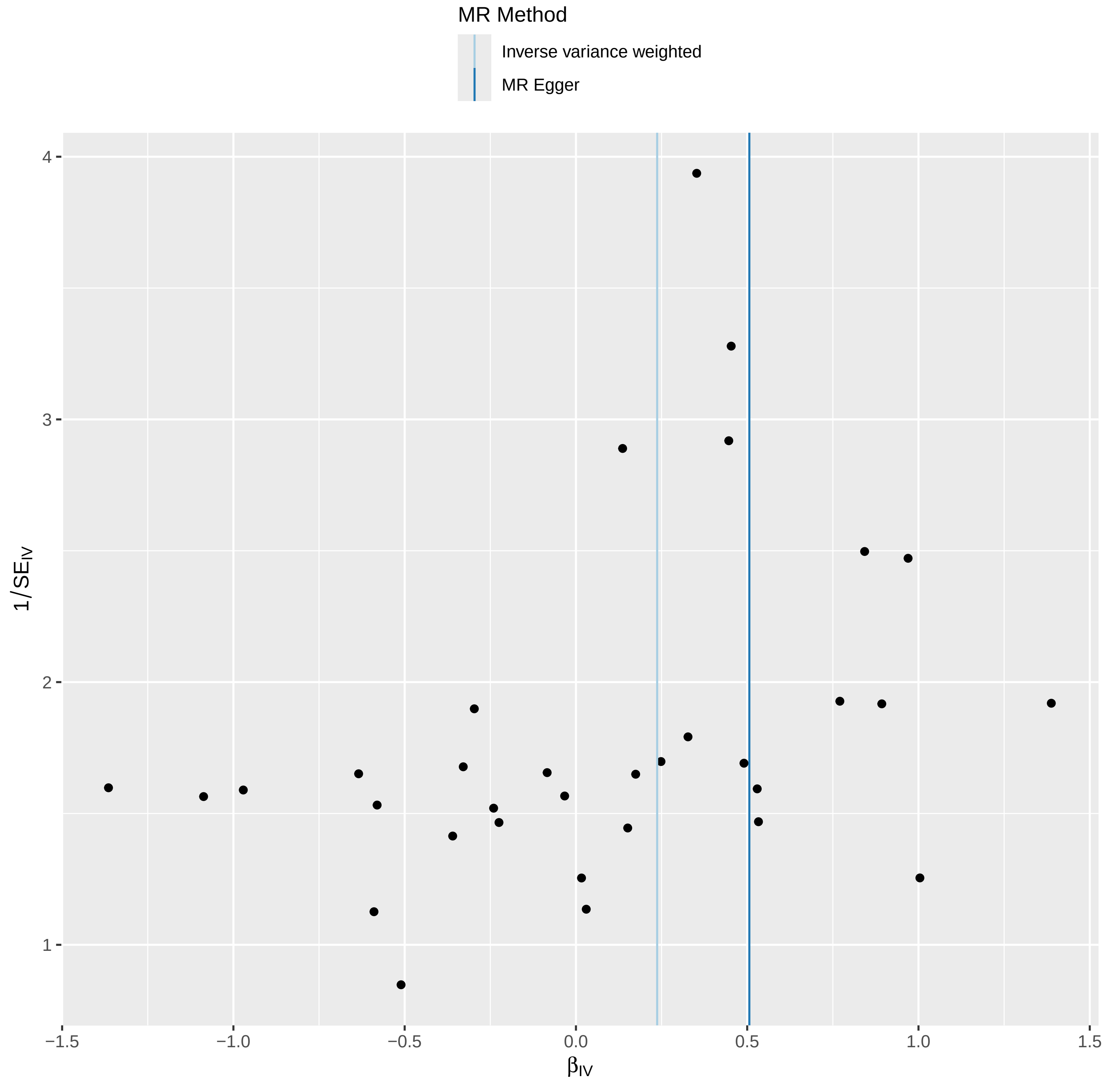

MR Method
Inverse variance weighted
MR Egger
1/SE_IV
β_IV
4
3
2
1
−1.5
−1.0
−0.5
0.0
0.5
1.0
1.5

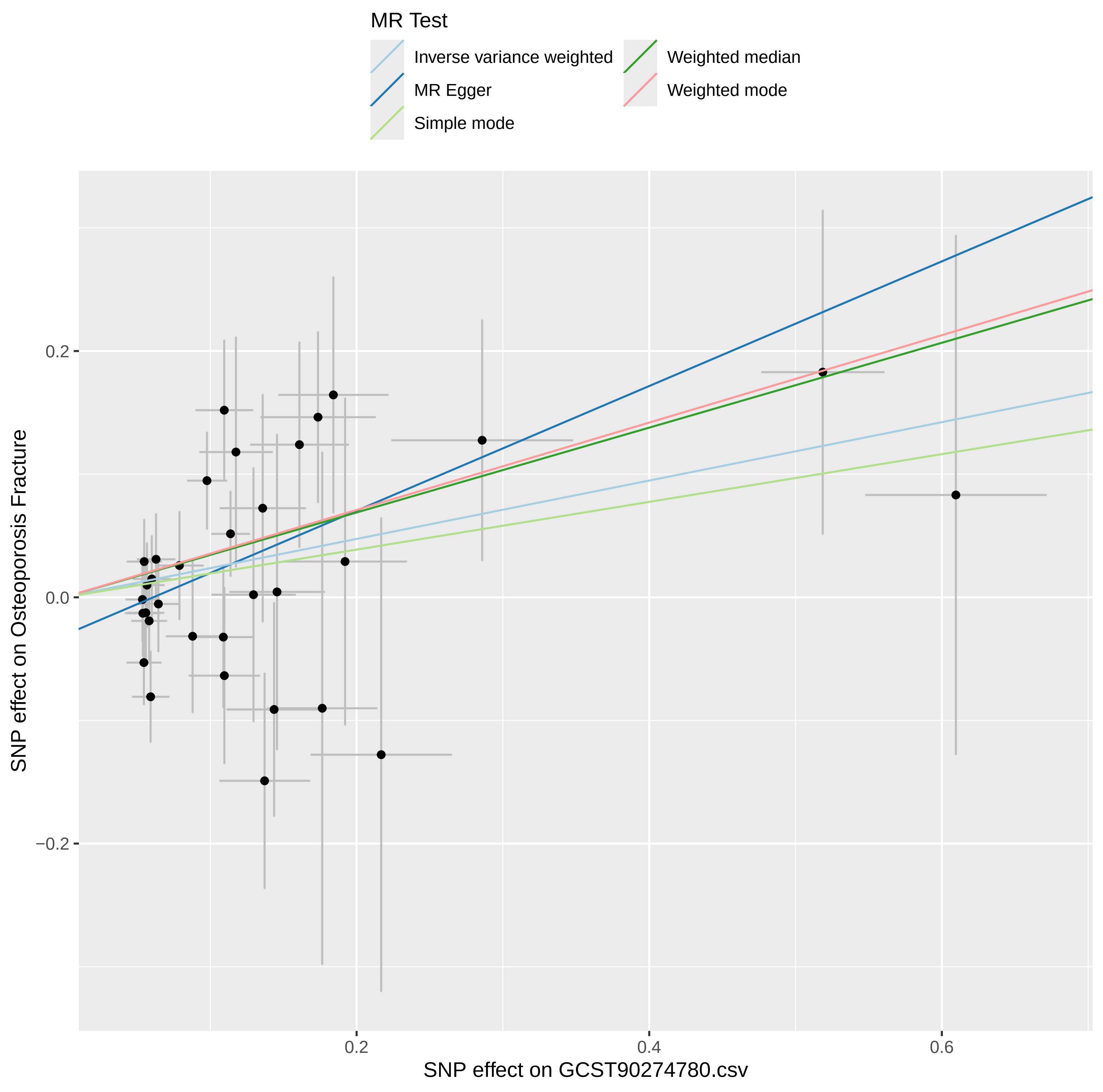
MR Test
Inverse variance weighted
MR Egger
Simple mode
Weighted median
Weighted mode
0.2
0.0
−0.2
SNP effect on Osteoporosis Fracture
0.2
0.4
0.6
SNP effect on GCST90274780.csv

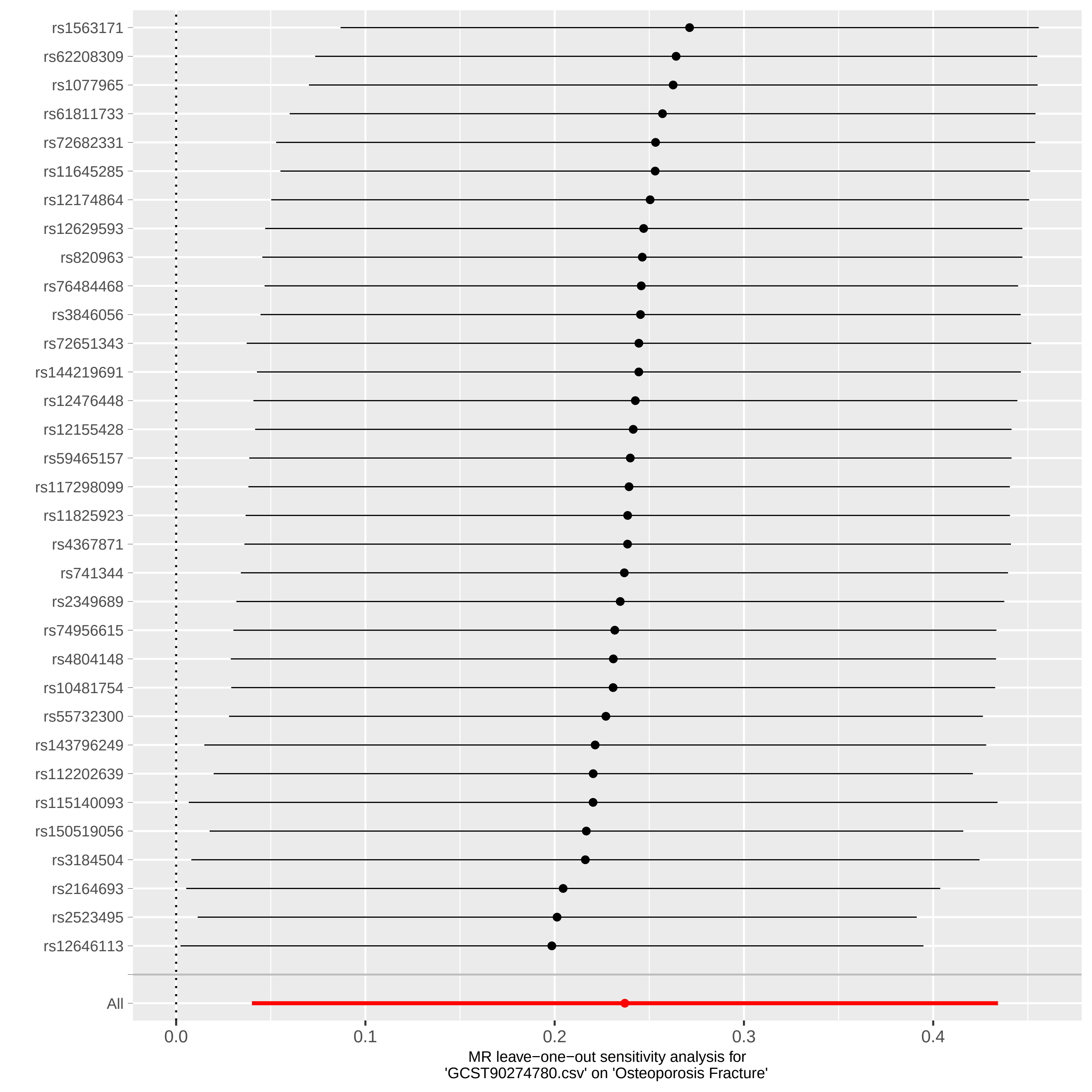

rs1563171
rs62208309
rs1077965
rs61811733
rs72682331
rs11645285
rs12174864
rs12629593
rs820963
rs76484468
rs3846056
rs72651343
rs144219691
rs12476448
rs12155428
rs59465157
rs117298099
rs11825923
rs4367871
rs741344
rs2349689
rs74956615
rs4804148
rs10481754
rs55732300
rs143796249
rs112202639
rs115140093
rs150519056
rs3184504
rs2164693
rs2523495
rs12646113
All
0.0
0.1
0.2
0.3
0.4
MR leave−one−out sensitivity analysis for
'GCST90274780.csv' on 'Osteoporosis Fracture'

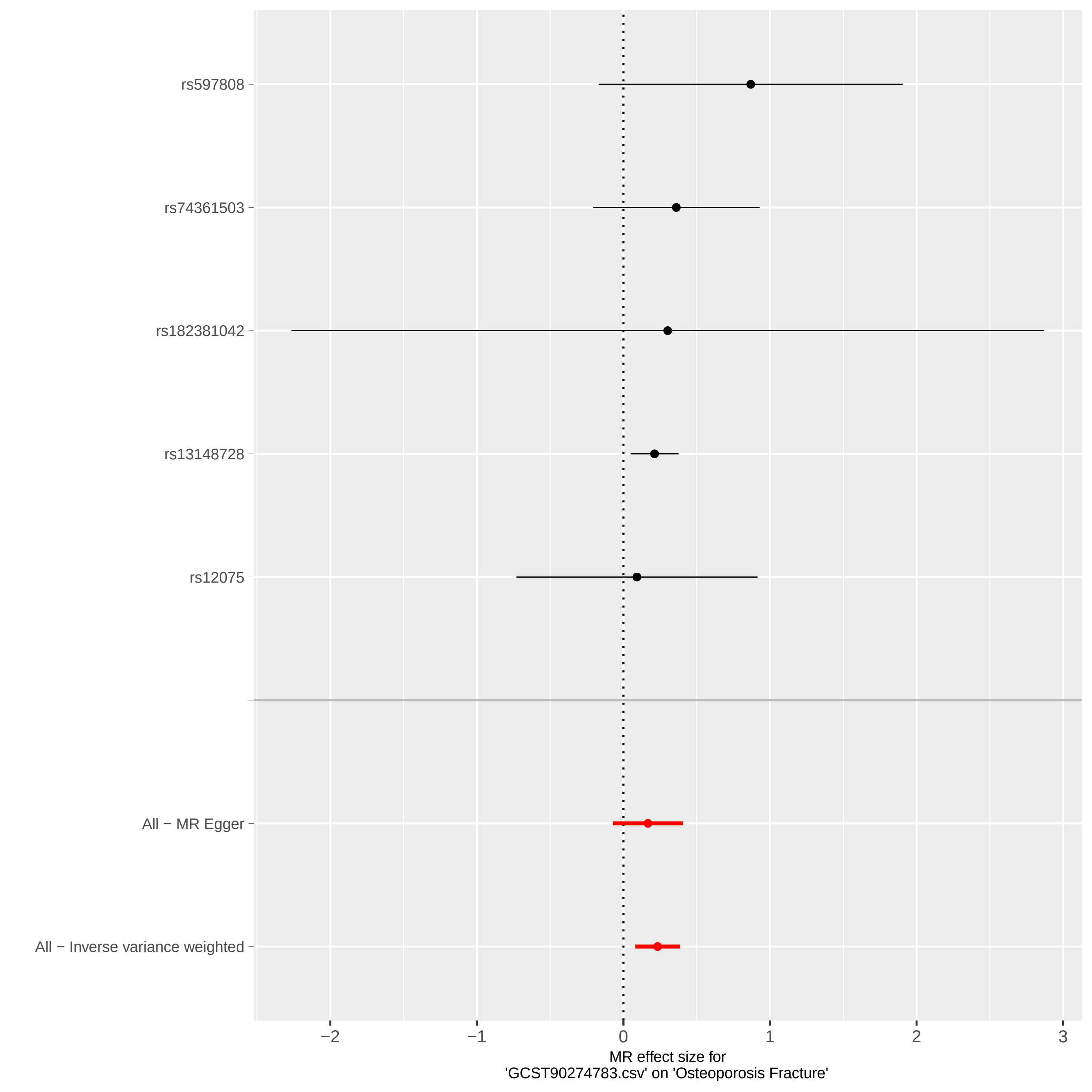
rs597808
rs74361503
rs182381042
rs13148728
rs12075
All − MR Egger
All − Inverse variance weighted
−2
−1
0
1
2
3
MR effect size for
'GCST90274783.csv' on 'Osteoporosis Fracture'

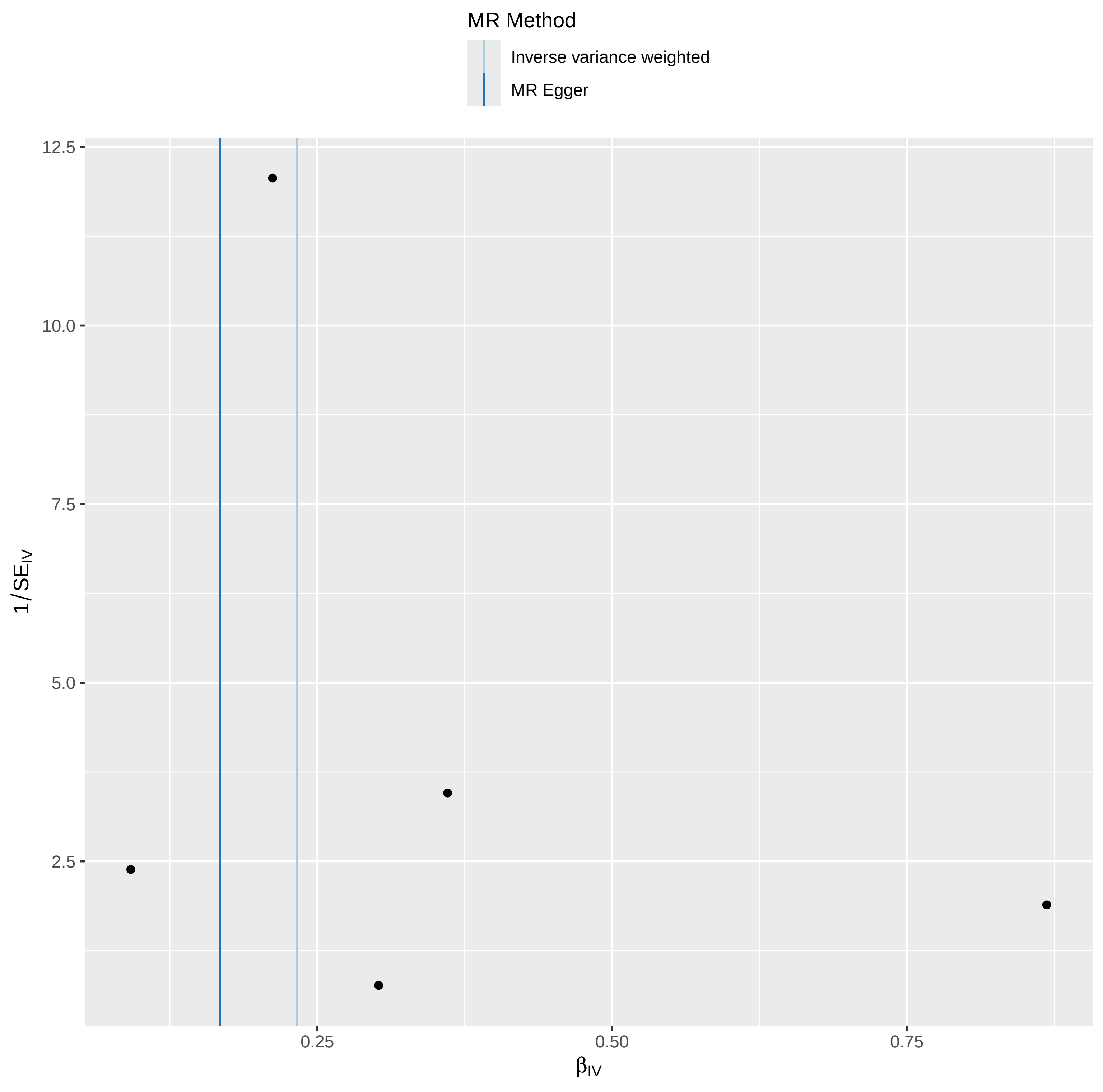

MR Method
Inverse variance weighted
MR Egger
12.5
10.0
7.5
5.0
2.5
1/SE$_{IV}$
0.25
0.50
0.75
β$_{IV}$

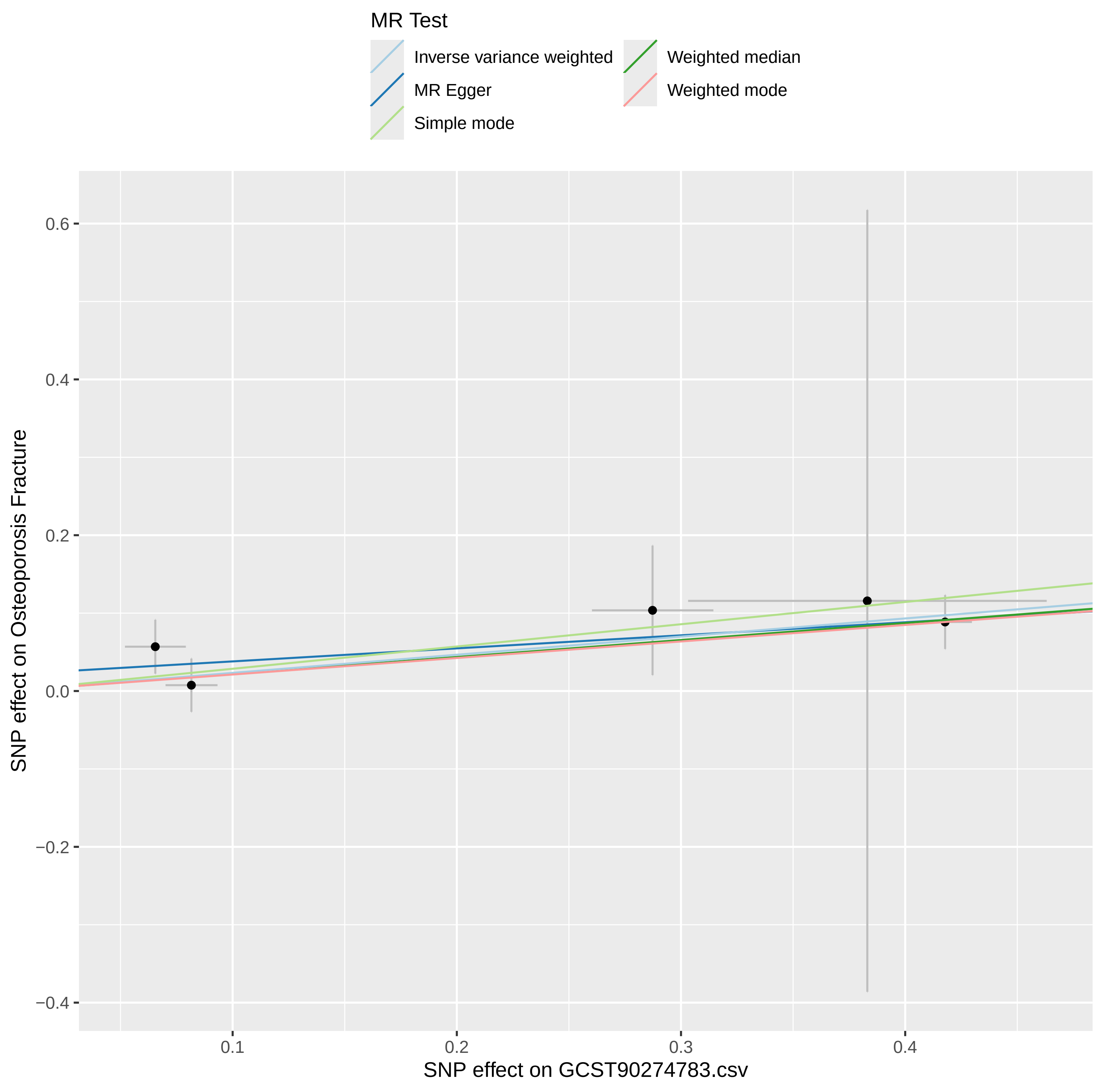

MR Test
Inverse variance weighted
MR Egger
Simple mode
Weighted median
Weighted mode
0.6
0.4
0.2
0.0
−0.2
−0.4
0.1
0.2
0.3
0.4
SNP effect on GCST90274783.csv
SNP effect on Osteoporosis Fracture

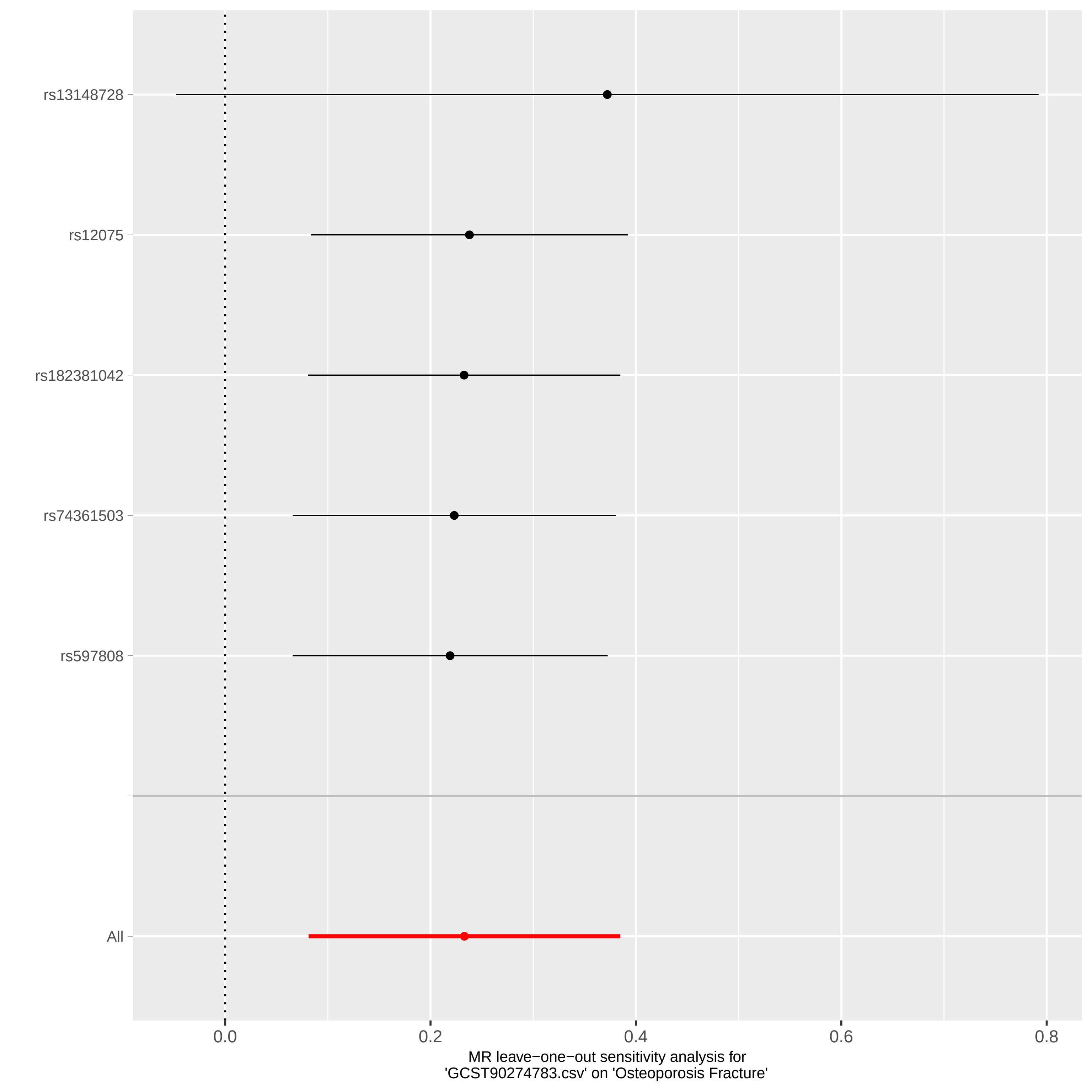
rs13148728
rs12075
rs182381042
rs74361503
rs597808
All
0.0
0.2
0.4
0.6
0.8
MR leave−one−out sensitivity analysis for
'GCST90274783.csv' on 'Osteoporosis Fracture'

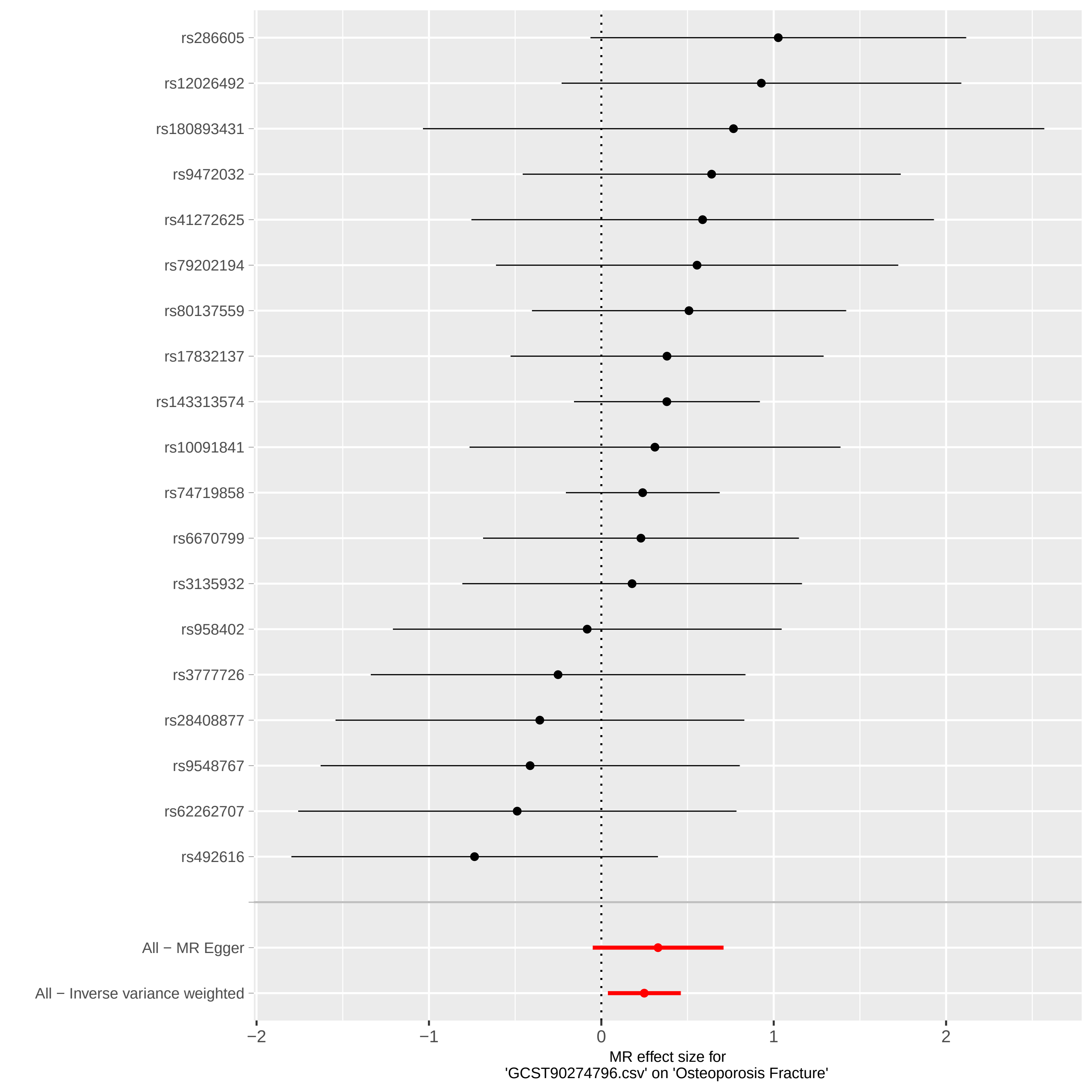
rs286605
rs12026492
rs180893431
rs9472032
rs41272625
rs79202194
rs80137559
rs17832137
rs143313574
rs10091841
rs74719858
rs6670799
rs3135932
rs958402
rs3777726
rs28408877
rs9548767
rs62262707
rs492616
All − MR Egger
All − Inverse variance weighted
−2
−1
0
1
2
MR effect size for
'GCST90274796.csv' on 'Osteoporosis Fracture'

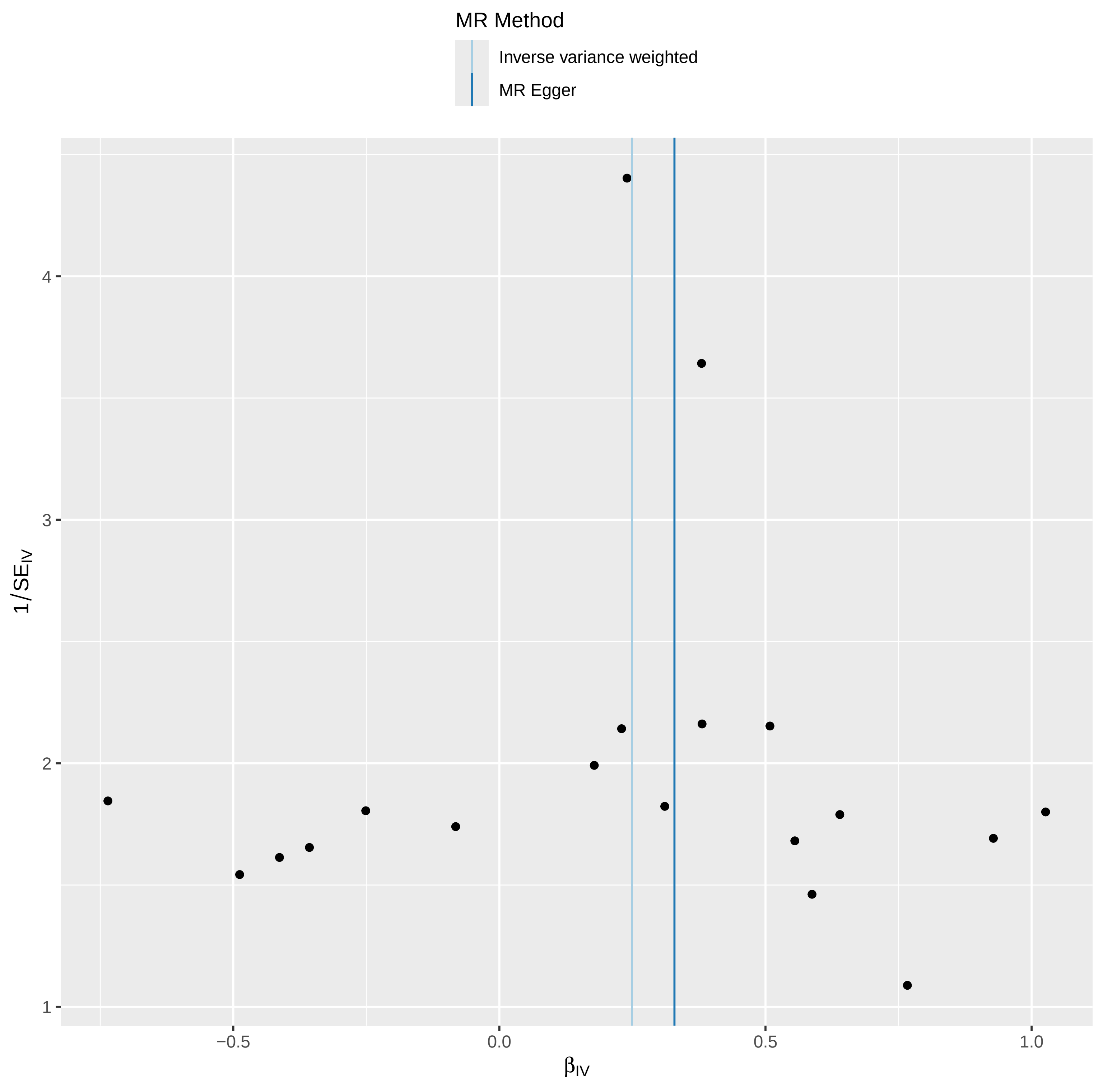

MR Method
Inverse variance weighted
MR Egger
4
3
2
1
1/SE_IV
−0.5
0.0
0.5
1.0
β_IV

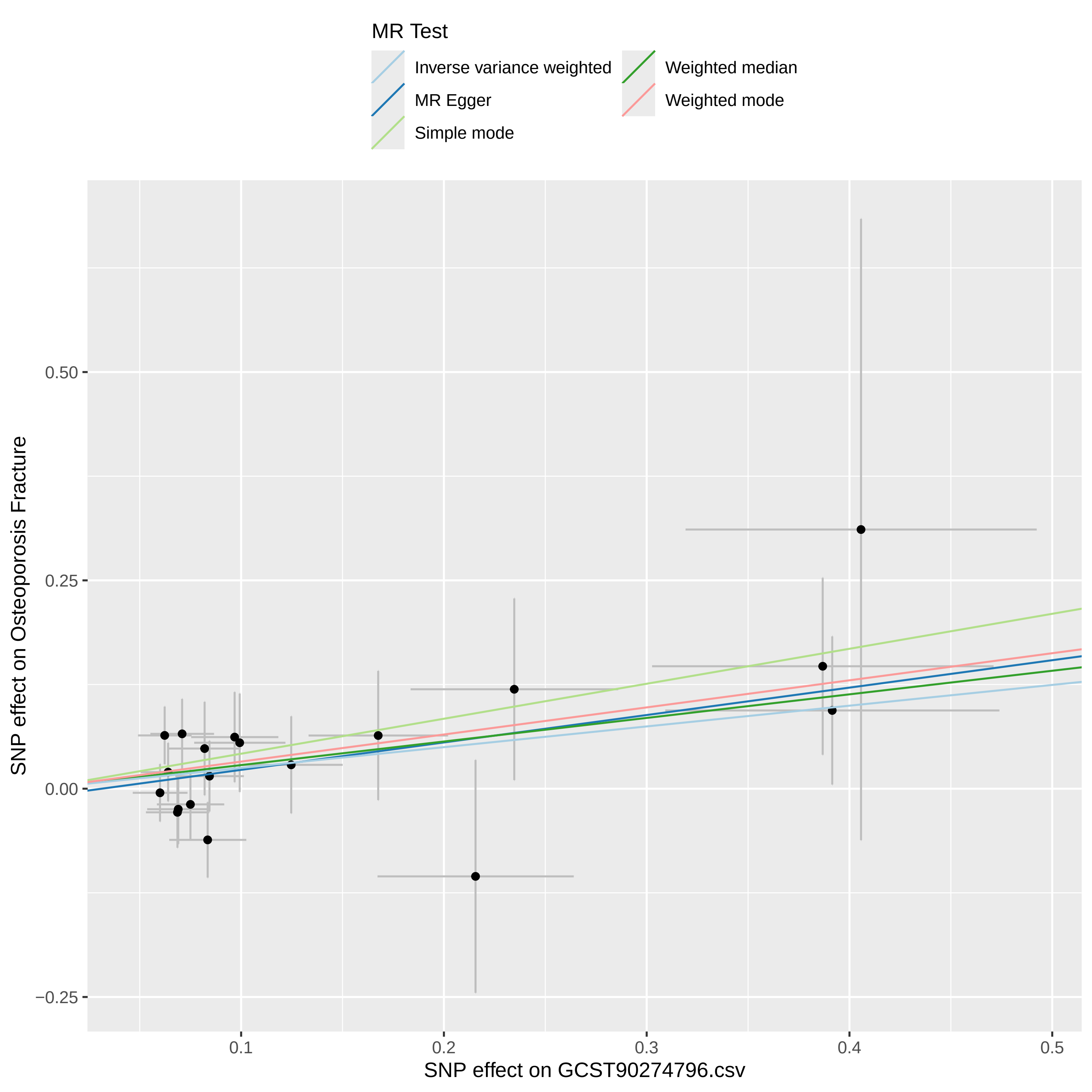

MR Test
Inverse variance weighted
MR Egger
Simple mode
Weighted median
Weighted mode
SNP effect on Osteoporosis Fracture
0.50
0.25
0.00
−0.25
0.1
0.2
0.3
0.4
0.5
SNP effect on GCST90274796.csv

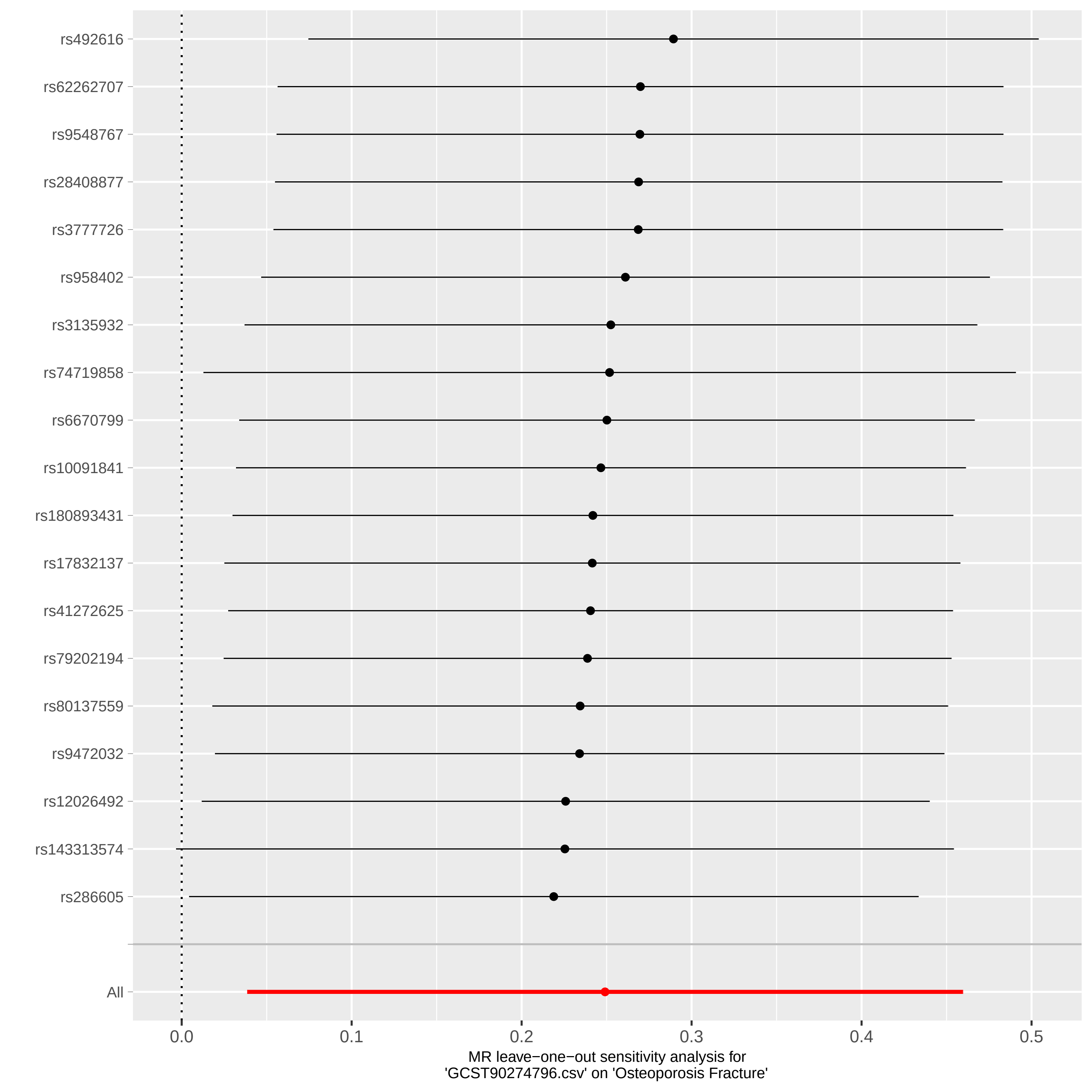
rs492616
rs62262707
rs9548767
rs28408877
rs3777726
rs958402
rs3135932
rs74719858
rs6670799
rs10091841
rs180893431
rs17832137
rs41272625
rs79202194
rs80137559
rs9472032
rs12026492
rs143313574
rs286605
All
0.0
0.1
0.2
0.3
0.4
0.5
MR leave−one−out sensitivity analysis for
'GCST90274796.csv' on 'Osteoporosis Fracture'

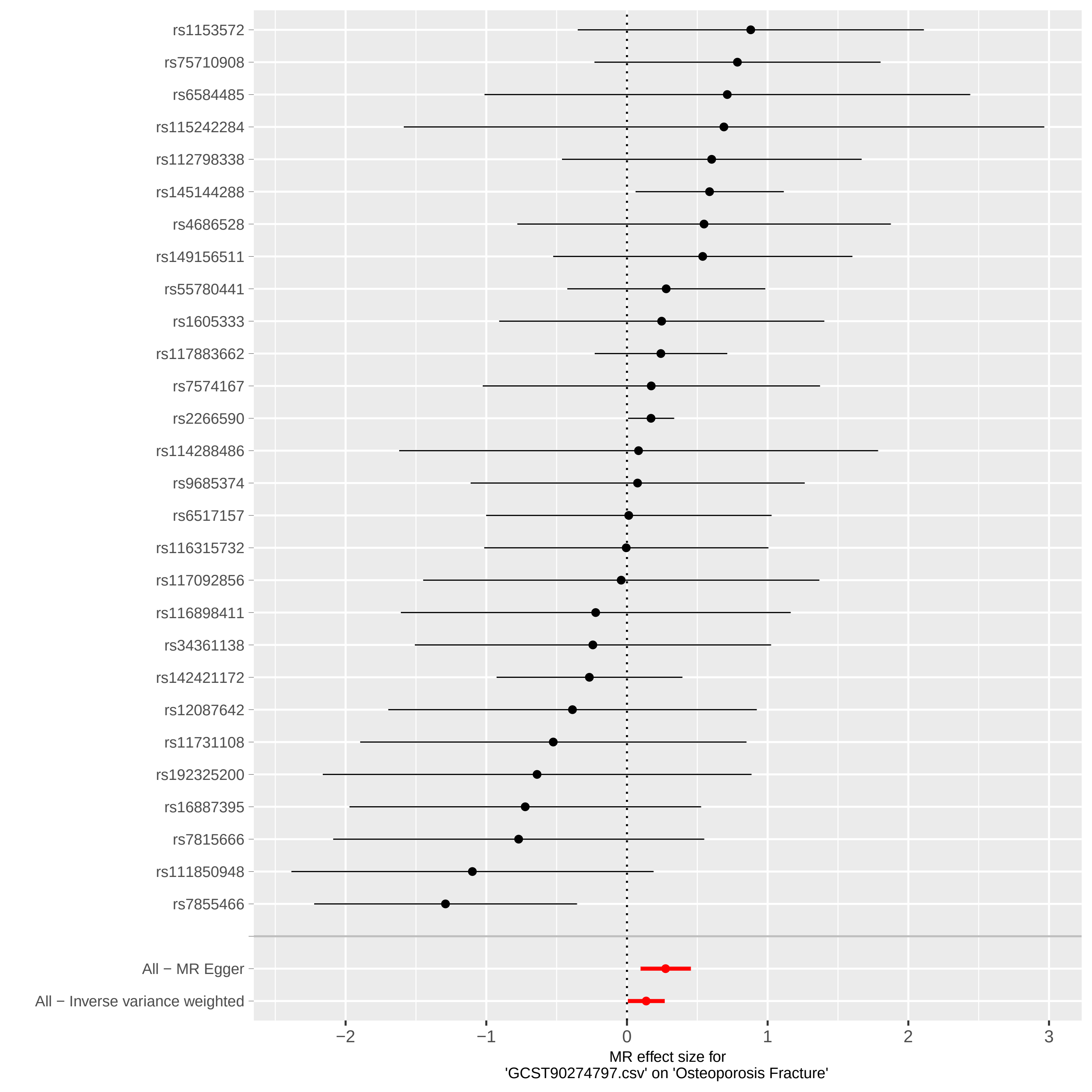
rs1153572
rs75710908
rs6584485
rs115242284
rs112798338
rs145144288
rs4686528
rs149156511
rs55780441
rs1605333
rs117883662
rs7574167
rs2266590
rs114288486
rs9685374
rs6517157
rs116315732
rs117092856
rs116898411
rs34361138
rs142421172
rs12087642
rs11731108
rs192325200
rs16887395
rs7815666
rs111850948
rs7855466
All − MR Egger
All − Inverse variance weighted
−2
−1
0
1
2
3
MR effect size for
'GCST90274797.csv' on 'Osteoporosis Fracture'

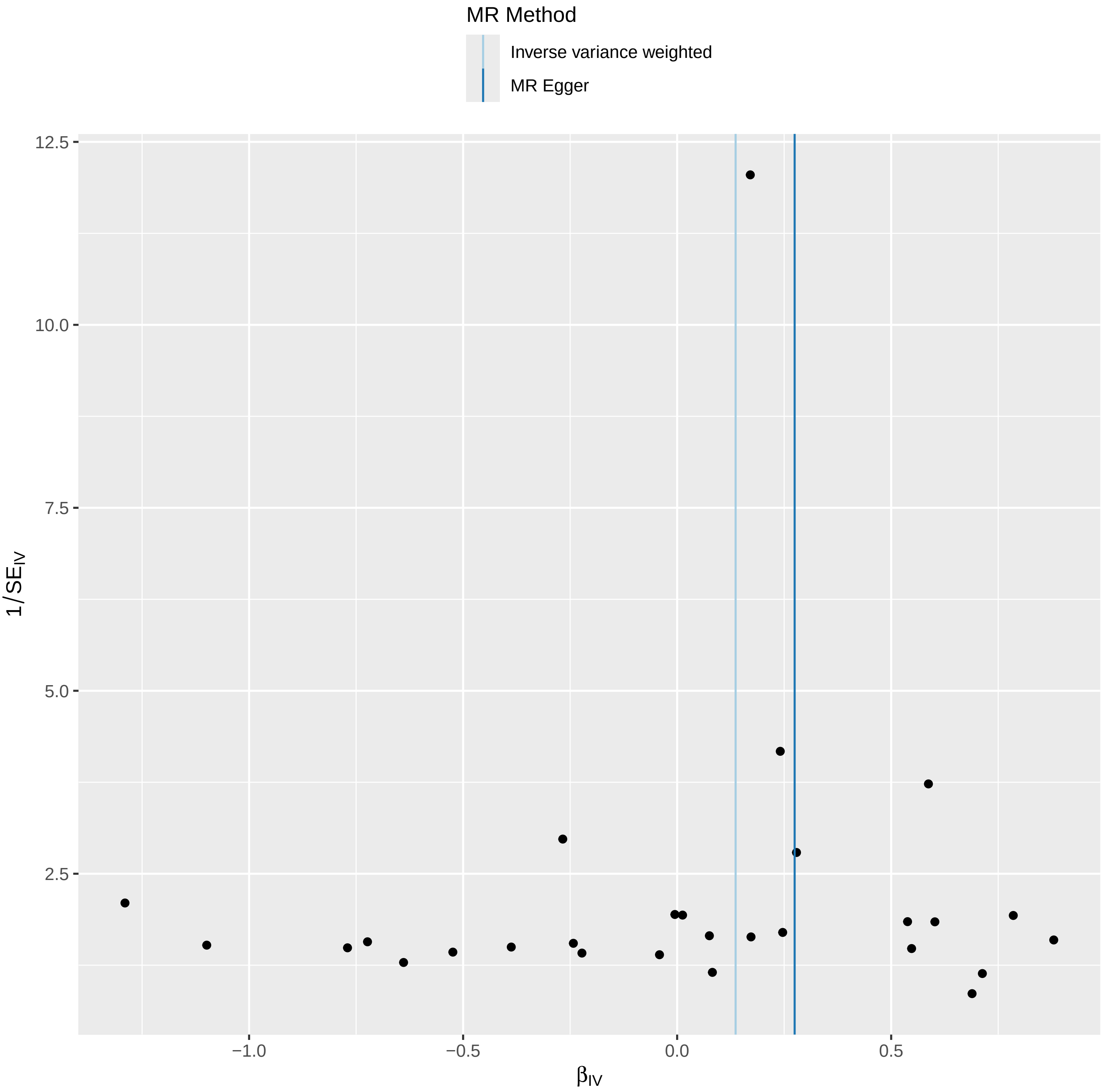

MR Method
Inverse variance weighted
MR Egger
12.5
10.0
7.5
5.0
2.5
1/SE_IV
−1.0
−0.5
0.0
0.5
β_IV

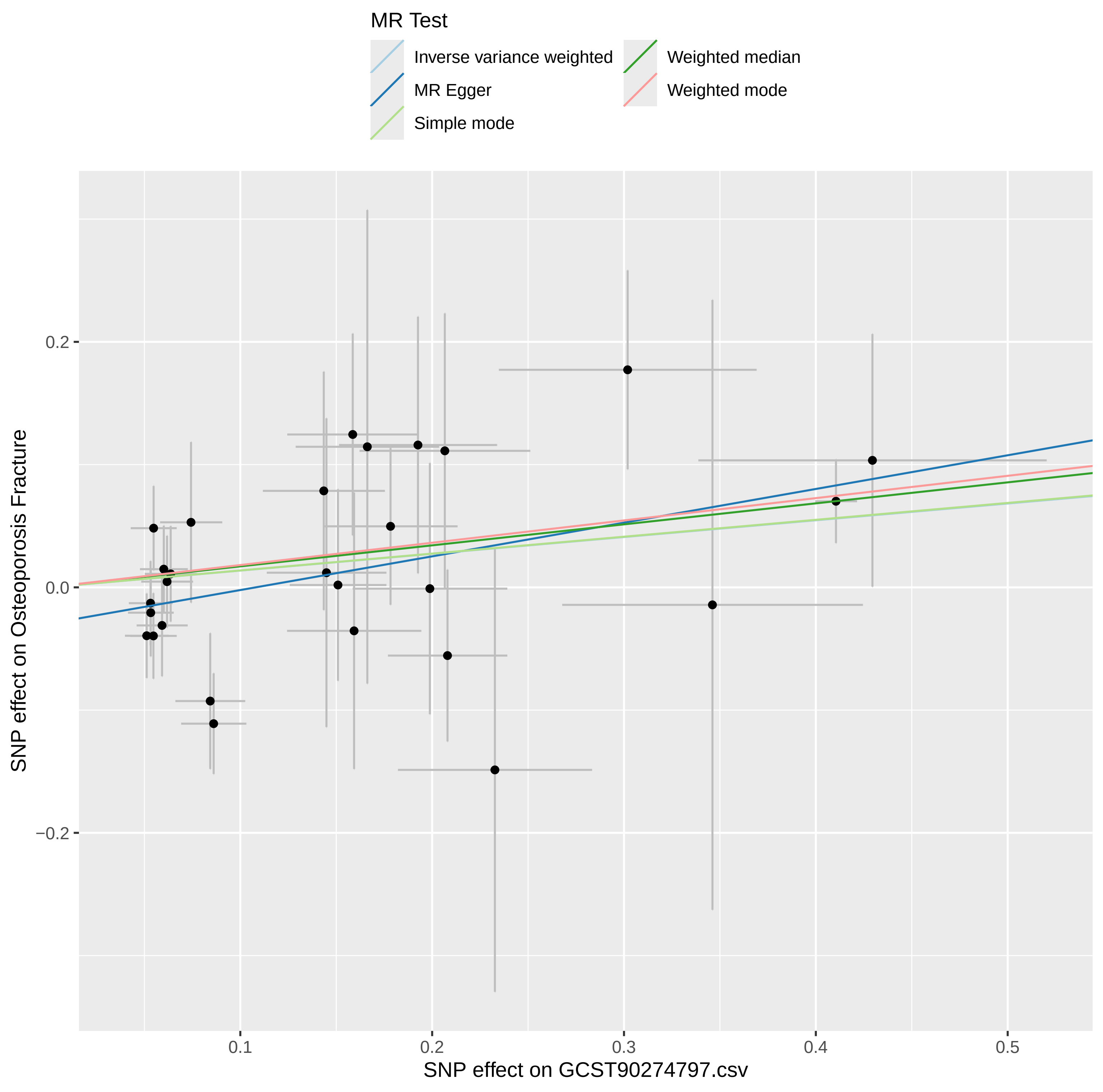
MR Test
Inverse variance weighted
MR Egger
Simple mode
Weighted median
Weighted mode
0.2
0.0
−0.2
0.1
0.2
0.3
0.4
0.5
SNP effect on Osteoporosis Fracture
SNP effect on GCST90274797.csv

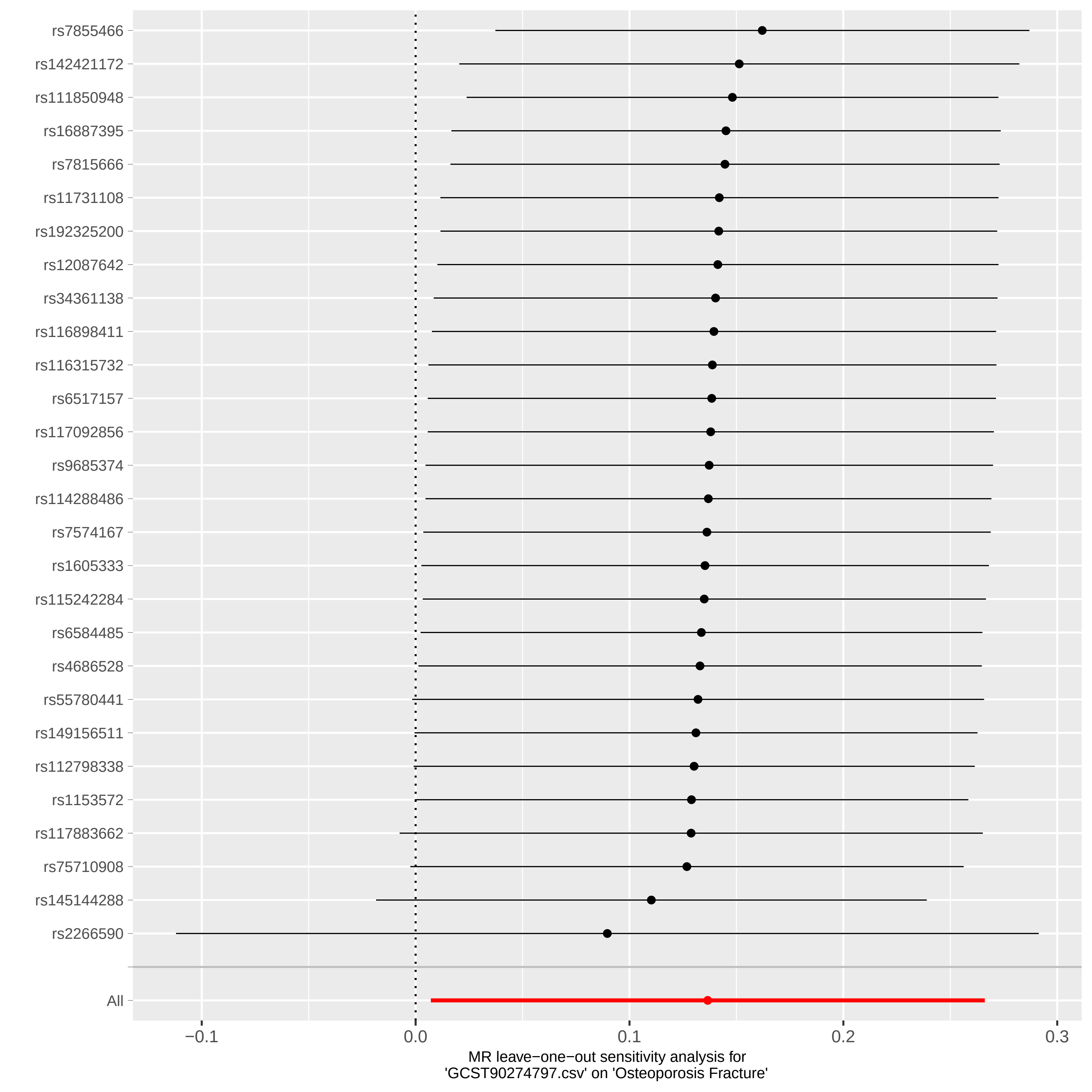

rs7855466
rs142421172
rs111850948
rs16887395
rs7815666
rs11731108
rs192325200
rs12087642
rs34361138
rs116898411
rs116315732
rs6517157
rs117092856
rs9685374
rs114288486
rs7574167
rs1605333
rs115242284
rs6584485
rs4686528
rs55780441
rs149156511
rs112798338
rs1153572
rs117883662
rs75710908
rs145144288
rs2266590
All
−0.1
0.0
0.1
0.2
0.3
MR leave−one−out sensitivity analysis for
'GCST90274797.csv' on 'Osteoporosis Fracture'

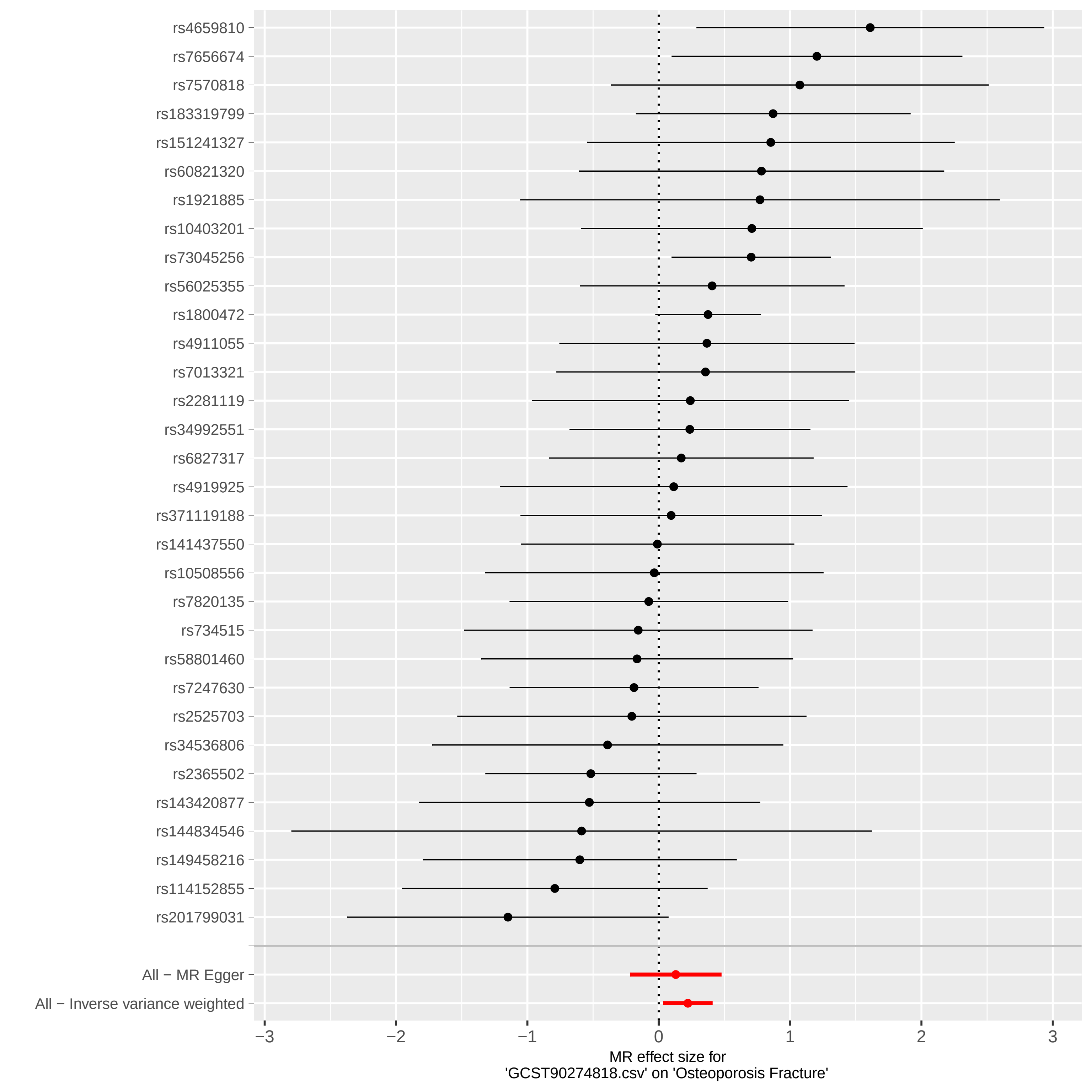
rs4659810
rs7656674
rs7570818
rs183319799
rs151241327
rs60821320
rs1921885
rs10403201
rs73045256
rs56025355
rs1800472
rs4911055
rs7013321
rs2281119
rs34992551
rs6827317
rs4919925
rs371119188
rs141437550
rs10508556
rs7820135
rs734515
rs58801460
rs7247630
rs2525703
rs34536806
rs2365502
rs143420877
rs144834546
rs149458216
rs114152855
rs201799031
All − MR Egger
All − Inverse variance weighted
−3
−2
−1
0
1
2
3
MR effect size for
'GCST90274818.csv' on 'Osteoporosis Fracture'

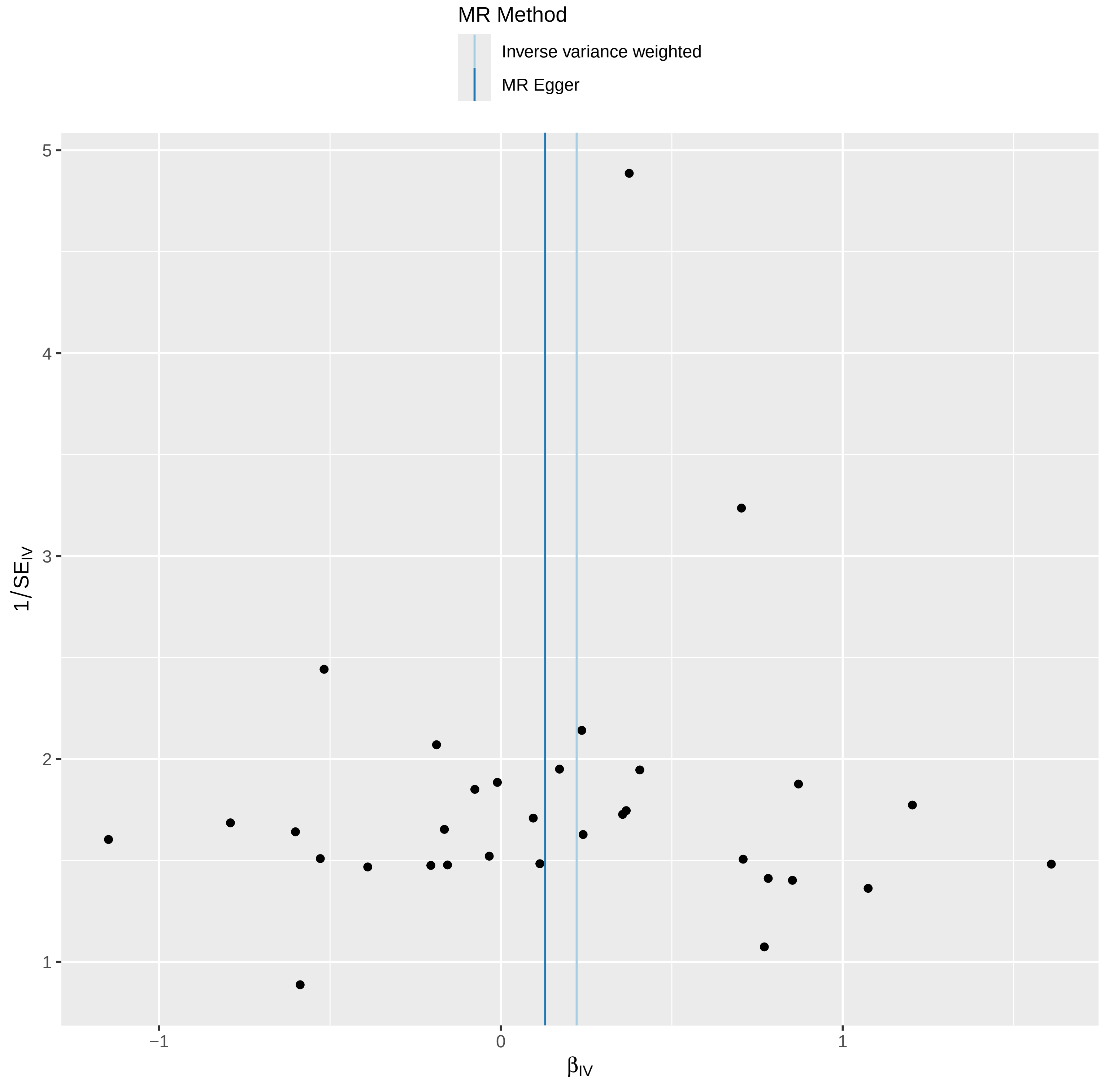
MR Method
Inverse variance weighted
MR Egger
1/SE$_{IV}$
5
4
3
2
1
−1
0
1
β$_{IV}$

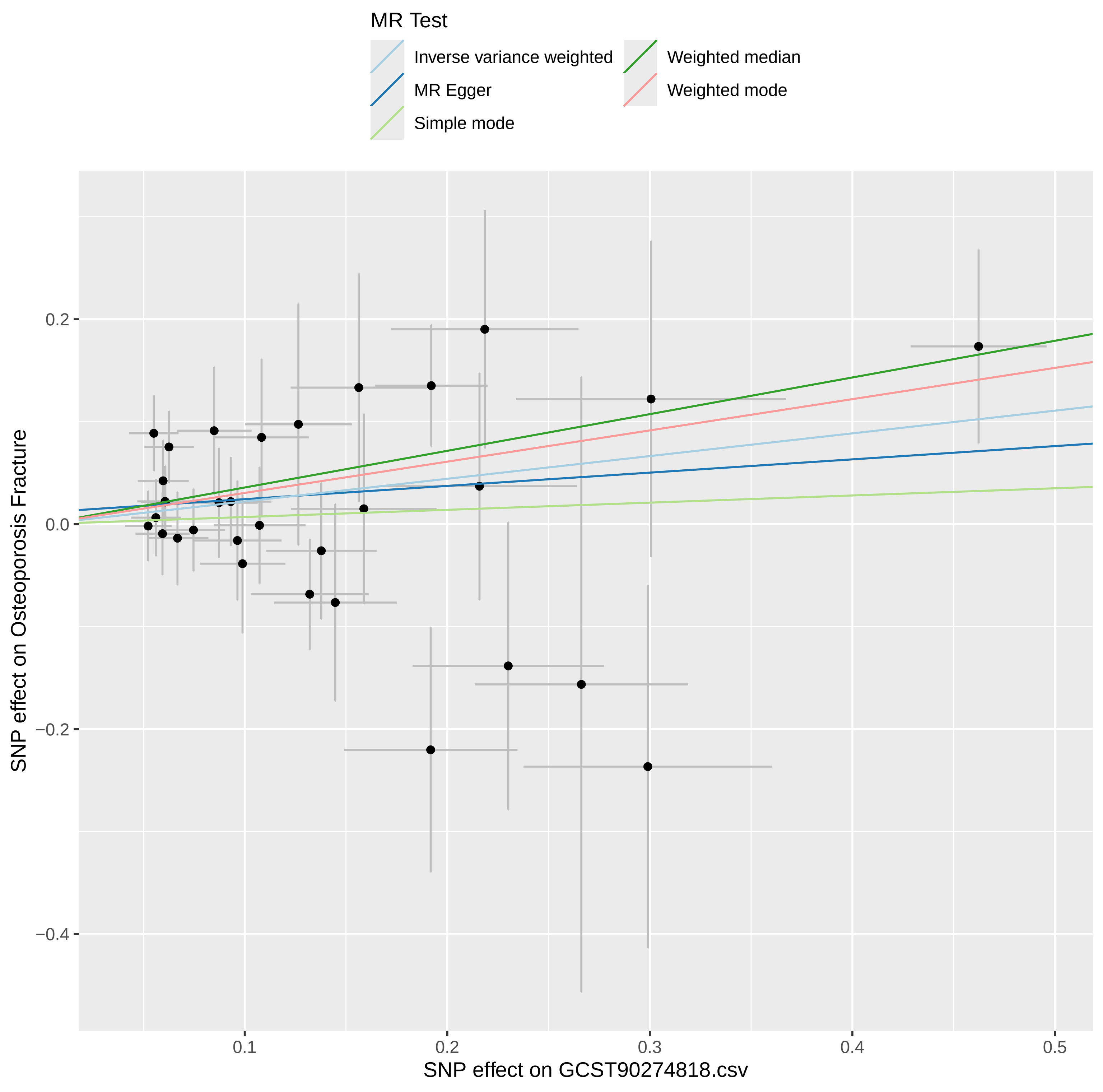
MR Test
Inverse variance weighted
MR Egger
Simple mode
Weighted median
Weighted mode
SNP effect on Osteoporosis Fracture
SNP effect on GCST90274818.csv
0.2
0.0
−0.2
−0.4
0.1
0.2
0.3
0.4
0.5

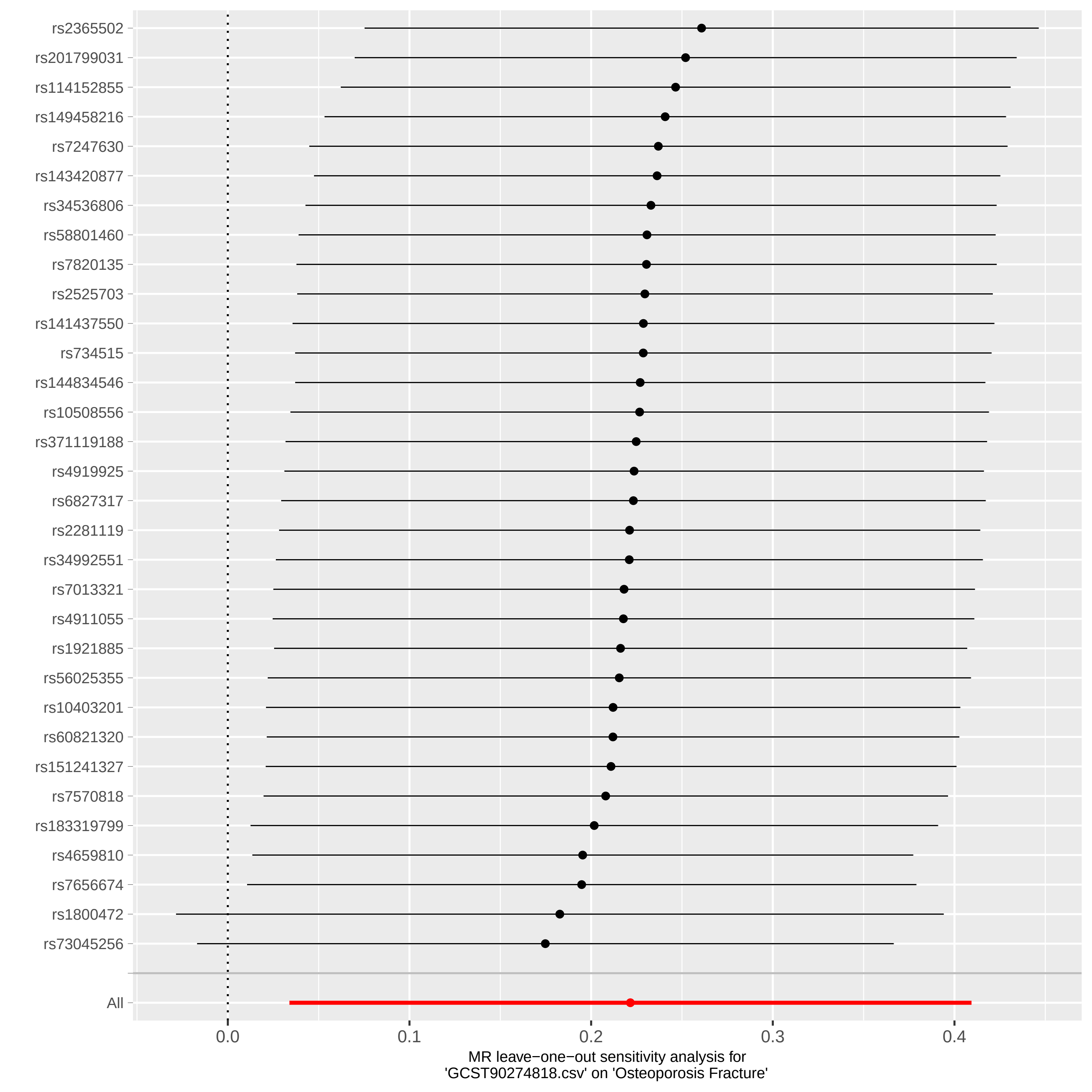

rs2365502
rs201799031
rs114152855
rs149458216
rs7247630
rs143420877
rs34536806
rs58801460
rs7820135
rs2525703
rs141437550
rs734515
rs144834546
rs10508556
rs371119188
rs4919925
rs6827317
rs2281119
rs34992551
rs7013321
rs4911055
rs1921885
rs56025355
rs10403201
rs60821320
rs151241327
rs7570818
rs183319799
rs4659810
rs7656674
rs1800472
rs73045256
All
0.0
0.1
0.2
0.3
0.4
MR leave−one−out sensitivity analysis for
'GCST90274818.csv' on 'Osteoporosis Fracture'

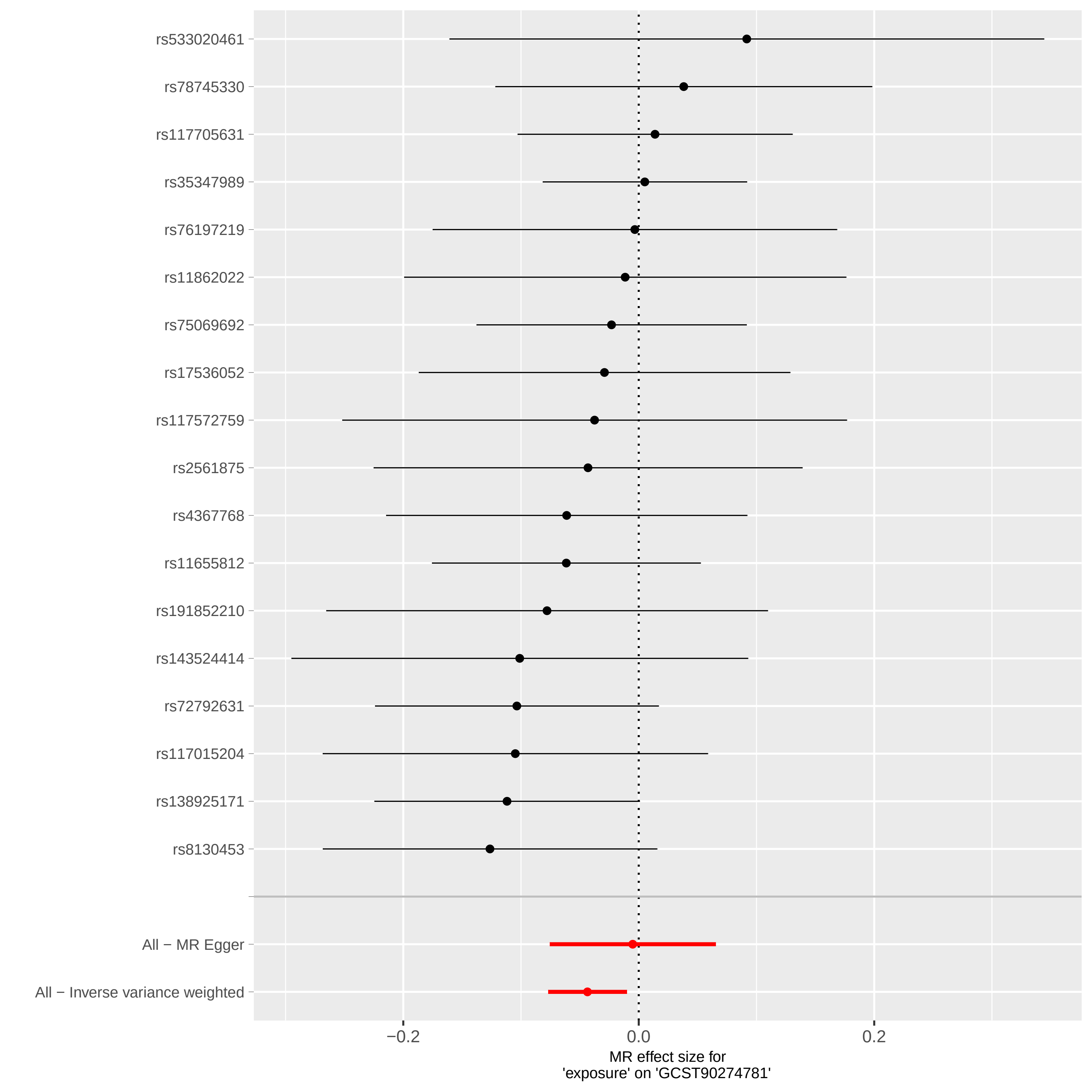
rs533020461
rs78745330
rs117705631
rs35347989
rs76197219
rs11862022
rs75069692
rs17536052
rs117572759
rs2561875
rs4367768
rs11655812
rs191852210
rs143524414
rs72792631
rs117015204
rs138925171
rs8130453
All − MR Egger
All − Inverse variance weighted
−0.2
0.0
0.2
MR effect size for
'exposure' on 'GCST90274781'

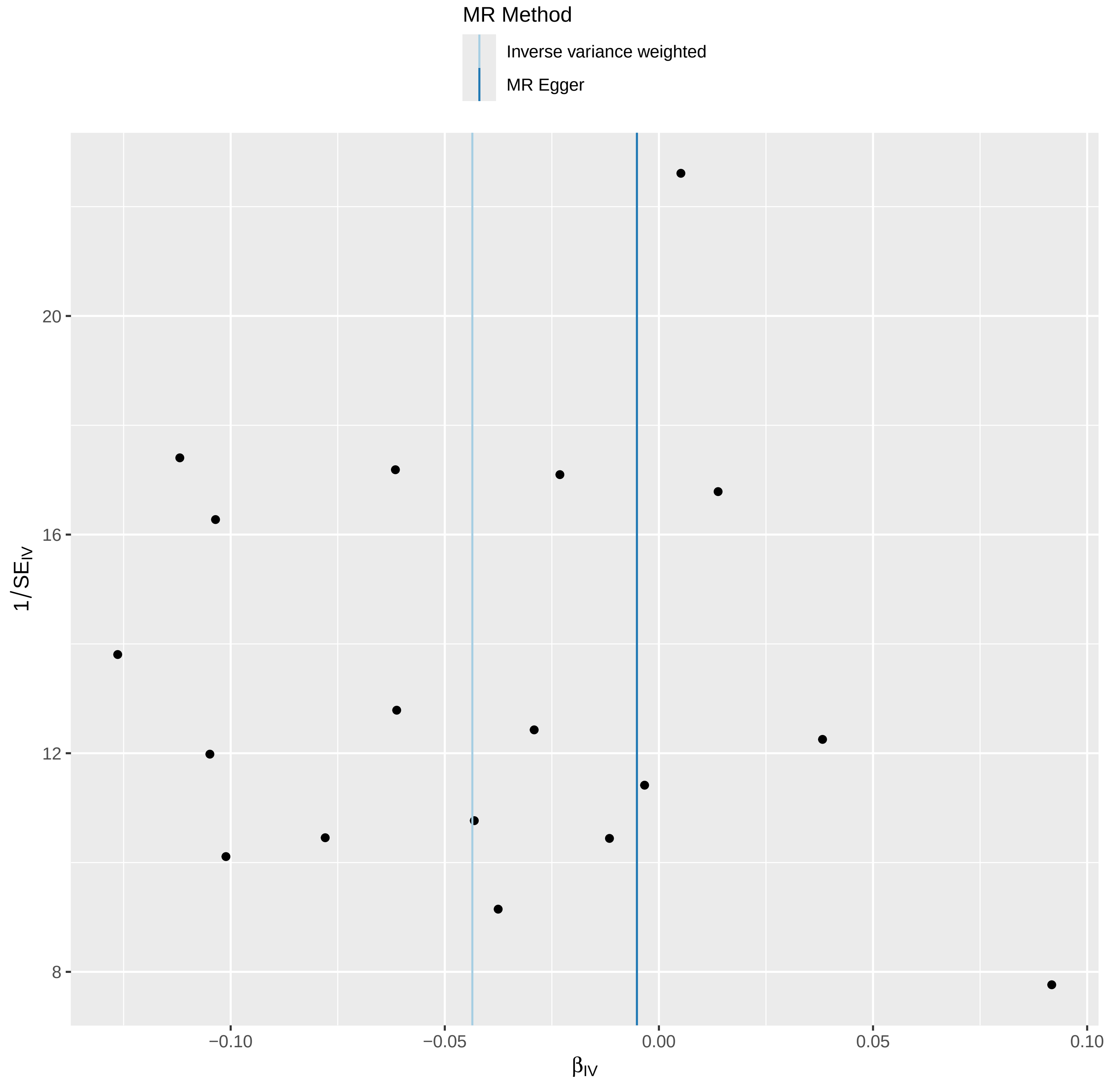

MR Method
Inverse variance weighted
MR Egger
20
16
12
8
$1/SE_{IV}$
−0.10
−0.05
0.00
0.05
0.10
β_{IV}

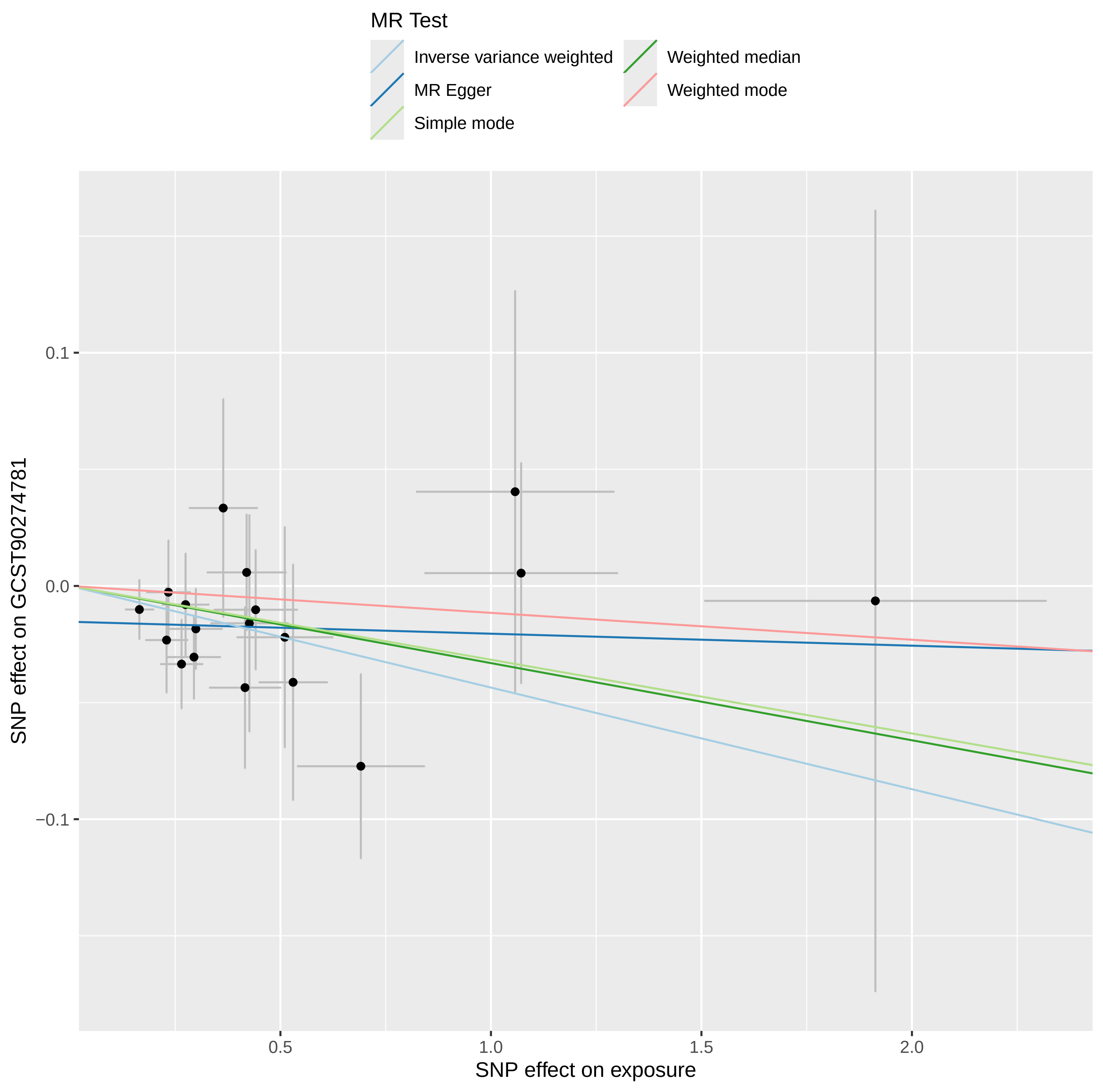

MR Test
Inverse variance weighted
MR Egger
Simple mode
Weighted median
Weighted mode
0.1
0.0
−0.1
0.5
1.0
1.5
2.0
SNP effect on exposure
SNP effect on GCST90274781

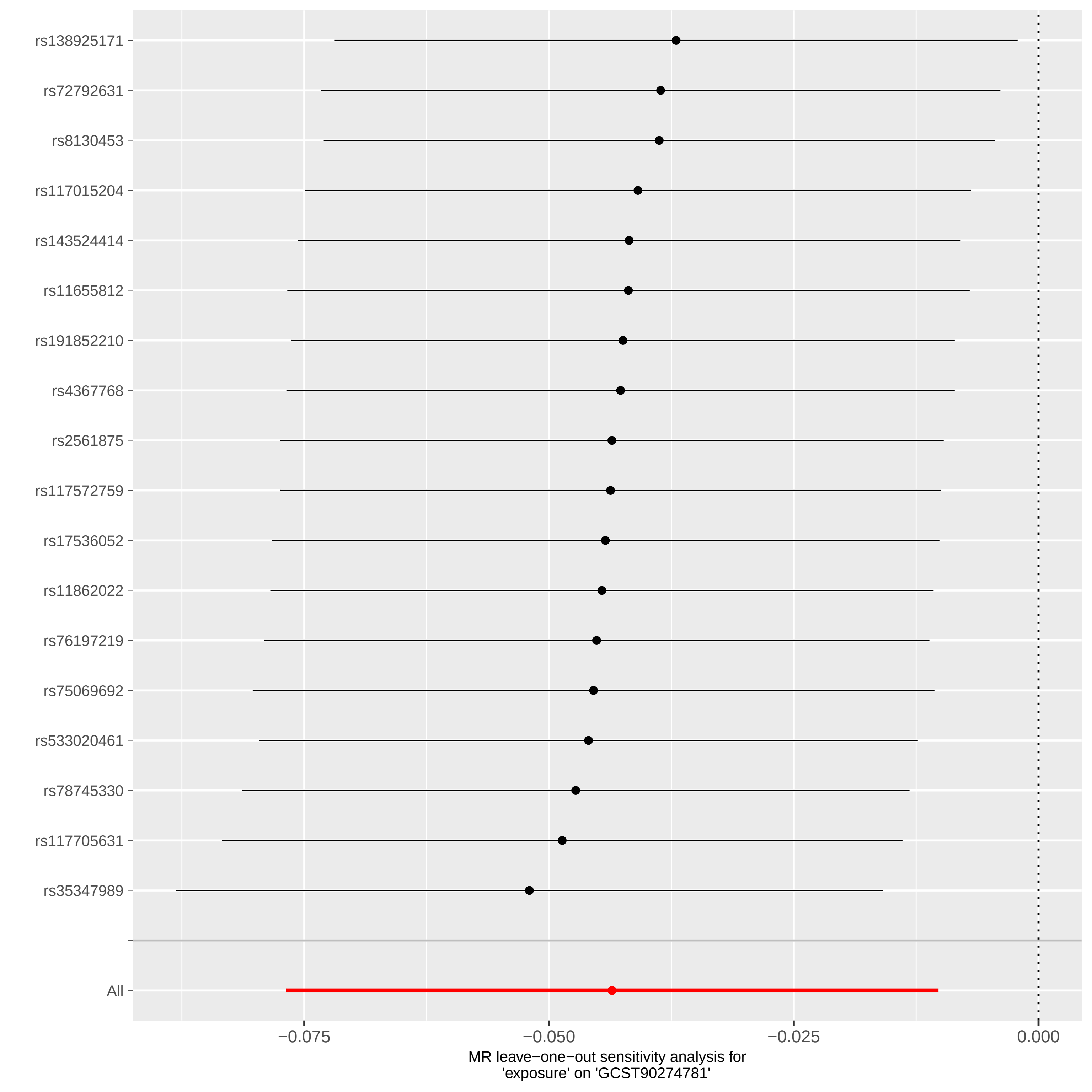

rs138925171
rs72792631
rs8130453
rs117015204
rs143524414
rs11655812
rs191852210
rs4367768
rs2561875
rs117572759
rs17536052
rs11862022
rs76197219
rs75069692
rs533020461
rs78745330
rs117705631
rs35347989
All
−0.075
−0.050
−0.025
0.000
MR leave−one−out sensitivity analysis for
'exposure' on 'GCST90274781'

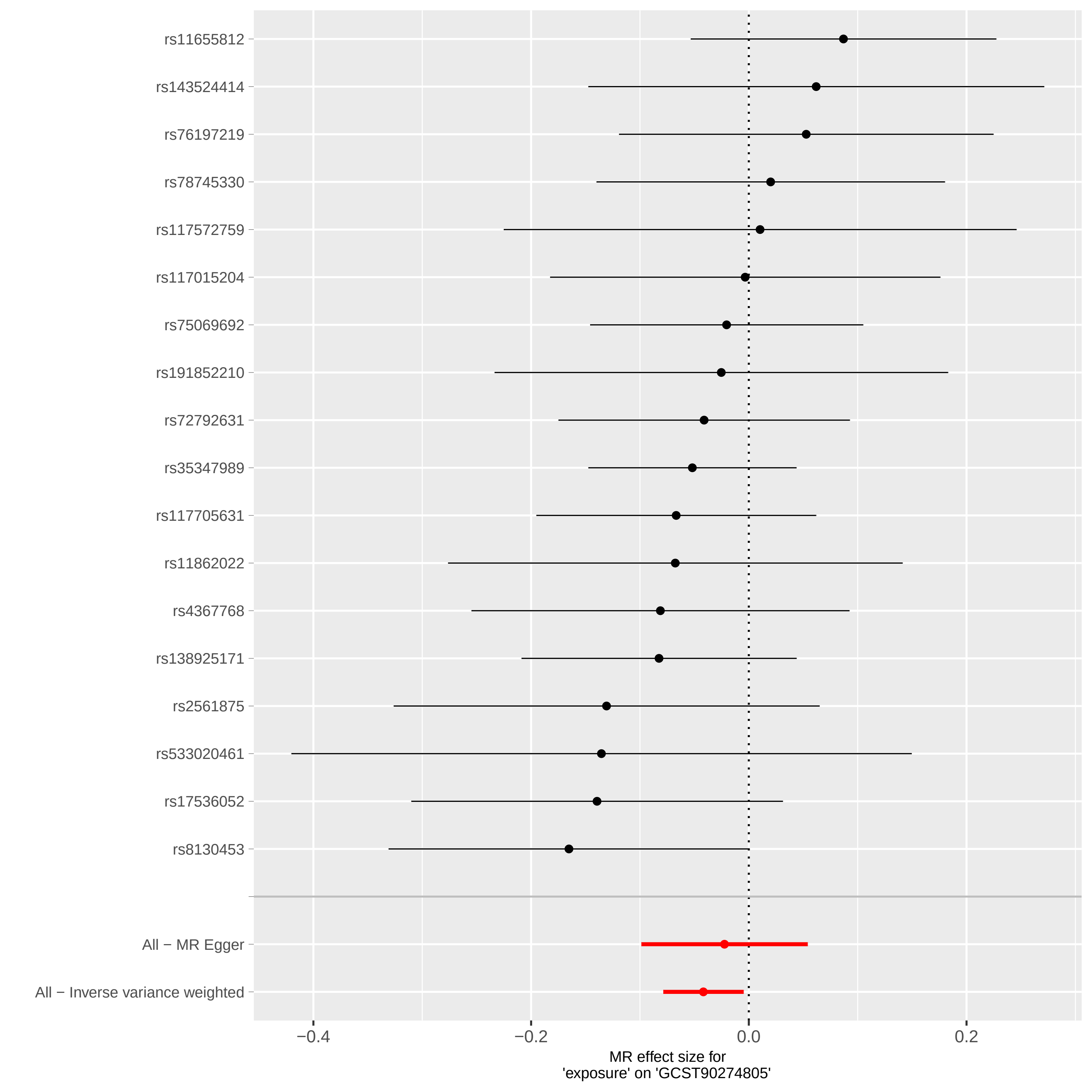

rs11655812
rs143524414
rs76197219
rs78745330
rs117572759
rs117015204
rs75069692
rs191852210
rs72792631
rs35347989
rs117705631
rs11862022
rs4367768
rs138925171
rs2561875
rs533020461
rs17536052
rs8130453
All − MR Egger
All − Inverse variance weighted
−0.4
−0.2
0.0
0.2
MR effect size for
'exposure' on 'GCST90274805'

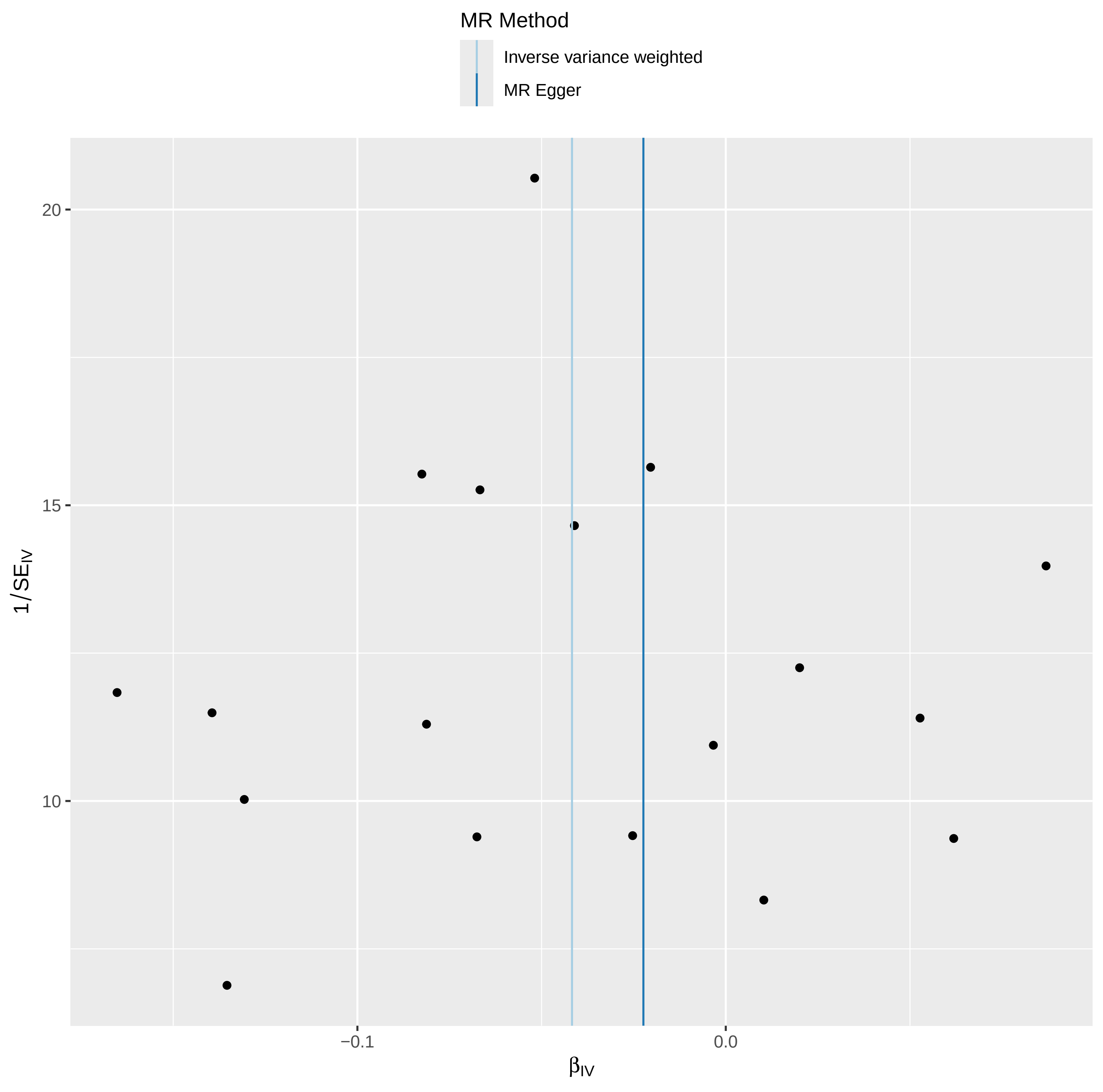
MR Method
Inverse variance weighted
MR Egger
20
15
10
1/SE_{IV}
−0.1
0.0
β_{IV}

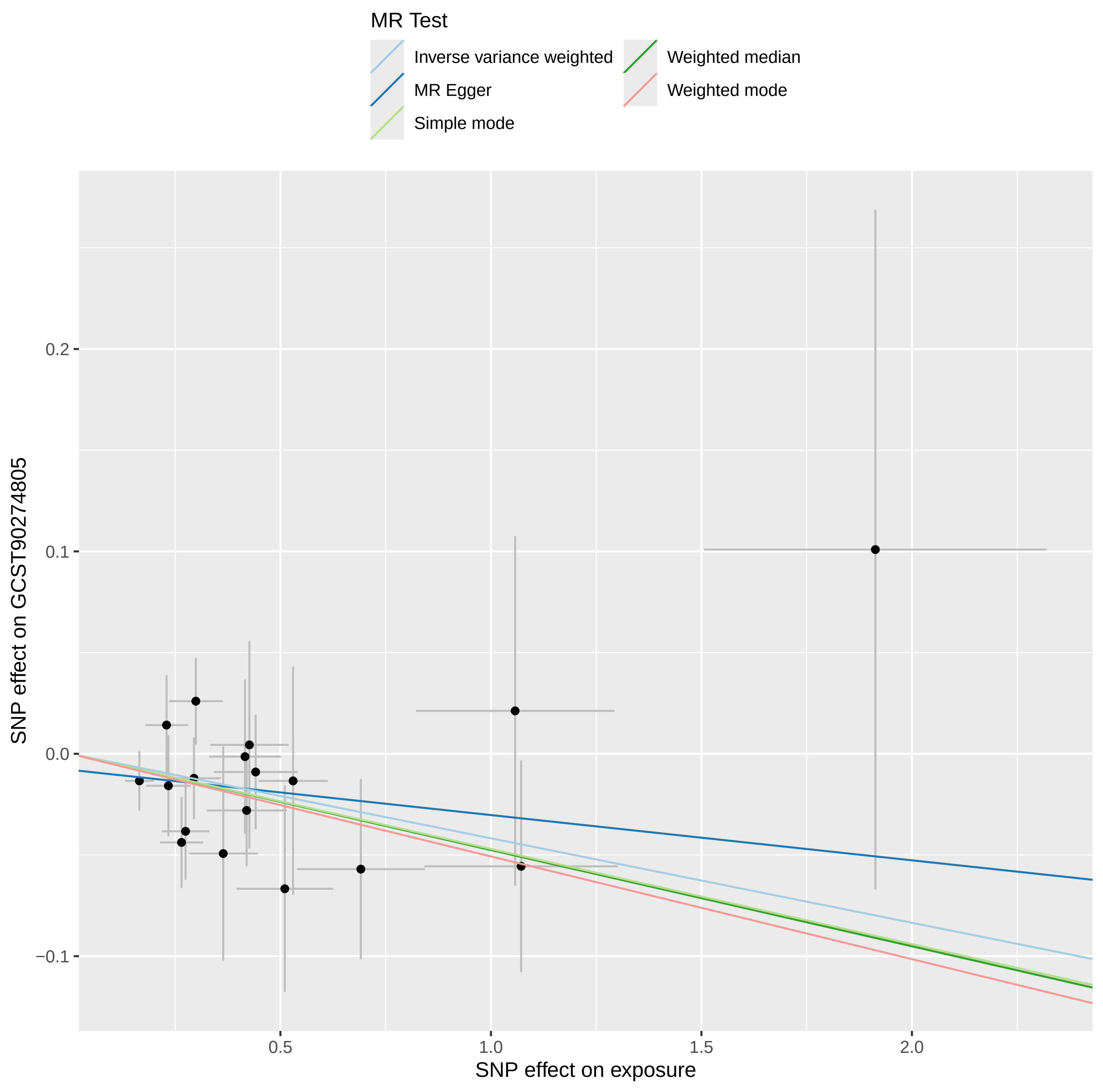

MR Test
Inverse variance weighted
MR Egger
Simple mode
Weighted median
Weighted mode
SNP effect on GCST90274805
0.2
0.1
0.0
−0.1
0.5
1.0
1.5
2.0
SNP effect on exposure

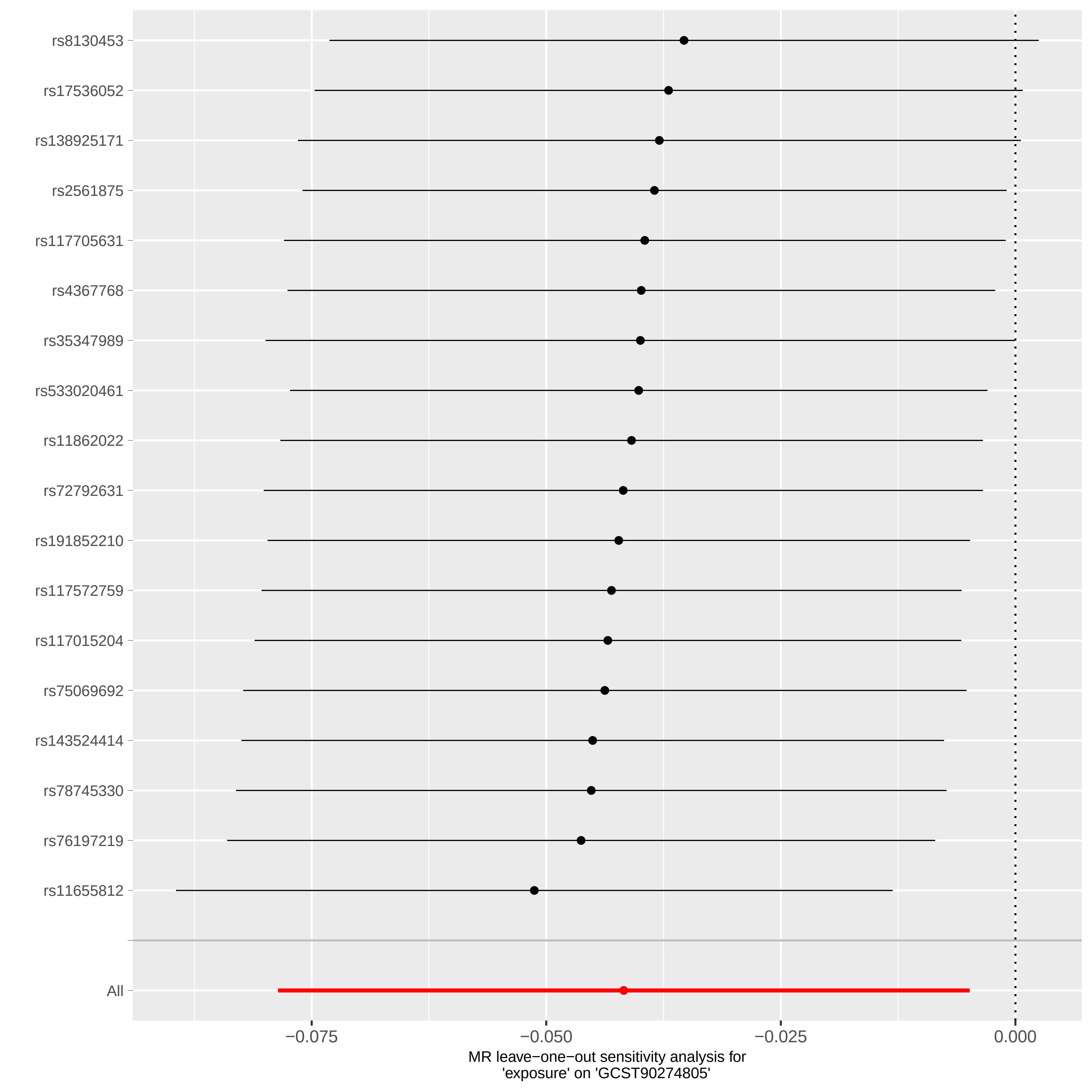

rs8130453
rs17536052
rs138925171
rs2561875
rs117705631
rs4367768
rs35347989
rs533020461
rs11862022
rs72792631
rs191852210
rs117572759
rs117015204
rs75069692
rs143524414
rs78745330
rs76197219
rs11655812
All
−0.075
−0.050
−0.025
0.000
MR leave−one−out sensitivity analysis for
'exposure' on 'GCST90274805'

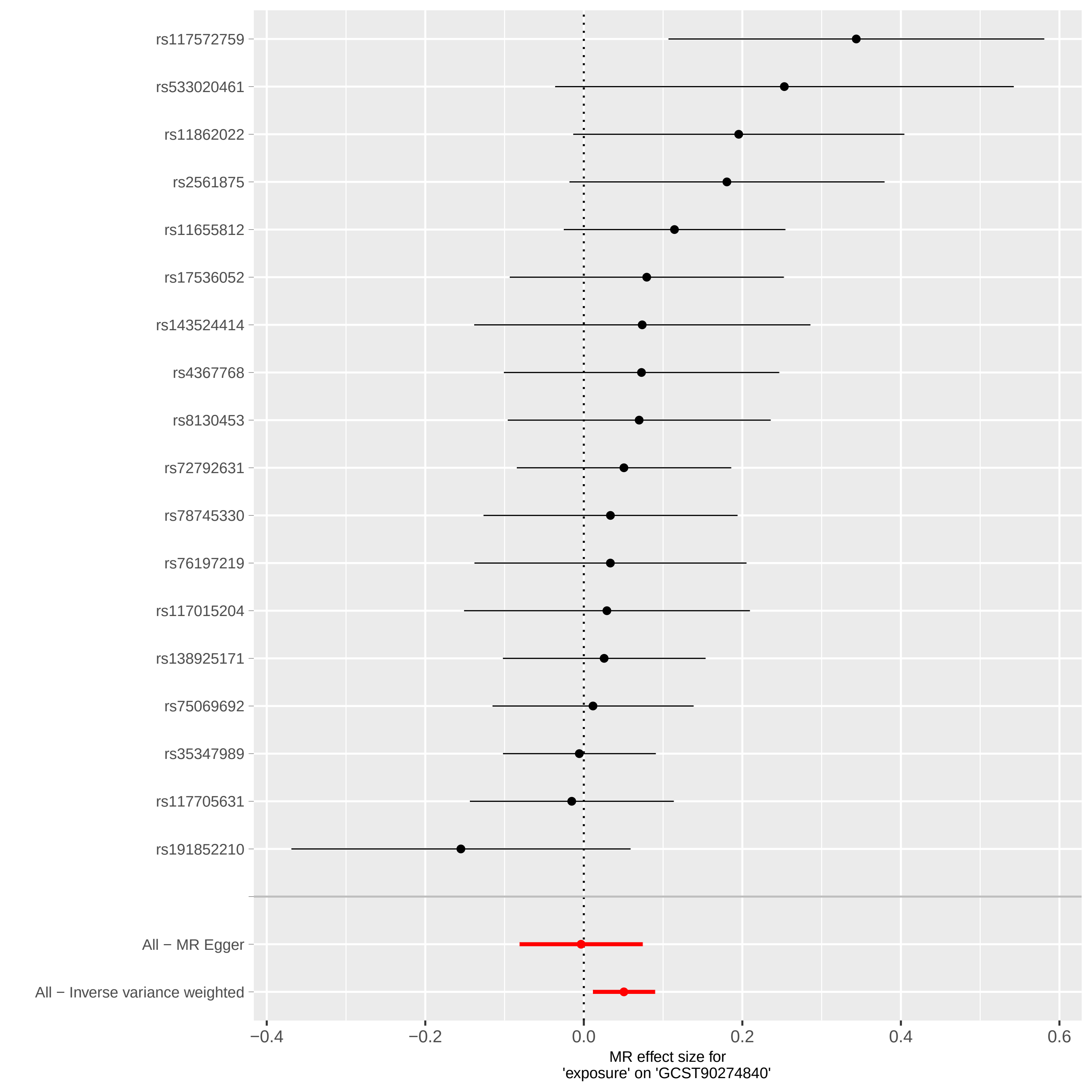

rs117572759
rs533020461
rs11862022
rs2561875
rs11655812
rs17536052
rs143524414
rs4367768
rs8130453
rs72792631
rs78745330
rs76197219
rs117015204
rs138925171
rs75069692
rs35347989
rs117705631
rs191852210
All − MR Egger
All − Inverse variance weighted
−0.4
−0.2
0.0
0.2
0.4
0.6
MR effect size for
'exposure' on 'GCST90274840'

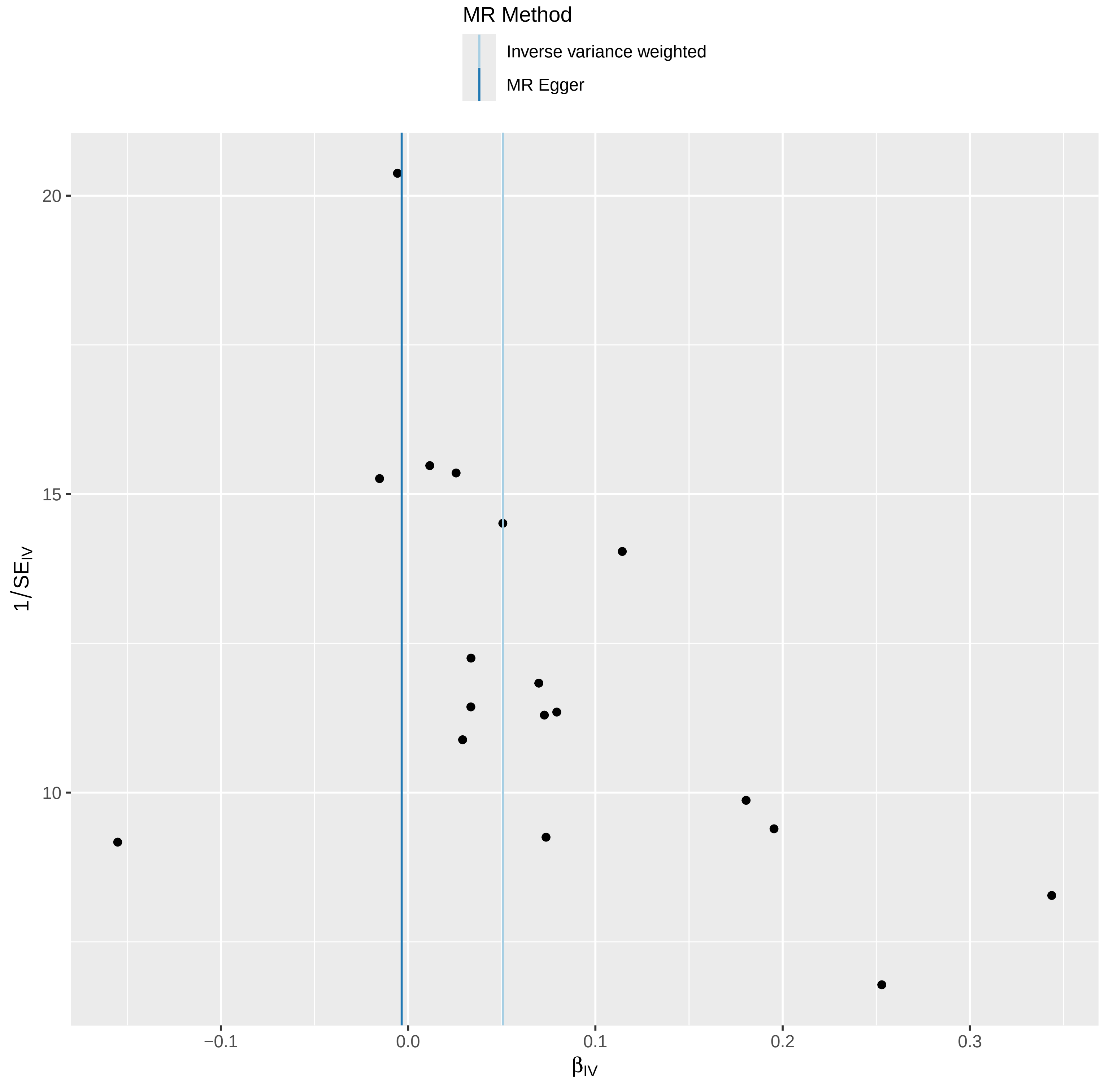
MR Method
Inverse variance weighted
MR Egger
20
15
10
1/SE$_{IV}$
−0.1
0.0
0.1
0.2
0.3
β$_{IV}$

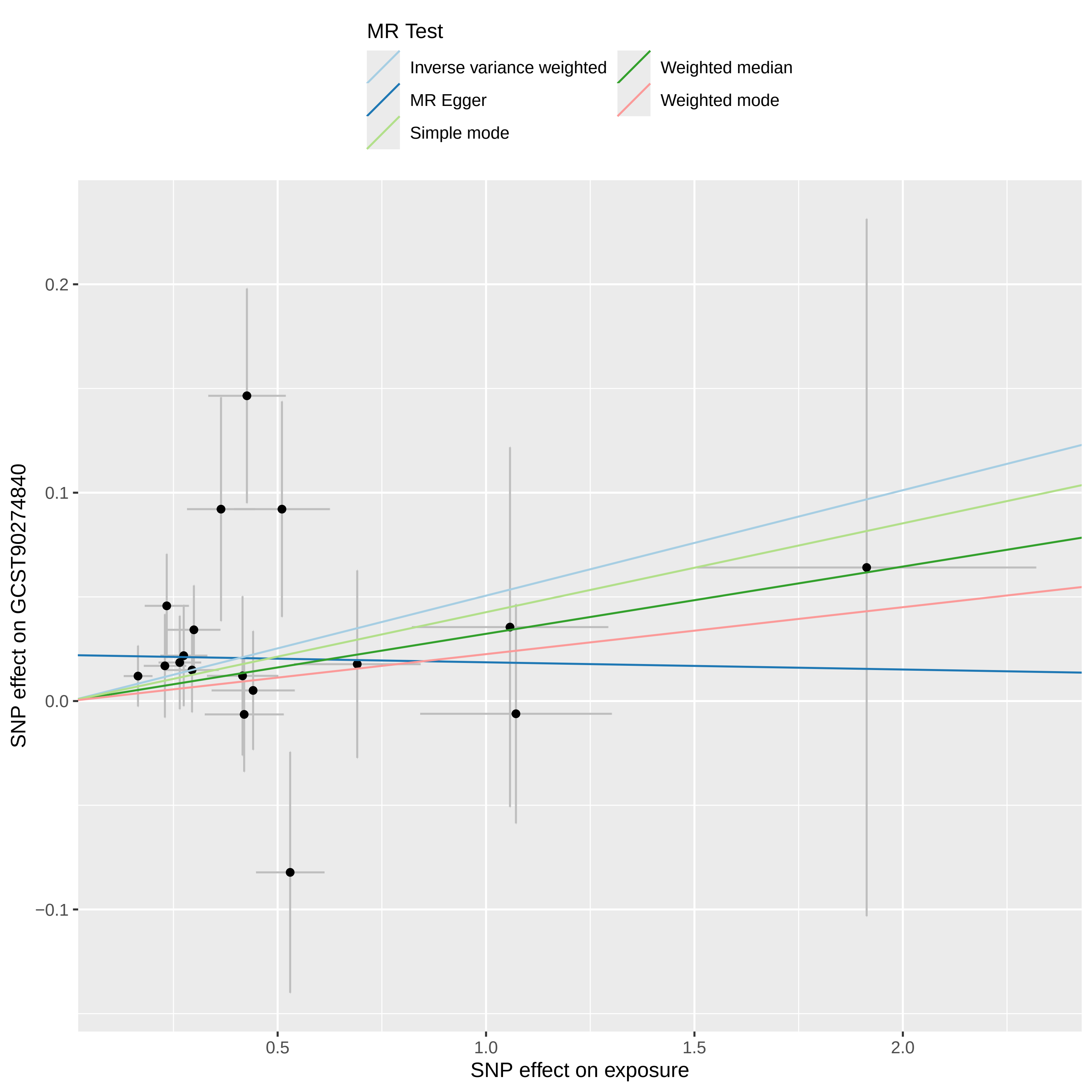
MR Test
Inverse variance weighted
MR Egger
Simple mode
Weighted median
Weighted mode
0.2
0.1
0.0
−0.1
0.5
1.0
1.5
2.0
SNP effect on exposure
SNP effect on GCST90274840

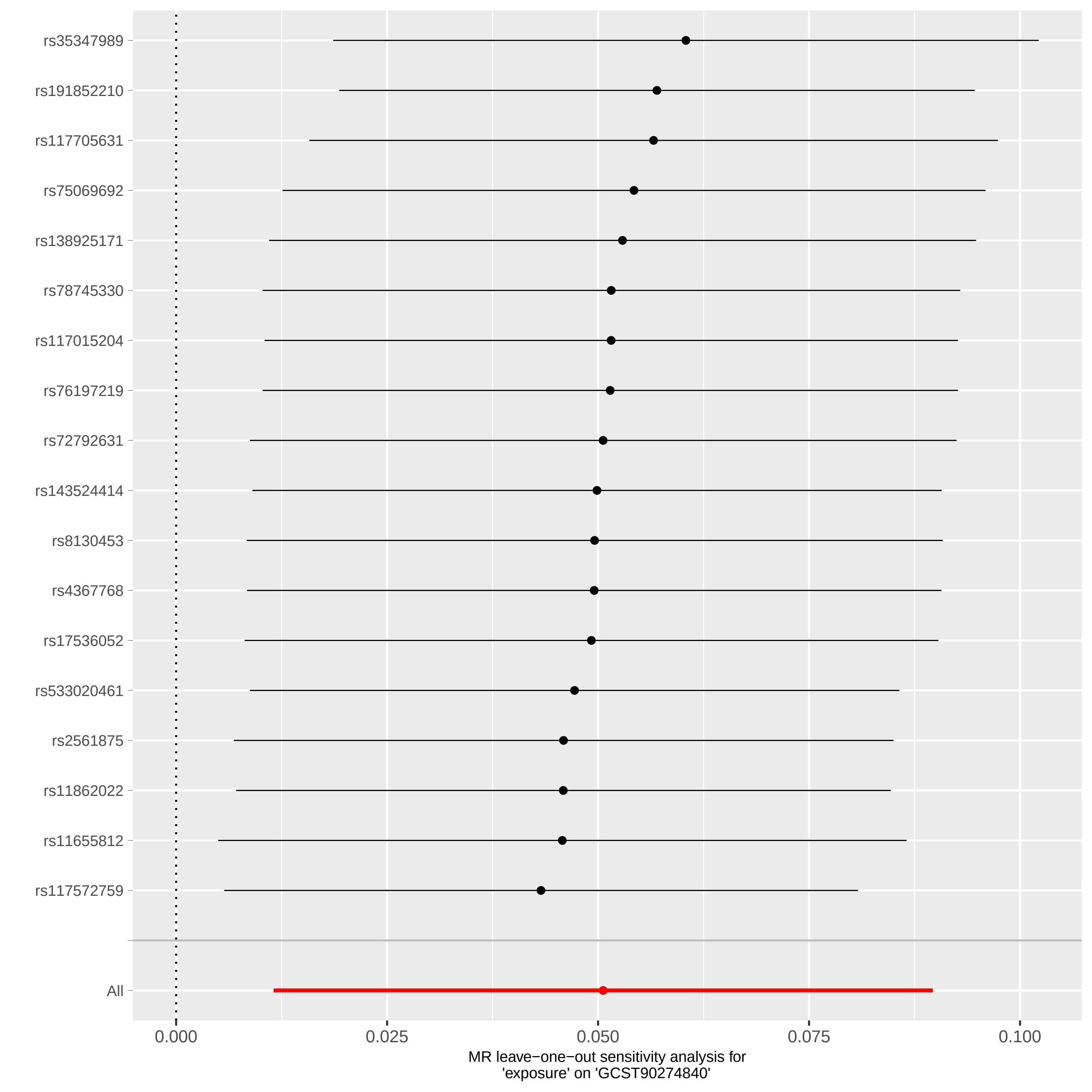
rs35347989
rs191852210
rs117705631
rs75069692
rs138925171
rs78745330
rs117015204
rs76197219
rs72792631
rs143524414
rs8130453
rs4367768
rs17536052
rs533020461
rs2561875
rs11862022
rs11655812
rs117572759
All
0.000
0.025
0.050
0.075
0.100
MR leave−one−out sensitivity analysis for
'exposure' on 'GCST90274840'

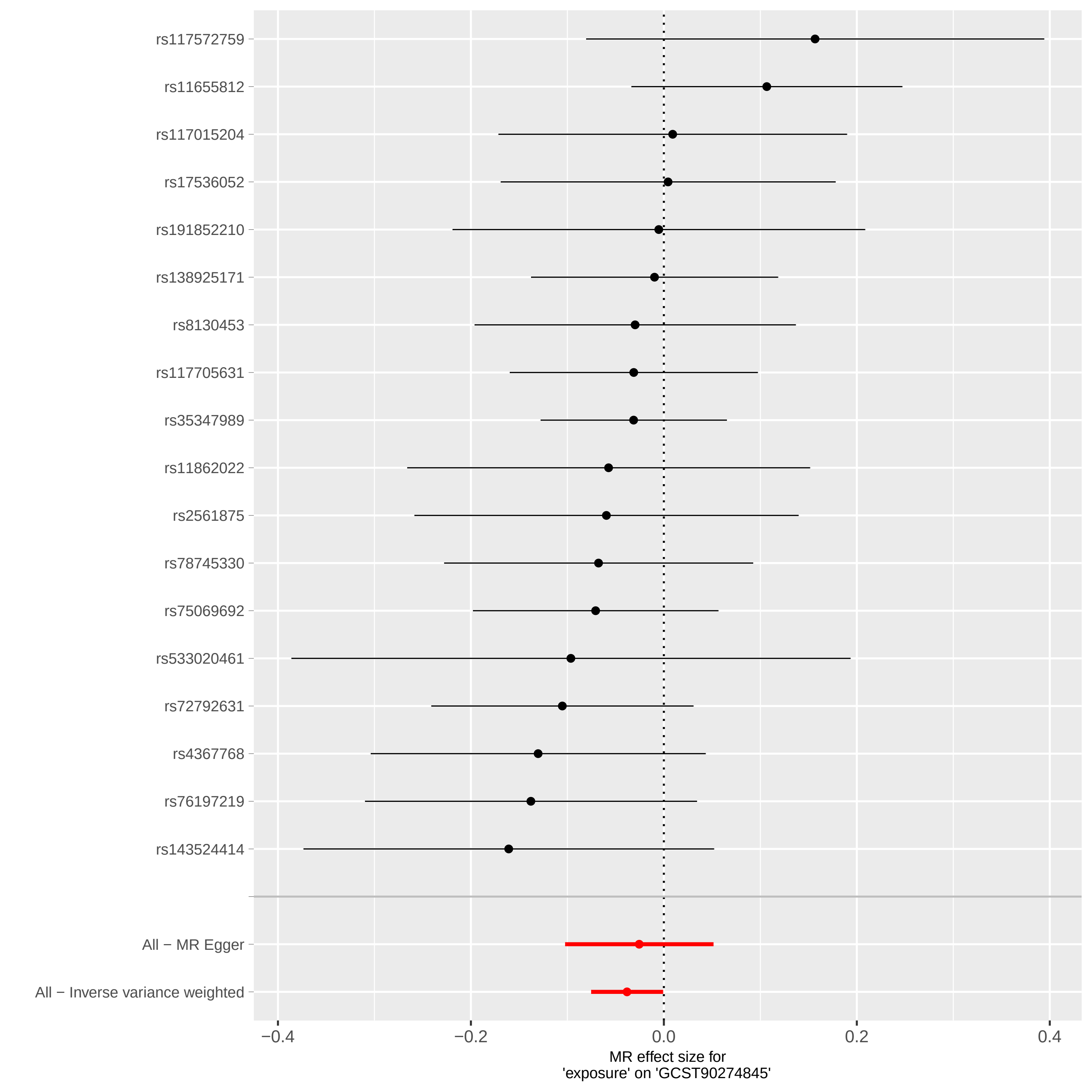

rs117572759
rs11655812
rs117015204
rs17536052
rs191852210
rs138925171
rs8130453
rs117705631
rs35347989
rs11862022
rs2561875
rs78745330
rs75069692
rs533020461
rs72792631
rs4367768
rs76197219
rs143524414
All − MR Egger
All − Inverse variance weighted
−0.4
−0.2
0.0
0.2
0.4
MR effect size for
'exposure' on 'GCST90274845'

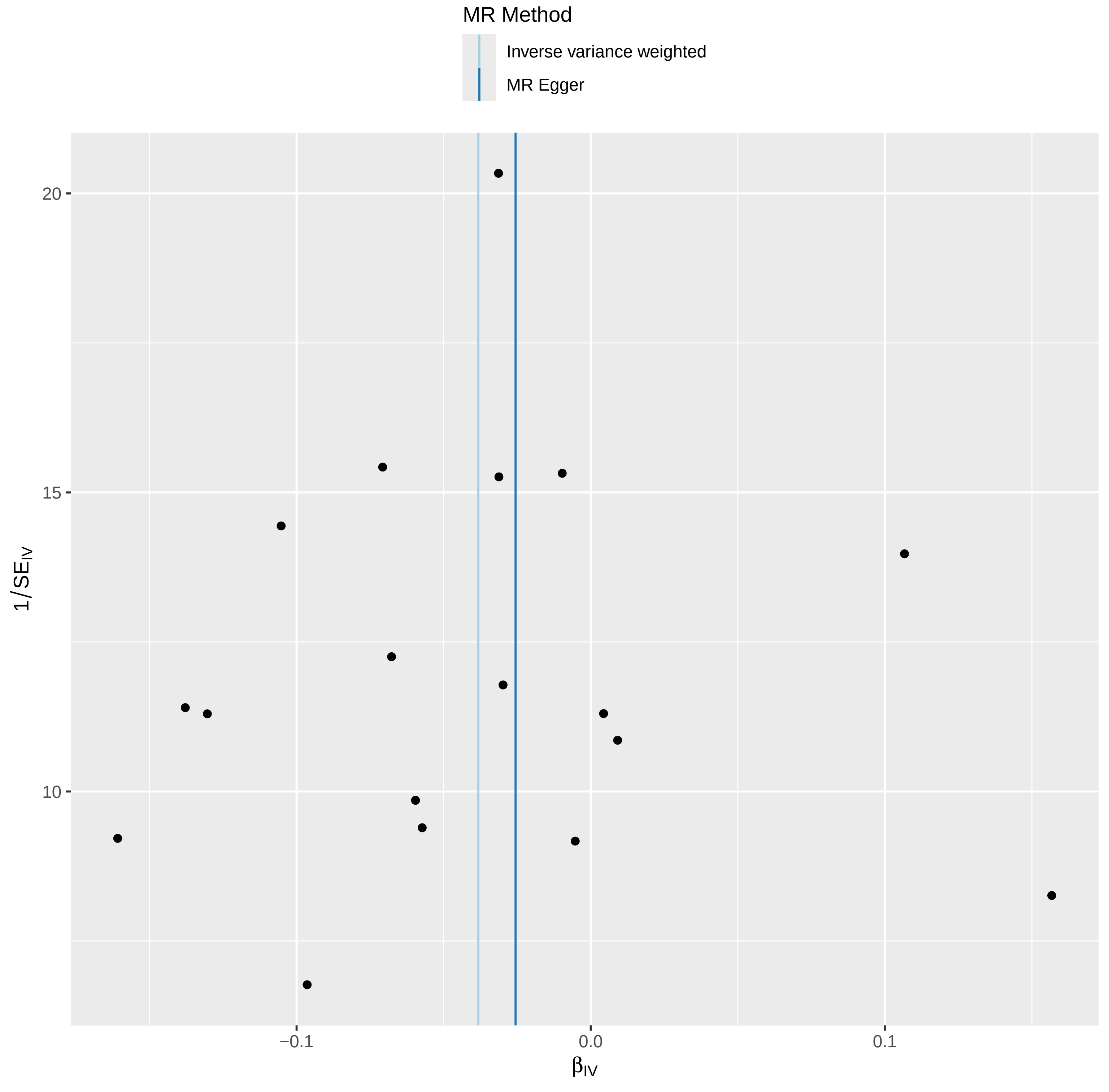

MR Method
Inverse variance weighted
MR Egger
20
15
10
1/SE$_{IV}$
−0.1
0.0
0.1
β$_{IV}$

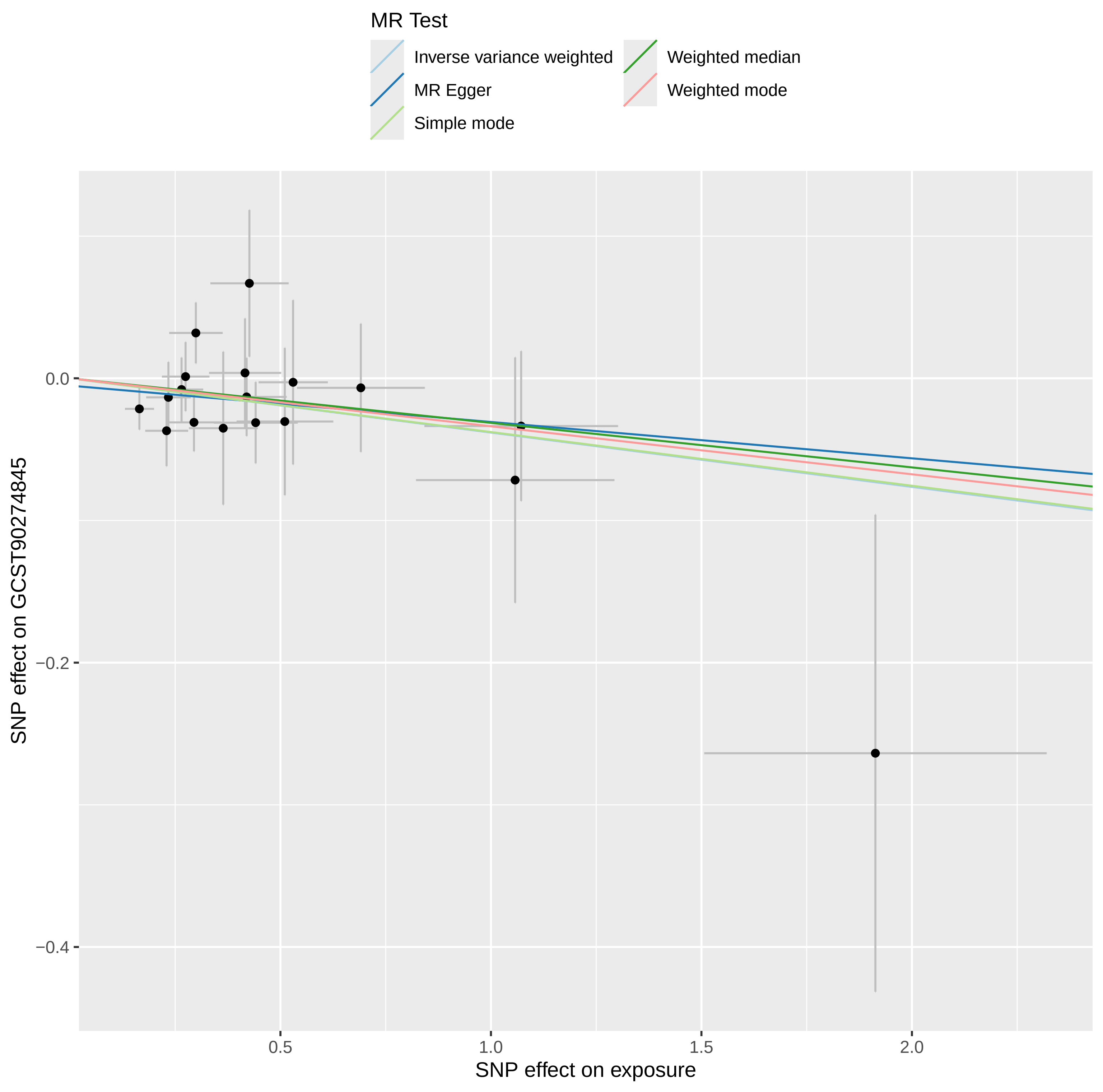

MR Test
Inverse variance weighted
MR Egger
Simple mode
Weighted median
Weighted mode
0.0
−0.2
−0.4
0.5
1.0
1.5
2.0
SNP effect on exposure
SNP effect on GCST90274845

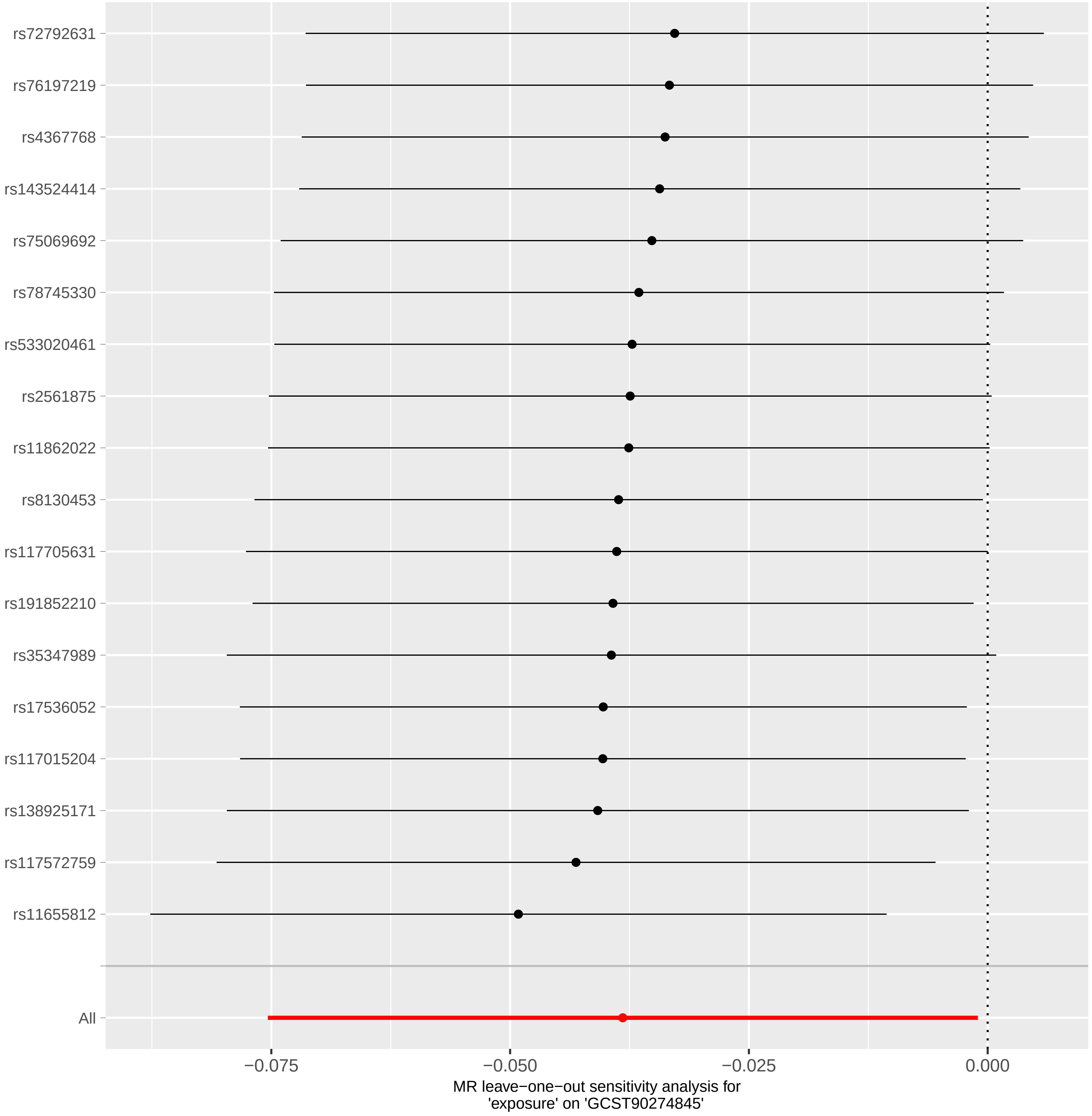
rs72792631
rs76197219
rs4367768
rs143524414
rs75069692
rs78745330
rs533020461
rs2561875
rs11862022
rs8130453
rs117705631
rs191852210
rs35347989
rs17536052
rs117015204
rs138925171
rs117572759
rs11655812
All
−0.075
−0.050
−0.025
0.000
MR leave−one−out sensitivity analysis for
'exposure' on 'GCST90274845'

ID	Mean_F	Median_F	Min_F	Max_F
GCST90274758	28.74596	21.83300	19.59389	126.54532
GCST90274759	397.77850	64.08726	22.82819	1796.08979
GCST90274760	21.55102	21.20602	19.62707	25.17677
GCST90274761	22.11047	21.85769	19.55800	25.78674
GCST90274762	22.82727	21.07996	19.53828	43.12992
GCST90274763	26.66067	21.60850	19.67452	131.89994
GCST90274764	35.23721	21.82313	19.63987	217.17261
GCST90274765	34.98233	21.38615	19.62954	310.83708
GCST90274766	24.30463	21.24697	19.66265	62.18823
GCST90274767	59.22396	21.69468	19.53728	1061.04832
GCST90274768	284.72607	23.84567	22.68830	2892.29049
GCST90274769	23.03521	21.36413	19.51184	33.99007
GCST90274770	72.13979	21.76789	19.55608	982.20806
GCST90274771	34.14806	21.19046	19.53309	317.93331
GCST90274772	80.65051	21.56964	19.58097	1286.12934
GCST90274773	31.76452	22.80295	19.63331	124.26656
GCST90274774	283.75635	23.68231	22.68351	2026.05984
GCST90274775	41.37582	22.49859	19.62331	430.64616
GCST90274776	27.73754	20.98787	19.58885	203.63825
GCST90274777	150.63202	31.51040	22.74936	1934.54814
GCST90274778	24.83301	21.72770	19.68428	47.56868
GCST90274779	42.86206	20.39383	19.54188	546.92707
GCST90274780	30.66746	21.72076	19.50983	152.44301
GCST90274781	30.11135	21.59751	19.62138	213.42805
GCST90274782	72.37365	23.92105	19.67623	782.34757
GCST90274783	292.70769	50.34145	22.98601	1253.46466
GCST90274784	26.31175	20.94837	19.51520	122.88132
GCST90274785	36.37791	21.19702	19.58885	299.83904
GCST90274786	29.06355	21.34080	19.94784	158.70101
GCST90274787	28.85801	21.04896	19.58054	198.11453
GCST90274788	34.79062	22.08754	19.55859	156.61294
GCST90274789	24.58726	21.62766	19.59407	78.80541
GCST90274790	233.77718	23.71759	22.64443	1676.16310
GCST90274791	36.06412	21.67038	19.66716	442.13902

ID	Mean_F	Median_F	Min_F	Max_F
GCST90274792	39.74365	21.07769	19.61219	385.53435
GCST90274793	25.49027	21.15584	19.59310	86.57632
GCST90274794	22.36888	21.36278	19.57009	29.36423
GCST90274795	27.40670	21.47077	19.61912	107.52501
GCST90274796	21.43836	20.98205	19.51519	25.23438
GCST90274797	73.89368	21.63895	19.53507	1444.50742
GCST90274798	141.94895	32.67700	22.61725	1653.77710
GCST90274799	21.71835	20.49843	19.64657	31.33751
GCST90274800	62.64722	21.51941	19.55175	844.96626
GCST90274801	21.73375	21.10657	19.60892	27.58870
GCST90274802	22.82954	20.89084	19.64975	59.59736
GCST90274803	36.17990	21.25937	19.55331	228.09092
GCST90274804	219.83492	27.98710	23.01402	1492.99304
GCST90274805	27.75598	20.96994	19.60879	150.45173
GCST90274806	21.83264	21.57234	19.74282	26.19523
GCST90274807	21.34983	20.72112	19.51413	27.02567
GCST90274808	21.54605	20.75447	19.53211	34.97090
GCST90274809	21.28456	21.25162	19.65297	26.02539
GCST90274810	21.49403	20.91949	19.78443	28.74939
GCST90274811	21.50724	21.34141	19.53219	25.39165
GCST90274812	21.03965	20.75854	19.51125	25.81708
GCST90274813	21.31811	20.46124	19.56877	27.35988
GCST90274814	21.62430	20.80925	19.57240	27.07786
GCST90274815	35.53237	21.34939	20.01322	200.23101
GCST90274816	21.85253	20.93901	19.62421	25.66194
GCST90274817	23.78384	21.64633	19.61975	61.75426
GCST90274818	28.11355	21.83609	19.56771	190.41415
GCST90274819	21.05302	20.72968	19.56028	26.33274
GCST90274820	34.98820	21.13041	19.54309	355.49628
GCST90274821	37.92671	21.59901	19.59529	230.14975
GCST90274822	626.23009	23.62027	23.11828	3549.33429
GCST90274823	29.66127	21.84615	19.69501	158.11376
GCST90274824	51.06500	21.68587	19.53269	274.82418
GCST90274825	73.24681	21.35646	19.71316	923.13031

ID	Mean_F	Median_F	Min_F	Max_F
GCST90274826	32.35462	21.45594	19.75032	148.00769
GCST90274827	58.64166	21.17318	19.63834	585.24899
GCST90274828	21.36499	20.98521	19.51702	25.48082
GCST90274829	22.23048	20.89503	19.51637	50.71531
GCST90274830	35.90391	22.18803	19.51361	223.65482
GCST90274831	27.00161	21.60393	19.53623	88.54090
GCST90274832	26.59718	21.97800	19.70218	116.44426
GCST90274833	37.99968	22.04695	19.64737	393.85875
GCST90274834	27.24059	21.43792	19.57311	124.16496
GCST90274835	30.26535	21.77956	19.56948	185.90987
GCST90274836	31.18991	21.70847	19.59236	194.30999
GCST90274837	22.13521	20.95527	19.65439	26.67044
GCST90274838	23.77167	21.60273	19.54226	55.43021
GCST90274839	21.97276	21.27553	19.55946	26.61152
GCST90274840	171.93140	50.17411	22.64714	1027.42069
GCST90274841	25.30314	22.42864	19.90655	100.13691
GCST90274842	32.15720	21.31326	19.64253	321.31350
GCST90274843	54.59660	22.89369	19.60329	475.33395
GCST90274844	34.90350	21.66769	19.54278	211.36422
GCST90274845	22.08537	21.56662	19.66799	30.97255
GCST90274846	30.82370	21.19187	19.55501	267.50169
GCST90274847	30.74928	20.58816	19.51978	184.09635
GCST90274848	69.69739	22.09640	19.63140	1476.94326
